# New monoterpene phenyl ethers from *Illicium micranthum*

**DOI:** 10.1007/s13659-013-0007-x

**Published:** 2013-03-16

**Authors:** Zheng-Ye Guan, Chuan-Fu Dong, Li Gao, Jia-Ping Wang, Shi-De Luo, Yi-Fen Wang

**Affiliations:** 17 State Key Laboratory of Phytochemistry and Plant Resources in West China, Kunming Institute of Botany, Chinese Academy of Sciences, Kunming, 650201 China; 27 University of Chinese Academy of Sciences, Beijing, 100049 China

**Keywords:** *Illicium micranthum*, monoterpene phenyl ethers, micranthumnins, anti-AChE activities

## Abstract

Seven new monoterpene phenyl ethers, namely micranthumnins A-G (**1–7**), were isolated from the stem bark of *Illicium micranthum* (Illiciaceae). Their structures were elucidated by comprehensive spectroscopic analyses including MS, IR, 1D and 2D NMR. All compounds were evaluated for their anti-AChE activities.

**Electronic Supplementary Material:**

Supplementary material is available for this article at 10.1007/s13659-013-0007-x and is accessible for authorized users.

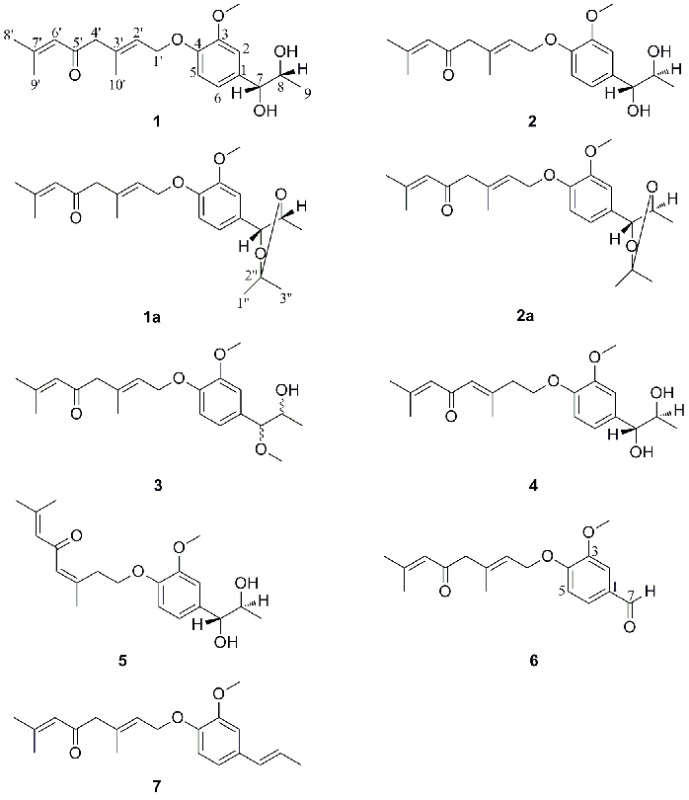

## Introduction

*Illicium* species, which belong to the only genus of the family Illiciaceae, are mainly distributed in East Asia and the southeast of North America.[1] Twenty eight species (over 60% of the family) in Illiciaceae are mainly distributed in southern and eastern China, and twelve species of *Illicium* L. are found in Yunnan province.[2,3] Phytochemical investigation showed that this genus produced monoterpenoids,[4] sesquiterpene lactones,[5–8] diterpenoids,[9] triterpenoids,[10] lignans and neolignans,[11–13] which showed various bioactivities, such as insecticidal activity,[14] cancer chemopreventive activity,[15] and neurotrophic activity.[16–18] *Illicium micranthum* was a poisonous shrub used as a traditional pesticide.[3] Previous studies on this plant have resulted in the isolation of eight secoprezizaane sesquiterpene lactones,[5] seven phenylpropanoids[19] and several other compounds.[20] As an on-going search for neurotrophic active compounds from natural resources, our investigation on *I. micranthum* led to the isolation of seven new monoterpene phenyl ethers (**1**–**7**). This paper deals with the isolation, structure characterization, and anti-AChE activity of these compounds.

## Results and Discussion

A 90% aqueous MeOH extract the stem bark of *I. micranthum* was partitioned between $CHCl_3$ and $H_2O$. The $CHCl_3$ solubles were dried and subjected to silica gel, Sephadex LH-20 and RP-18 gel column chromatography (CC) and semipreparative HPLC to afford seven new compounds.

The molecular formula of micranthumnin A (**1**) was assigned as $C_{20}H_{28}O_5$ on the basis of HREIMS at *m/z* 348.1931 (calcd for 348.1937, $[M]^+$), indicating 7 degrees of unsaturation.

**Figure 1.** Structures of compounds **1**–**7**, **1a** and **2a**

Its IR spectrum showed the presence of aromatic ring (1602, 1516, 1424 $cm^{-1}$) and one *α*,*β*-conjugated carbonyl (1673 $cm^{-1}$). The $^1H$ NMR spectrum of **1** (Table 1) revealed the presence of one 1,3,4-trisubstituted aromatic ring [$\delta_H$ 7.03 (1H, d, $J$ = 1.8 Hz, H-2), 6.91 (1H, d, $J$ = 8.4 Hz, H-5), 6.87 (1H, dd, $J$ = 8.4, 1.8 Hz, H-6)], one methoxy group at $\delta_H$ 3.84 (3H, s, $OCH_3$-3), four methyls [$\delta_H$ 1.12 (3H, d, $J$ = 6.6 Hz, H-9), 1.91

*To whom correspondence should be addressed. E-mail: wangyifen@mail.kib.ac.cn

**Table 1.** $^{1}$**H NMR (600 MHz) data of compounds 1 and 2 in** $CD_3OD$ **(*δ* in ppm, *J* in Hz)**

| pos. | 1 | 2 |
|---|---|---|
| 2 | 7.03 (d, 1.8) | 6.98 (d, 1.8) |
| 5 | 6.91 (d, 8.4) | 6.91 (d, 8.4) |
| 6 | 6.87 (dd, 8.4, 1.8) | 6.86 (dd, 8.4, 1.8) |
| 7 | 4.43 (d, 4.8) | 4.28 (d, 7.2) |
| 8 | 3.84 (overlapped) | 3.77 (dq, 7.2, 6.6) |
| 9 | 1.12 (d, 6.6) | 0.96 (d, 6.6) |
| 1′ | 4.61 (d, 6.6) | 4.62 (d, 6.6) |
| 2′ | 5.61 (td, 6.6, 1.2) | 5.60 (td, 6.6, 1.2) |
| 4′ | 3.16 (s) | 3.16 (s) |
| 6′ | 6.22 (br. s) | 6.22 (br. s) |
| 8′ | 1.91 (d, 1.2) | 1.90 (d, 1.2) |
| 9′ | 2.13 (d, 1.2) | 2.13 (d, 0.6) |
| 10′ | 1.72 (s) | 1.71 (s) |
| $OCH_3$-3 | 3.84 (s) | 3.84 (s) |

(3H, d, $J$ = 1.2 Hz, H-8′), 2.13 (3H, d, $J$ = 1.2 Hz, H-9′), 1.72 (3H, s, H-10′)], two olefinic protons at [$\delta_H$ 5.61 (1H, td, $J$ = 6.6, 1.2 Hz, H-2′), 6.22 (1H, br. s, H-6′)], and two oxymethines [$\delta_H$ 4.43 (1H, d, $J$ = 4.8 Hz, H-7), 3.84 (1H, m, H-8)]. The proton and carbon signals were assigned from the $^{1}H$-$^{1}H$ COSY, HMQC and HMBC spectroscopic data (see Table 1). Comparison of the NMR data between **1** and tomentellin[21] revealed that compound **1** was structurally similar to tomentellin[21]. The difference was the methyl ether moiety at $\delta_C$ 166.8 (C-7′), 52.0 ($OCH_3$-7′) in tomentellin[21] was replaced by a 1,2-propanediol moiety at $\delta_C$ 77.4 (C-7), 71.3 (C-8), and 17.5 (C-9) in **1**. This was further confirmed by the HMBC correlations (Figure 2) from H-7 at $\delta_H$ 4.43 to C-1 ($\delta_C$ 133.2), C-2 ($\delta_C$ 109.9), C-6 ($\delta_C$ 118.9). Moreover, the HMBC correlation from H-1′ at $\delta_H$ 4.61 to C-4 ($\delta_C$ 147.6), combined with the ROESY correlations (Figure 4) of H-1′/H-5 and -$OCH_3$/H-2 suggested that the monoterpene moiety was connected to C-4 of the aromatic ring through an ether bond. The *E*-zeometry at C-2′ and C-3′ was assigned by the ROESY correlations of H-2′/H-4′ and H-1′/H-10′.

Micranthumnin B (**2**) was obtained as a yellow gum with the same molecular formula as **1**. Its $^{13}C$ NMR spectrum (Table 3) was very similar to that of **1** except the signals of C-7, C-8, and C-9 shifted from $\delta_C$ 77.4, 71.3 and 17.5 to $\delta_C$ 79.2, 72.1 and 18.7, respectively. Moreover, the $^{1}H$ NMR signals (Table 1) of H-7, H-8 and H-9 shifted from $\delta_H$ 4.43, 3.84 and 1.12 to $\delta_H$ 4.28, 3.77, 0.96. Detailed analysis of 2D NMR spectra revealed that the structure of **2** was also 4-[(2′*E*)-3′,7′-dimethyl-5′-oxo-2′,6′-octadienyl)oxy]-3-methoxy-phenylpropane-7,8-diol. The similarity of the NMR spectra between **1** and **2** suggested that they might be *erythro* and *threo* isomers. The OH configurations of two stereogenic centers in compounds **1** and **2** were deduced by comparison with the $^{13}C$ NMR spectra of similar compounds, *erythro*- and *threo*-1-phenyl-1,2-dihydroxypropane, for which the structures were confirmed by synthesis[22,23]. The $^{13}C$ NMR signals of C-7 and C-8 in *erythro*-1-phenyl-1,2-dihydroxypropane ($\delta_C$ 77.6 and 71.3) were closer together than those in the *threo* isomer ($\delta_C$ 79.5 and 72.7). In compound **1**, C-7 and C-8 showed signals at $\delta_C$ 77.4 and 71.3, which were closer than those of compound **2** ($\delta_C$ 79.2 and 72.1).

The relative configurations of **1** and **2** were further confirmed by the derivative reaction. In the reactions, the vicinal diol parts in **1** and **2** react with DMP to yield ketal products **1a** and **2a**, respectively, which makes the carbon-carbon bond between C-7 and C-8 rotate unfreely. The relative configurations of **1** and **2** were determined depending on the two ketal products **1a** and **2a** whose relative configurations were clarified by ROESY experiments (Figure 3). ROESY correlations of H-7/H-1″ and H-8/H-1″ were detected while the correlation of H-7/H-9 was disappeared in **1a**, which determined the *erythro* configuration of **1a**. The relative configuration of **2a** was determined to be *threo* by ROESY correlations of H-7/H-1″, H-8/H-3″, and H-7/H-9. Consequently, the relative configurations of **1** and **2** were determined to be *erythro* and *threo*, respectively (Figure 1).

**Figure 2.** Selected $^{1}H$-$^{1}H$ COSY and HMBC correlations of **1**, **3**, **4**, **6** and **7**

**Figure 3.** Selected ROESY correlations of **1a** and **2a**

The mass spectrum of compound **3** (HREIMS $m/z$ 362.2098, clacd for 362.2093, $[M]^+$) was 14 amu higher than that of **1**, indicating that there was probably one more methyl group in **3**. The similarities of the spectroscopic data (Tables 2 and 3) between **3** and **1** suggested that **3** was identical to **1** except for the methoxyl group at C-7, causing a significant downfield chemical shift from $\delta_C$ 77.4 to $\delta_C$ 89.2 due to C-7. HMBC correlation (Figure 2) from $OCH_3$-7 at $\delta_H$ 3.23 to C-7 at $\delta_C$ 89.2 further confirmed the above assignment. Without an isomer as a comparison, as well as the significant difference of C-7 chemical shift between **3** and **1**, the relative configuration of **3** was not clarified. Finally, the structure of **3** was established as shown in Figure 1, and named as micranthumnin C.

Micranthumnin D (**4**), yellow gum, had the same molecular formula ($C_{20}H_{28}O_5$) as **1**. Analysis of its NMR data (Tables 2 and 3) showed that **4** was similar to **1**, except for the signals of one methylene, one oxymethylene, one olefinic methine and

**Table 2. $^1$H NMR data of compounds 3–7 ($\delta$ in ppm, $J$ in Hz)**

| pos. | 3[b] | 4[a] | 5[a] | 6[a] | 7[a] |
|---|---|---|---|---|---|
| 2 | 6.79 (s) | 7.01(d, 2.4) | 6.97(d, 1.8) | 7.47 (d, 1.8) | 6.96 (d, 1.8) |
| 5 | 6.85 (d, 8.3) | 6.96 (d, 8.4) | 6.95 (d, 8.4) | 7.15 (d, 8.4) | 6.84 (d, 8.4) |
| 6 | 6.78 (d, 8.3) | 6.90 (dd, 8.4, 2.4) | 6.86 (dd, 8.4, 1.8) | 7.54 (dd, 8.4, 1.8) | 6.82 (dd, 8.4, 1.8) |
| 7 | 3.77 (d, 8.0) | 4.31 (d, 7.2) | 4.28 (d, 7.2) | 9.83 (s) | 6.32 (dq, 15.6, 1.2) |
| 8 | 3.80 (overlapped) | 3.81 (overlapped) | 3.80 (overlapped) | | 6.13 (dq, 15.6, 6.6) |
| 9 | 0.96 (d, 5.6) | 0.98 (d, 6.0) | 0.96 (d, 6.6) | | 1.85 (dd, 6.6, 1.2) |
| 1′ | 4.66 (d, 6.0) | 4.18 (t, 6.6) | 4.16 (t, 6.6) | 4.78 (d, 6.0) | 4.61 (d, 6.0) |
| 2′ | 5.64 (t, 6.0) | 2.65 (t, 6.6) | 3.05 (t, 6.6) | 5.64 (t, 6.0) | 5.60 (td, 6.0, 1.2) |
| 4′ | 3.14 (s) | 6.25 (d, 1.2) | 6.23 (d, 0.6) | 3.21(s) | 3.14 (s) |
| 6′ | 6.11 (br. s) | 6.17 (br. s) | 6.17 (br. s) | 6.23 (br. s) | 6.18 (br. s) |
| 8′ | 1.88 (s) | 1.94 (d, 1.2) | 2.14 (d, 1.2) | 1.91 (s) | 1.87 (d, 1.2) |
| 9′ | 2.14 (s) | 2.16 (d, 1.2) | 1.91 (d, 1.2) | 2.15 (s) | 2.12 (d, 1.2) |
| 10′ | 1.75 (s) | 2.23 (d, 1.2) | 2.04 (d, 0.6) | 1.79 (s) | 1.71 (s) |
| $OCH_3$-3 | 3.88 (s) | 3.85 (s) | 3.82 (s) | 3.92 (s) | 3.83 (s) |
| $OCH_3$-7 | 3.23 (s) | | | | |

[a]Recorded in $CD_3OD$ at 600MHz; [b]Recorded in $CDCl_3$ at 400MHz.

**Figure 4.** Selected ROESY correlations of **1**, **4** and **5**

one quaternary carbon shifted from $\delta_C$ 55.0 (C-4′), 65.6 (C-1′), 124.5 (C-2′), 134.7 (C-3′) to $\delta_C$ 41.6, 68.4, 128.4, 155.9, suggesting that the double bond between C-2′ and C-3′ may have migrated. $^1$H-$^1$H COSY, HSQC and HMBC analyses of **4** showed the presence of a $CH_2$ (C-1′)-$CH_2$ (C-2′) unit. HMBC correlations (Figure 2) from H-2′ at $\delta_H$ 2.65 to C-1′, C-3′, C-4′ and from H-4′ at $\delta_H$ 6.25 to C-3′, C-5′ indicated the presence of a trisubstituted double bond between C-3′ and C-4′. The *E*-geometry was established by the ROESY correlation (Figure 4) of H-4′/H-2′. Consequently, the structure of **4** was established as shown in Figure 1 and named as micranthumnin D.

Micranthumnin E (**5**) showed an $[M]^+$ ion at *m*/*z* 348.1932 (calcd for 348.1937) in the HREIMS spectrum, indicating a molecular formula of $C_{20}H_{28}O_5$. HMBC correlations in combination with HSQC and $^1$H-$^1$H COSY spectra indicated that **5** had the same planar structure as **4**. A significant chemical shift of C-2′ ($\delta_C$ 34.9) and C-10′ ($\delta_C$ 26.9) might suggest a different geometry of the double bond between C-3′ and C-4′. ROESY correlation (Figure 4) of H-4′/H-10′ was observed, which was absent in **4**, while correlation of H-2′/H-4′ disappeared in **5**, indicating a *Z*-geometry double bond between C-3′ and C-4′. The chemical shifts of C-7 and C-8 in **4** and **5** were very similar (**4**, $\delta_C$ 80.1, 73.0; **5**, $\delta_C$ 80.2, 73.0), and both similar to the *threo*-1-phenyl-1,2-dihydroxypropane ($\delta_C$ 79.5, 72.7). Consequently, the relative configurations of **4** and **5** were established as shown in Figure 1.

The molecular formula of **6** was determined as $C_{18}H_{22}O_4$ by HREIMS (*m*/*z* 302.1512, calcd for 302.1518, $[M]^+$). Comparison of its NMR data (Tables 2 and 3) with **1** suggested that the 1,2-propanediol group in **1** was replaced by a formyl group at C-1. The HMBC correlations (Figure 2) from H-7 ($\delta_H$ 9.83) to C-1 ($\delta_C$ 131.5), C-2 ($\delta_C$ 110.7), and C-6 ($\delta_C$ 127.7), from H-2 ($\delta_H$ 7.47) and H-6 ($\delta_H$ 7.54) to C-7 ($\delta_C$ 192.9) further confirmed this assignment. Thus, **6** was determined and named as micranthumnin F.

The mass spectrum showed that compound **7** was 34 mass amu lower than that of **1**, indicating the probable loss of two hydroxyl groups in **7**. The NMR data indicated that **7** was similar to **1**. Extensive analyses of its 1D and 2D NMR data suggested that the 1,2-propanediol group in **1** was replaced by a propenyl group. The signals corresponding to a propenyl group at $\delta_H$ 6.32 (1H, dq, $J$ = 15.6, 1.2 Hz, H-7), 6.13 (1H, dq, $J$ = 15.6, 6.6 Hz, H-8), and 1.85 (3H, dd, $J$ = 6.6, 1.2 Hz, H-9) were evident in the $^1$H NMR data (Table 2) of **7**. The structure was further confirmed by HMBC correlations (Figure 2) of H-7/C-1, C-2, C-6 and H-8/C-1, C-9. Moreover, the analysis of the ROESY spectrum as well as the coupling constant ($J$ = 15.6 Hz) between H-7 and H-8, indicating an *E*-geometry double bond. Therefore, the structure of **7** was determined and named as micranthumnin G.

The acetyl cholinesterase (AChE) inhibitory activities of all compounds were assayed using the Ellman method.[24] Compound **5** showed weak inhibitory activity (27.4%) at a concentration of 50 $\mu$M, using tacrine (0.33 $\mu$M) as the positive control (50.56% inhibition). The remaining compounds were inactive at 50 $\mu$M.

## Experimental Section

**General Experimental Procedures.** Optical rotations were measured on a JASCO P-1020 polarimeter. IR and UV spectra were recorded on a Bruker Tensor 27 FT-IR spectrometer and a Shimadzu UV2401PC spectrometer, respectively. 1D and 2D NMR spectra were recorded on Bruker Avance III-600, DRX-500 or AM-400 MHz spectrometers with TMS as internal standard at room temperature. HRESIMS were recorded on a

**Table 3.** $^{13}$**C NMR data of compounds 1–7 (**$\delta$ **in ppm)**

| Pos. | **1**[b] | **2**[b] | **3**[b] | **4**[a] | **5**[a] | **6**[a] | **7**[b] |
|---|---|---|---|---|---|---|---|
| 1 | 133.2 s | 133.9 s | 131.0 s | 136.9 s | 136.4 s | 131.5 s | 131.3 s |
| 2 | 109.9 d | 109.9 d | 110.0 d | 112.4 d | 112.1 d | 110.7 d | 108.7 d |
| 3 | 149.4 s | 149.4 s | 149.5 s | 151.0 s | 150.8 s | 151.3 s | 149.4 s |
| 4 | 147.6 s | 147.7 s | 148.0 s | 149.2 s | 149.3 s | 155.3 s | 147.0 s |
| 5 | 112.9 d | 112.9 d | 112.6 d | 115.0 d | 114.3 d | 113.5 d | 113.2 d |
| 6 | 118.9 d | 119.1 d | 120.3 d | 120.9 d | 120.9 d | 127.7 d | 118.5 d |
| 7 | 77.4 d | 79.2 d | 89.2 d | 80.1 d | 80.2 d | 192.9 d | 130.5 d |
| 8 | 71.3 d | 72.1 d | 71.3 d | 73.0 d | 73.0 d | | 123.8 d |
| 9 | 17.5 q | 18.7 q | 18.0 q | 19.4 q | 19.4 q | | 18.3 q |
| 1′ | 65.6 t | 65.6 t | 65.6 t | 68.4 t | 69.1 t | 66.6 t | 65.6 t |
| 2′ | 124.5 d | 124.5 d | 124.5 d | 41.6 t | 34.9 t | 125.1 d | 124.7 d |
| 3′ | 134.7 s | 134.7 s | 134.8 s | 155.9 s | 157.1 s | 137.0 s | 134.6 s |
| 4′ | 55.0 t | 54.9 t | 55.0 t | 128.4 d | 128.6 d | 55.6 t | 55.1 t |
| 5′ | 198.2 s | 198.3 s | 198.2 s | 193.8 s | 193.0 s | 200.5 s | 198.3 s |
| 6′ | 122.7 d | 122.7 d | 122.7 d | 127.3 d | 127.1 d | 123.8 d | 122.7 d |
| 7′ | 156.5 s | 156.6 s | 156.4 s | 156.8 s | 156.9 s | 158.4 s | 156.5 s |
| 8′ | 27.7 q | 27.7 q | 27.7 q | 27.9 q | 27.9 q | 27.7 q | 27.7 q |
| 9′ | 20.7 q | 20.7 q | 20.7 q | 20.9 q | 20.9 q | 20.9 q | 20.7 q |
| 10′ | 16.9 q | 16.9 q | 16.9 q | 19.6 q | 26.9 q | 17.1 q | 16.9 q |
| $OCH_3$-3 | 55.8 q | 55.8 q | 55.9 q | 56.6 q | 56.5 q | 56.4 q | 55.7 q |
| $OCH_3$-7 | | | 56.5 q | | | | |

[a]Recorded in $CD_3OD$ at 150MHz; [b]Recorded in $CDCl_3$ at 100MHz.

API QSTAR Pulsar 1 spectrometer. Column chromatography (CC) was performed on silica gel (100–200 mesh, Qingdao Marine Chemical Ltd., Qingdao, China), Sephadex LH-20 (Amersham Biosciences, Sweden) and RP-18 gel (40 × 75 $\mu$m, Fuji Silysia Chemical Ltd., Japan). Analytical and semipreparative HPLC were performed on SHIMADZU LC-20AT system equipped with Extend-C18 column (4.6 × 150 mm) and YMC-Pack ODS-A column (10 × 150 mm).

**Plant Material.** Stem bark of *I. micranthum* were collected in Dongchuan of Yunnan province, China, in May 2011. The plant material was identified by Dr. Rong Li of Kunming Institute of Botany, Chinese Academy of Sciences. A sample was deposited in our laboratory. A voucher specimen of *I. micranthum* (Li Rong 560) is deposited in State Key Laboratory of Phytochemistry and Plant Resources in West China, Kunming Institute of Botany, Chinese Academy of Sciences.

**Extraction and Isolation.** The air-dried stem bark of *I. micranthum* (14 kg) was powdered and extracted with MeOH (3 × 25 L) at room temperature, and concentrated in vacuo to give a crude extract. The extract was successively fractionated with $CHCl_3$ and EtOAc. A portion of the $CHCl_3$ extract (310 g) was separated by silica gel column chromatography, using $CHCl_3$/MeOH (20:1 to 2:1) as a gradient solvent system to afford fractions I–VI.

Fraction II was isolated by silica gel eluting with a gradient of petroleum ether/EtOAc (40:1, 20:1, 10:1, 5:1, to 2:1) to afford four subfractions (A1–A4). Fraction A2 was chromatographed over Sephadex LH-20 with MeOH to yield **7** (31 mg). Fraction III was subjected to RP-18 column chromatography (MeOH/$H_2O$, 30:70 to 80:20) to afford three fractions. The first fraction was chromatographed over Sephadex LH-20 with MeOH and applied to silica gel CC (eluted with $CHCl_3/Me_2CO$, 40:1), and then purified by semipreparative HPLC to give **1** (15 mg, $t_R$ 21.5 min, MeCN/$H_2O$ 39:61), and **2** (40 mg, $t_R$ 25.0 min, MeCN/$H_2O$ 39:61), respectively. The second fraction was subjected to silica gel CC using $CHCl_3/Me_2CO$ (40:1), with final purification by semipreparative HPLC (MeCN/$H_2O$ 45:55), to give **4** (3.5 mg, $t_R$ 17.0 min) and **5** (42 mg, $t_R$ 23.0 min). Compound **3** (3.2 mg) was obtained from the last fraction after repeated silica gel CC and Sephadex LH-20 CC, followed by semipreparative HPLC ($t_R$ 38.0 min, MeOH/$H_2O$ 70:30). Fraction IV was subjected to repeated silica gel CC (petroleum ether/$Me_2CO$, 95:5 to 4:1), to afford seven subfractions (B1–B7). Fraction B4, eluted with MeOH over Sephadex LH-20, was further separated on semipreparative HPLC to yield **6** (4.5 mg, $t_R$ 18.5 min, $CH_3OH/H_2O$ 70:30).

**Micranthumnin A (1):** yellow gum; $[\alpha]_D^{21}$ − 7.9 (*c* 0.13, MeOH); UV (MeOH) $\lambda_{max}$ (log $\varepsilon$) 233 (2.31), 204 (2.55) nm; IR $\nu_{max}$ (KBr) 3443, 2965, 2900, 1673, 1602, 1516, 1424, 1262, 1228, 1137, 994, 854, 621 cm$^{-1}$; $^1$H (600 MHz, $CD_3OD$) and $^{13}$C NMR (100 MHz, $CDCl_3$) data, see Tables 1 and 3; HREIMS: *m*/*z* 348.1931 $[M]^+$, (clacd for $C_{20}H_{28}O_5$, 348.1937).

**Micranthumnin B (2):** yellow gum; $[\alpha]_D^{21}$ − 7.6 (*c* 0.17, MeOH); UV (MeOH) $\lambda_{max}$ (log $\varepsilon$) 233 (2.27), 204 (2.52) nm; IR $\nu_{max}$ (KBr) 3424, 2973, 2933, 2912, 1685, 1617, 1513, 1448, 1262, 1225, 1138, 1035, 809, 624 cm$^{-1}$; $^1$H (600 MHz, $CD_3OD$) and $^{13}$C NMR (100 MHz, $CDCl_3$) data, see Tables 1 and 3; HREIMS: *m*/*z* 348.1925 $[M]^+$, (clacd for $C_{20}H_{28}O_5$, 348.1937).

**Micranthumnin C (3):** yellow gum; $[\alpha]_D^{21}$ – 20.2 (*c* 0.08, MeOH); UV (MeOH) $\lambda_{max}$ (log $\varepsilon$) 233 (2.21), 204 (2.47) nm; IR $\nu_{max}$ (KBr) 3441, 2968, 2932, 1687, 1620, 1513, 1449, 1261, 1138, 1034, 975 cm$^{-1}$; $^1$H (600 MHz, $CD_3OD$) and $^{13}$C NMR (100 MHz, $CDCl_3$) data, see Tables 2 and 3; HREIMS: *m*/*z* 362.2098 $[M]^+$, (clacd for $C_{21}H_{30}O_5$, 362.2093).

**Micranthumnin D (4):** yellow gum; $[\alpha]_D^{24}$ + 7.6 (*c* 0.10, MeOH); UV (MeOH) $\lambda_{max}$ (log $\varepsilon$) 268 (2.23), 203 (2.40) nm; IR $\nu_{max}$ (KBr) 3431, 2970, 2931, 1668, 1627, 1514, 1263, 1138, 1034, 871, 624 cm$^{-1}$; $^1$H (600 MHz) and $^{13}$C NMR (150 MHz) data ($CD_3OD$), see Tables 2 and 3; HREIMS: *m*/*z* 348.1937 $[M]^+$, (clacd for $C_{20}H_{28}O_5$, 348.1937).

**Micranthumnin E (5):** yellow gum; $[\alpha]_D^{24}$ – 13.6 (*c* 0.09, MeOH); UV (MeOH) $\lambda_{max}$ (log $\varepsilon$) 268 (2.28), 203 (2.46) nm; IR $\nu_{max}$ (KBr) 3425, 2971, 2933, 1669, 1626, 1515, 1263, 1138, 1033, 872, 769 cm$^{-1}$; $^1$H (600 MHz) and $^{13}$C NMR (150 MHz) data ($CD_3OD$), see Tables 2 and 3; HREIMS: *m*/*z* 348.1932 $[M]^+$, (clacd for $C_{20}H_{28}O_5$, 348.1937).

**Micranthumnin F (6):** colorless gum; $[\alpha]_D^{21}$ – 7.4 (*c* 0.20, MeOH); UV (MeOH) $\lambda_{max}$ (log $\varepsilon$) 230 (2.37), 205 (2.32) nm; IR $\nu_{max}$ (KBr) 3426, 2936, 1684, 1586, 1508, 1424, 1267, 1136, 991, 731 cm$^{-1}$; $^1$H (600 MHz, $CD_3OD$) and $^{13}$C NMR (150 MHz, $CDCl_3$) data, see Tables 2 and 3; HREIMS: *m*/*z* 302.1512 $[M]^+$, (clacd for $C_{18}H_{22}O_4$, 302.1518).

**Micranthumnin G (7):** colorless gum; $[\alpha]_D^{21}$ – 3.9 (*c* 0.31, MeOH); UV (MeOH) $\lambda_{max}$ (log $\varepsilon$) 253 (2.32), 210 (2.47) nm; IR $\nu_{max}$ (KBr) 2933, 2913, 1686, 1618, 1511, 1446, 1262, 1225, 1137, 964 cm$^{-1}$; $^1$H (400 MHz) and $^{13}$C NMR (100 MHz) data ($CDCl_3$), see Tables 2 and 3; HREIMS: *m*/*z* 314.1887 $[M]^+$, (clacd for $C_{20}H_{26}O_3$, 314.1882).

**Derivative Reaction. 1** [9 mg, 0.026 mmol in DMP (1 mL)] and **2** [10 mg, 0.029 mmol in DMP (1 mL)] were stirred with PPTs (1 mg), respectively, and protected with argon at room temperature for 24 hours.

**Anti-AChE Assay.** AChE inhibitory activities of the compounds isolated were assayed by the spectrophotometric method developed by Ellman et al.[11] Acetylthiocholine iodide (Sigma) was used as substrate in the assay. Compounds were dissolved in DMSO. The mixture contained 110 $\mu$L phosphate buffer (pH 8.0), 10 $\mu$L of test compound solution (50 $\mu$M), and 40 $\mu$L AChE solution (0.04 U/100 $\mu$L), and the mixture was incubated for 20 min (30 °C). The reaction was initiated by the addition of 20 $\mu$L of DTNB (6.25 mM) and 20 $\mu$L of acetylthiocholine iodide (6.25 mM). The hydrolysis of acetylthiocholine was monitored at 405 nm after 30 min. Tacrine was used as positive control. All the reactions were performed in triplicate. The percentage inhibition was calculated as follows: % inhibition = $(E – S)/E \times 100$ (*E* is the activity of the enzyme without test compound and *S* is the activity of enzyme with test compound).

## Electronic Supplementary Material

Supplementary material is available in the online version of this article at http://dx.doi.org/ 10.1007/s13659-013-0007-x and is accessible for authorized users.

## Acknowledgments

This work was supported by the National Natural Science Foundation (20872148). The authors are grateful to the analytical group of the Laboratory of Phytochemistry, Kunming Institute of Botany, Chinese Academy of Sciences for measuring NMR, MS, and IR data.

**Open Access** This article is distributed under the terms of the Creative Commons Attribution License which permits any use, distribution, and reproduction in any medium, provided the original author(s) and source are credited.

## Electronic supplementary material

Supplementary material, approximately 4.23 MB.

## References

[1] Wu C (1997). Flora of China.

[2] Wu C (2003). The Families and Genera of Angiospers in China.

[3] Wu C (2000). Flora Yunnannica.

[4] Ngo K S, Brown G D (1996). Tetrahedron.

[5] Dong X J, Zhu X D, Wang Y F, Wang Q, Ju P, Luo S D (2006). Helv. Chim. Acta..

[6] Huang J M, Yang C S, Zhao R, Takahashi H, Fukuyama Y (2004). Chem. Pharm. Bull..

[7] Yokoyama R, Huang J M, Hosoda A, Kino K, Yang C S, Fukuyama Y (2003). J. Nat. Prod..

[8] Schmidt T J, Schmidt H M, Müller E, Peters W, Fronczek F R, Truesdale A, Fischer N H (1998). J. Nat. Prod..

[9] Sy L K, Brown G D (1998). J. Nat. Prod..

[10] Takahashi K, Takani M (1975). Chem. Pharm. Bull..

[11] Dong C F, Liu L, Luo H R, Li X N, Guan Z Y, Wang Y F (2012). Nat. Prod. Bioprospect..

[12] Sy L K, Brown G D (1998). J. Nat. Prod..

[13] Morimoto S, Tanabe H, Nanaka G I, Nishioka I (1988). Phytochemistry.

[14] Park I K, Shin S C (2005). J. Agric. Food Chem..

[15] Itoigawa M, Ito C, Tokuda H, Enjo F, Nishinoc H, Furukawa H (2004). Cancer Lett..

[16] Yokoyama R, Huang J M, Yang C S, Fukuyama Y (2002). J. Nat. Prod..

[17] Kubo M, Okada C, Huang J M, Harada K, Hioki H, Fukuyama Y (2009). Org. Lett..

[18] Trzoss L, Xu J, Lacoske M H, Mobley W C, Theodorakis E A (2011). Org. Lett..

[19] Zhang S Q, Chen Y, Li Q, Hu Z Y (2009). Chinese Traditional Patent Medicine.

[20] Li H L, Wang G L (1994). Nat. Prod. Res. Dev..

[21] Perry N B, Foster L M, Lorimer S D, May B C H, Weavers R T, Toyota M, Nakaishi E, Asakawa Y (1996). J. Nat. Prod..

[22] Shen C C, Ni C L, Shen Y C, Huang Y L, Kuo C H, Wu T S, Chen C C (2009). J. Nat. Prod..

[23] Pelter A, Peverall S, Pitchford A (1996). Tetrahedron.

[24] Ellman G L, Courtney K D, Andres V J, Featherstone R M (1961). Biochem. Pharmacol..

