# Supplementary material for: New monoterpene phenyl ethers from Illicium micranthum
Source: Nat Prod Bioprospect. 2013 Mar 16;3(2):43–7. doi: 10.1007/s13659-013-0007-x (PMC4131660; doi:10.1007/s13659-013-0007-x)
Supplement: Supplementary file 1 — Supplementary material, approximately 4.23 MB. [file 13659_2013_7_MOESM1_ESM.pdf]

Electronic Supplementary Material

New monoterpene phenyl ethers from *Illicium micranthum*

Zheng-Ye GUAN,[a,b] Chuan-Fu DONG,[a] Li GAO,[a,b] Jia-Ping WANG,[a,b] Shi-De LUO,[a] and Yi-Fen WANG[a,]*

[a]State Key Laboratory of Phytochemistry and Plant Resources in West China, Kunming Institute of Botany, Chinese Academy of Sciences, Kunming 650201, China

[b]University of Chinese Academy of Sciences, Beijing 100049, China

Received 24 January 2013; Accepted 10 March 2013

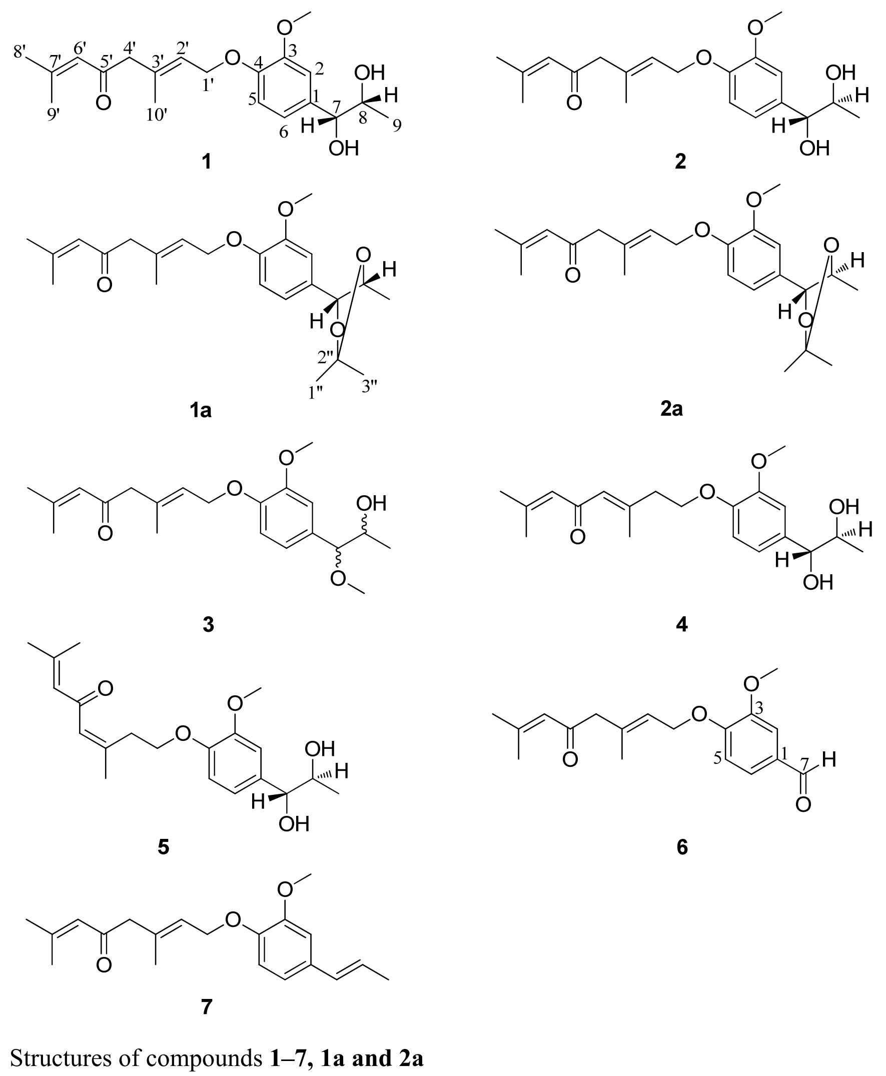

Structures of compounds **1–7, 1a and 2a**

*To whom correspondence should be addressed. E-mail: wangyifen@mail.kib.ac.cn

Figure 1S-7S. NMR and MS spectra of compound **1**

Figure 8S-14S. NMR and MS spectra of compound **2**

Figure 15S-17S. NMR spectra of compound **1a**

Figure 18S-20S. NMR spectra of compound **2a**

Figure 21S-27S. NMR and MS spectra of compound **3**

Figure 28S-34S. NMR and MS spectra of compound **4**

Figure 35S-41S. NMR and MS spectra of compound **5**

Figure 42S-48S. NMR and MS spectra of compound **6**

Figure 49S-55S. NMR and MS spectra of compound **7**

Figure 1S. ^{1}H NMR of compound **1**.

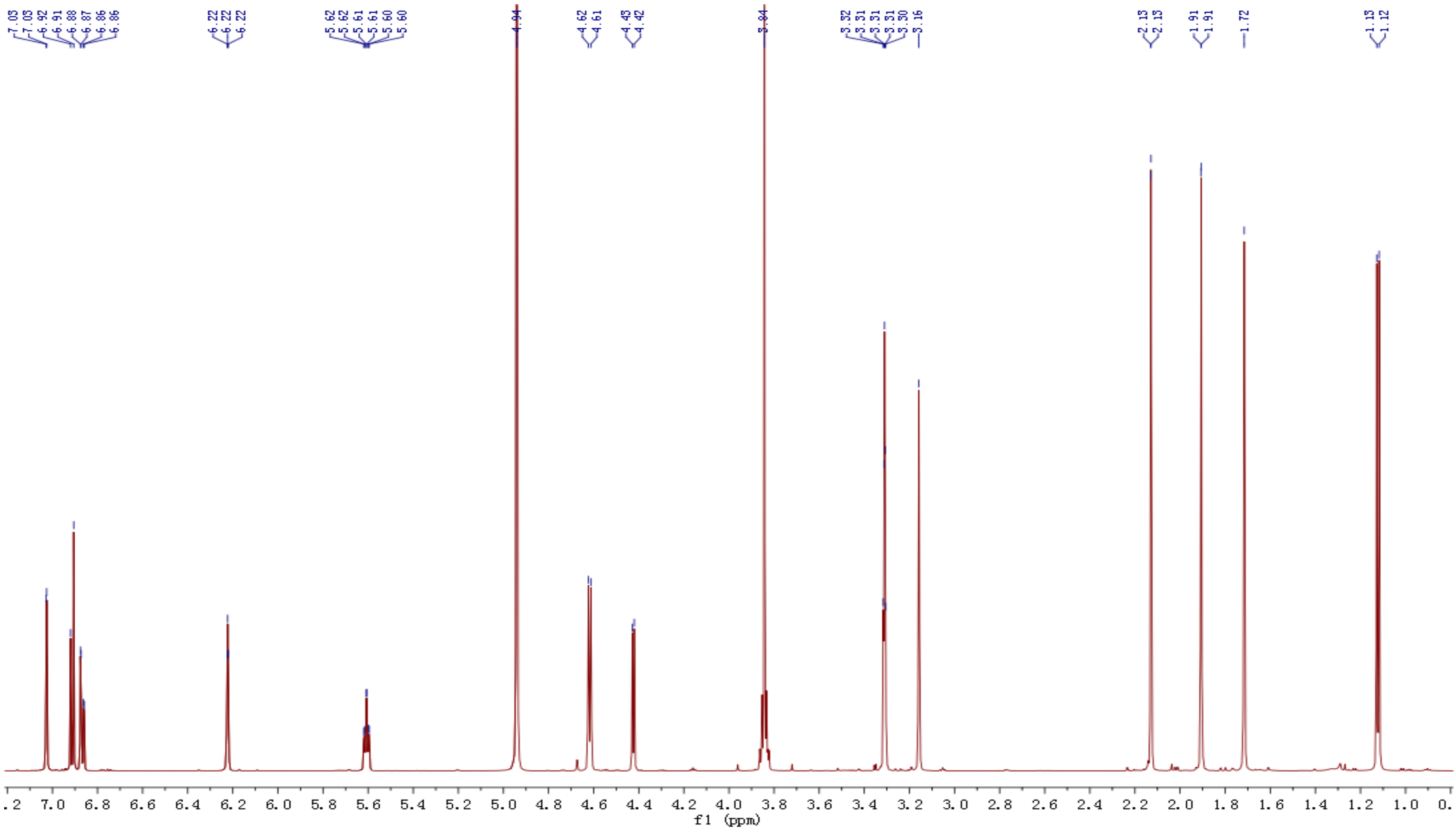

Figure 2S. ^{13}C NMR and DEPT of compound **1**.

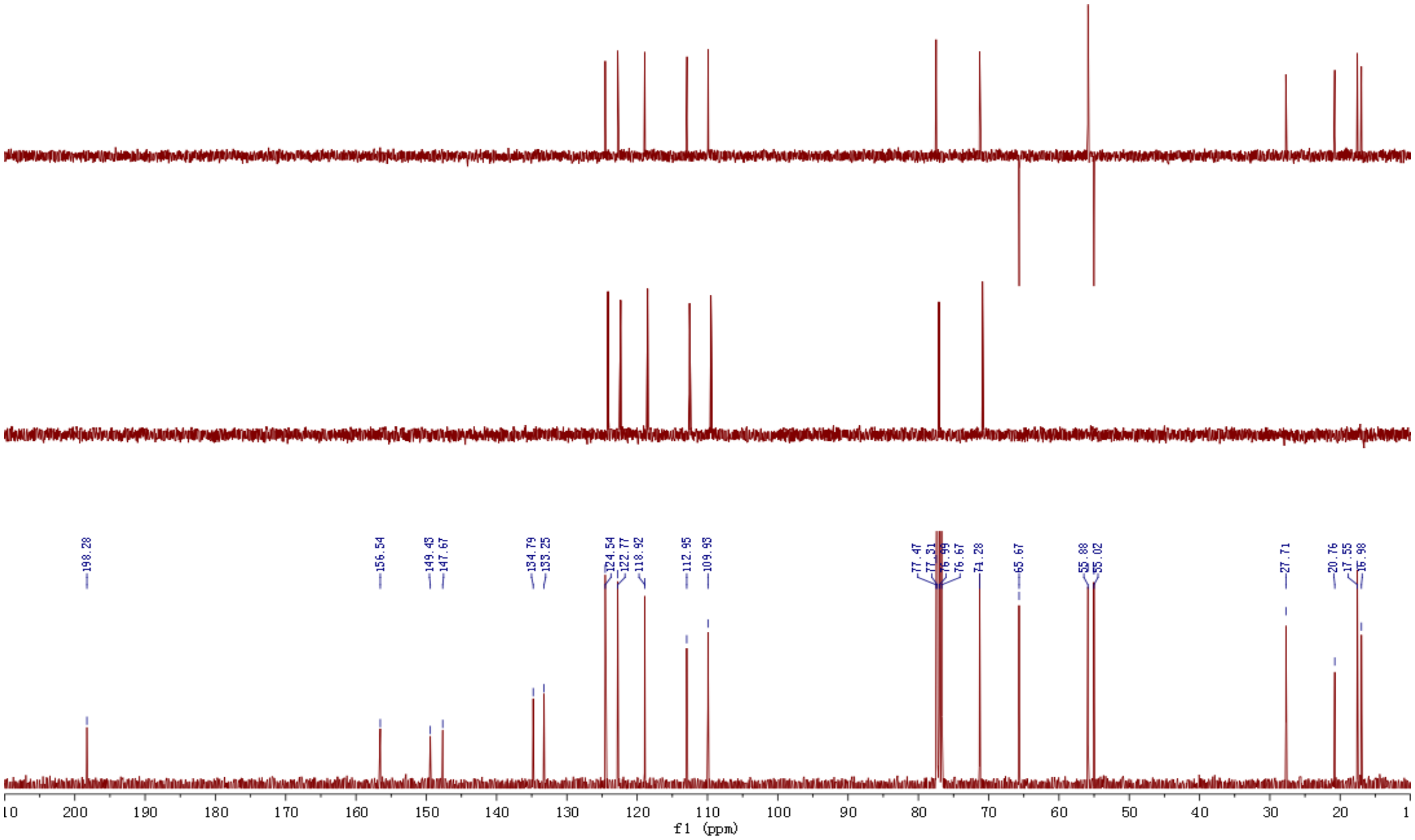

Figure 3S. HSQC of compound **1**

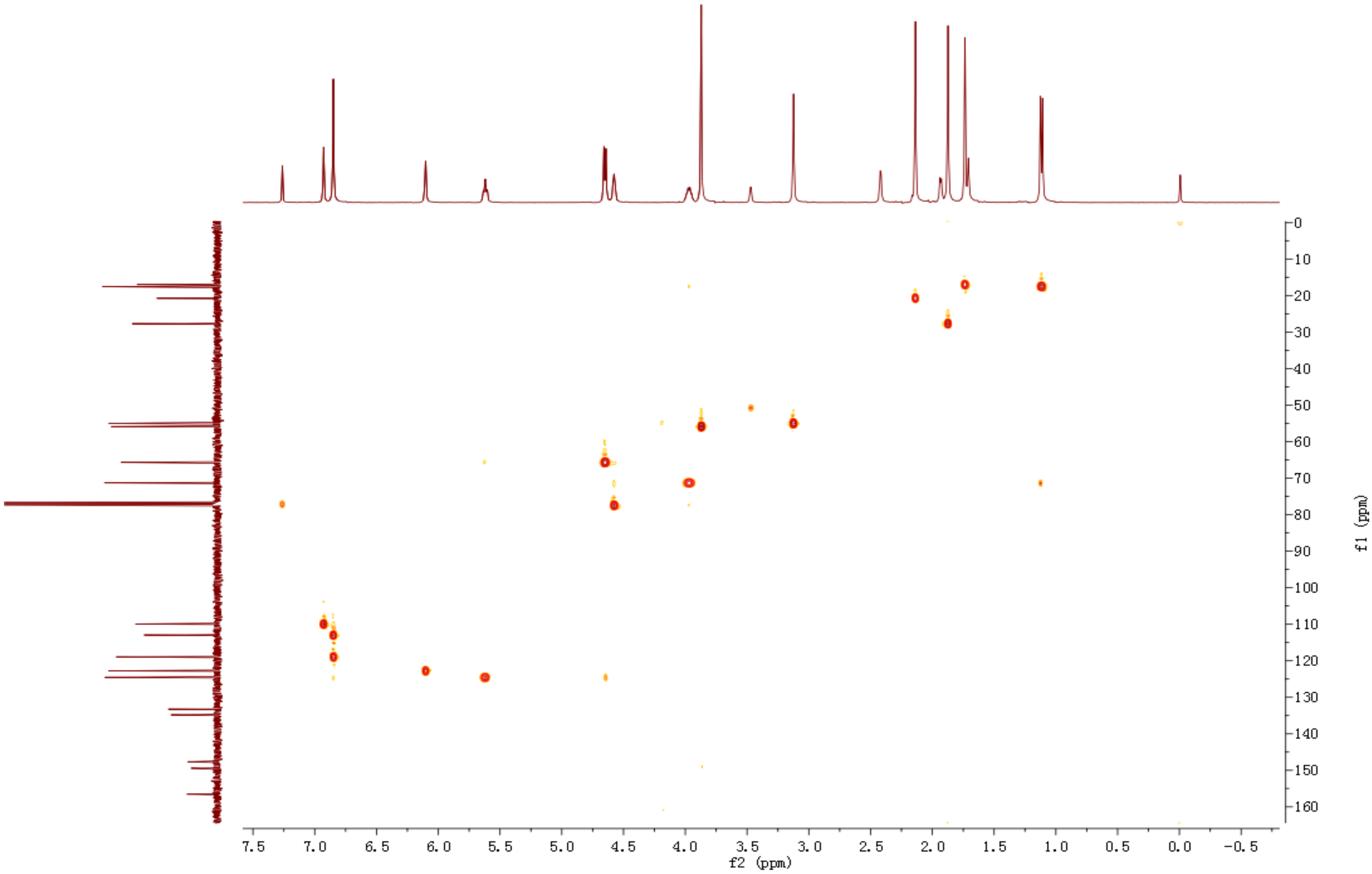

Figure 4S. HMBC of compound **1**.

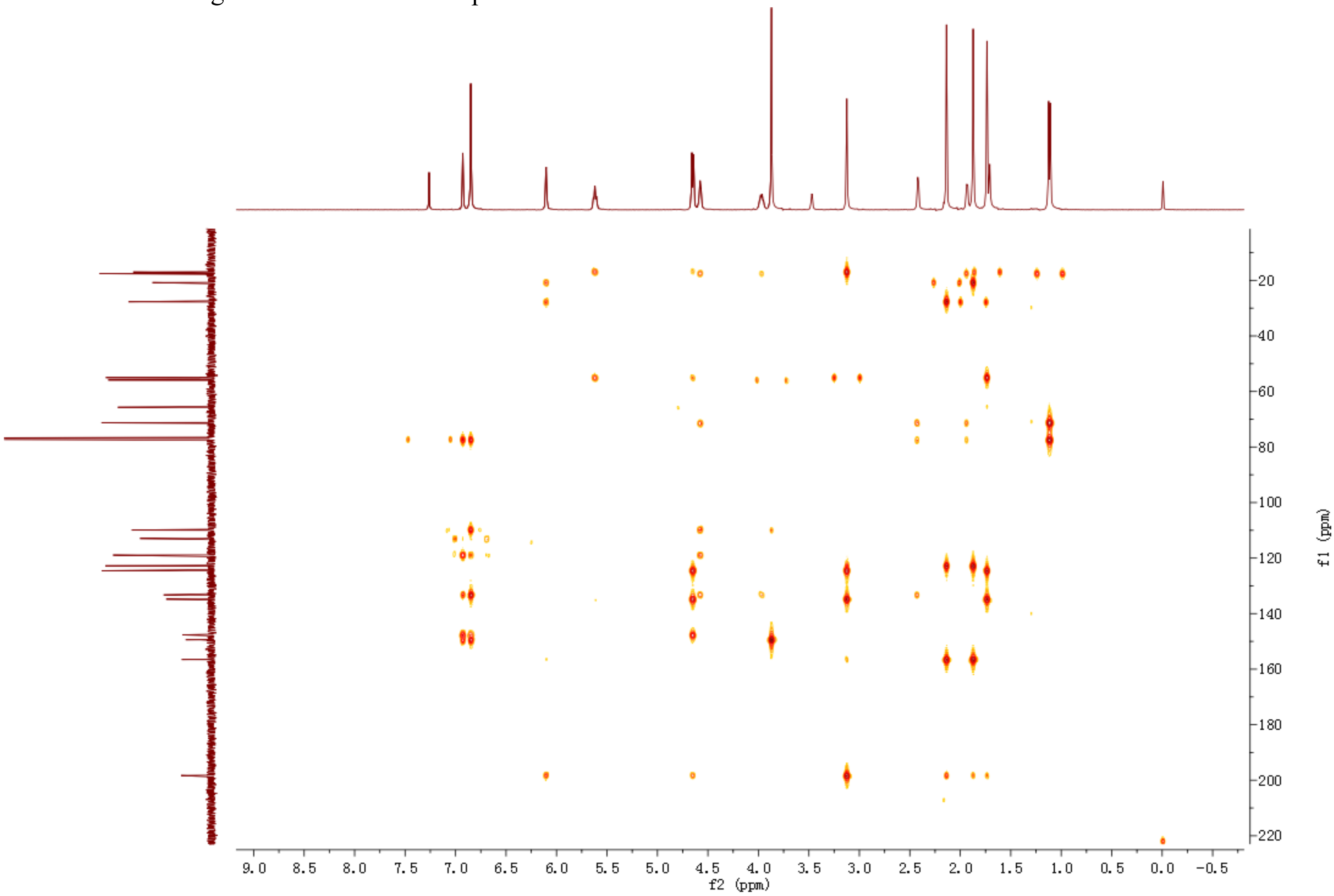

Figure 5S. H-H COSY of compound **1**.

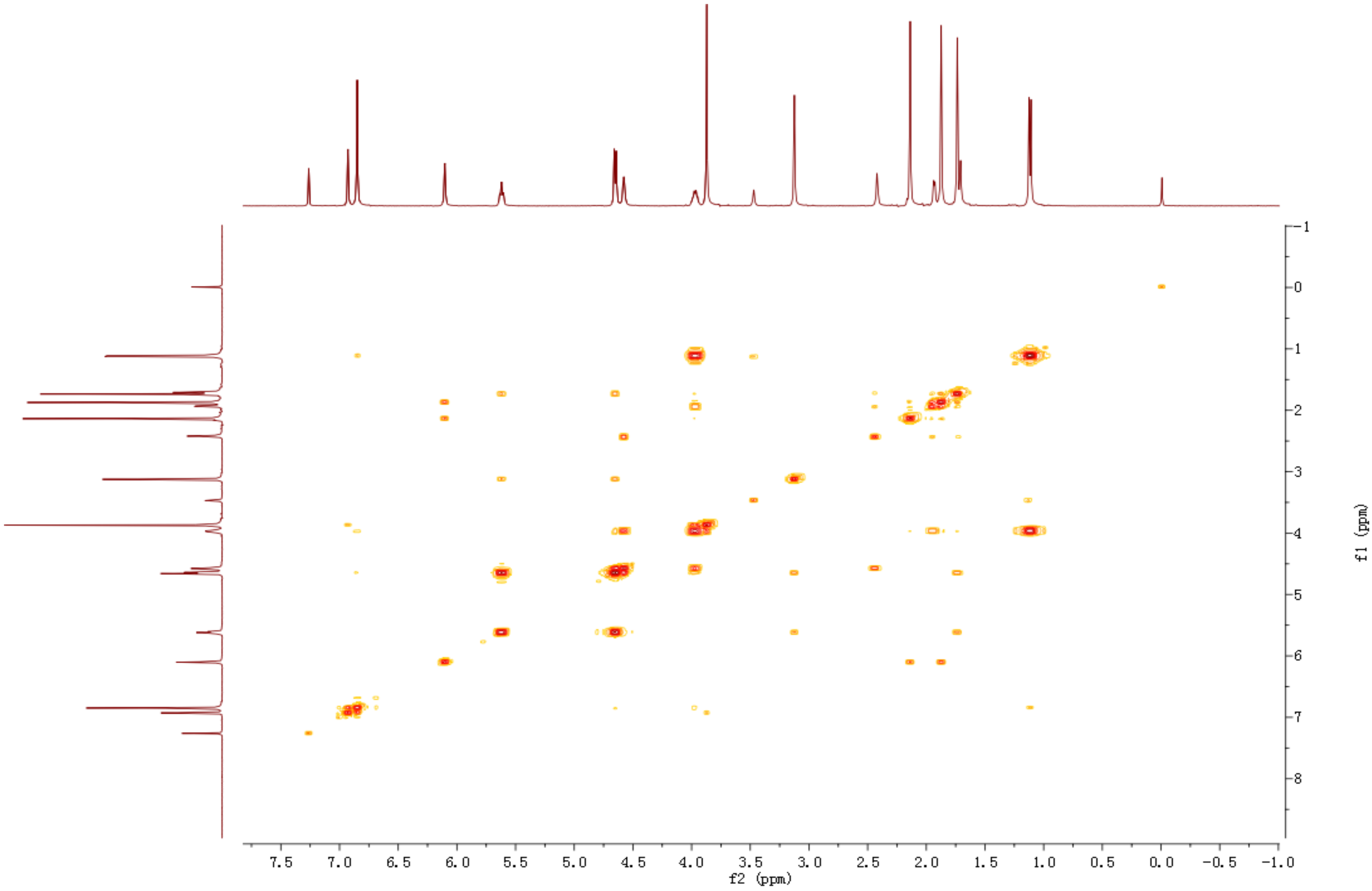

Figure 6S. ROESY of compound **1**.

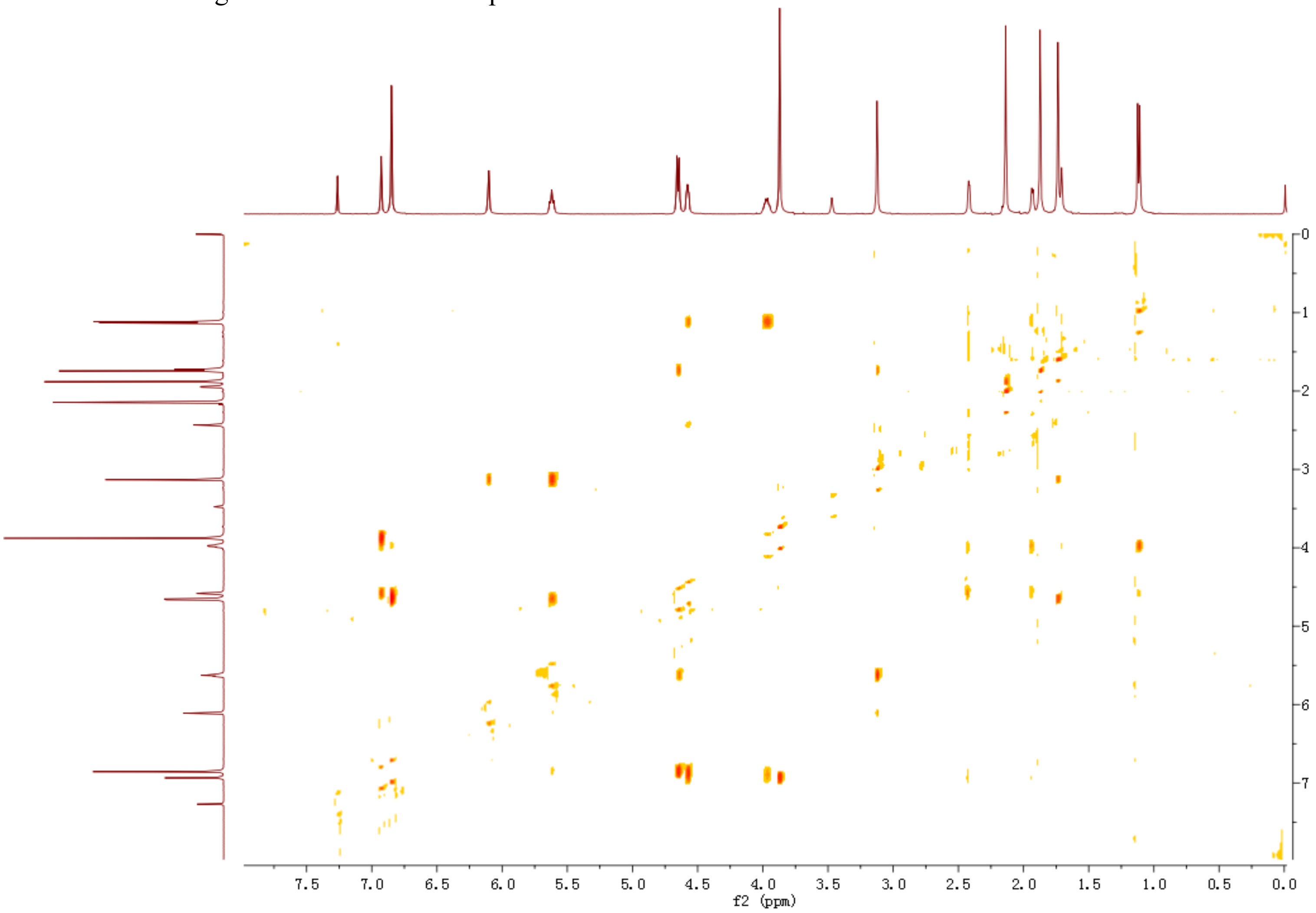

Figure 7S. HRESIMS of compound **1**.

Elemental Composition Report

Single Mass Analysis (displaying only valid results)

Tolerance = 10.0 PPM / DBE: min = 0.5, max = 60.0
Selected filters: None

Monoisotopic Mass, Odd and Even Electron Ions
17 formula(e) evaluated with 1 results within limits (up to 51 closest results for each mass)
Elements Used:
C: 0-200 H: 0-400 O: 3-6

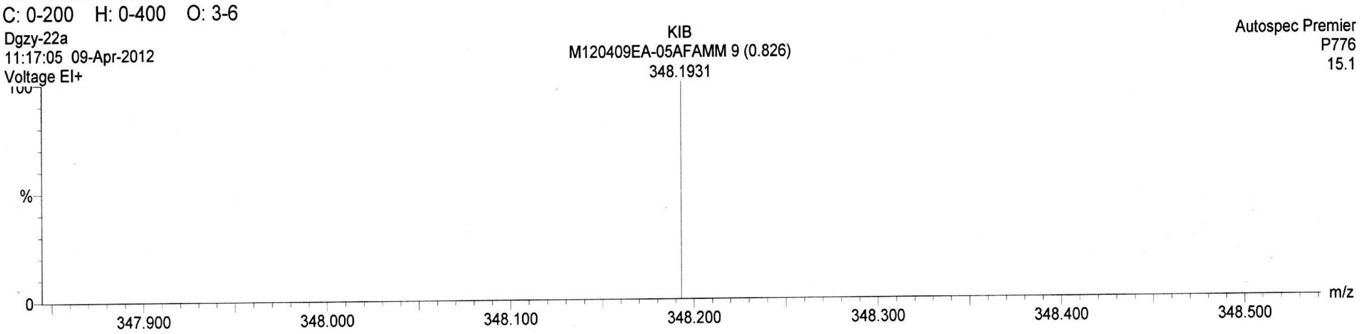

Minimum:				0.5		
Maximum:		73.0	10.0	60.0		
Mass	Calc. Mass	mDa	PPM	DBE	i-FIT	Formula
348.1931	348.1937	-0.6	-1.7	7.0	5546027.0	C20 H28 O5

Figure 8S. ^{1}H NMR of compound **2**.

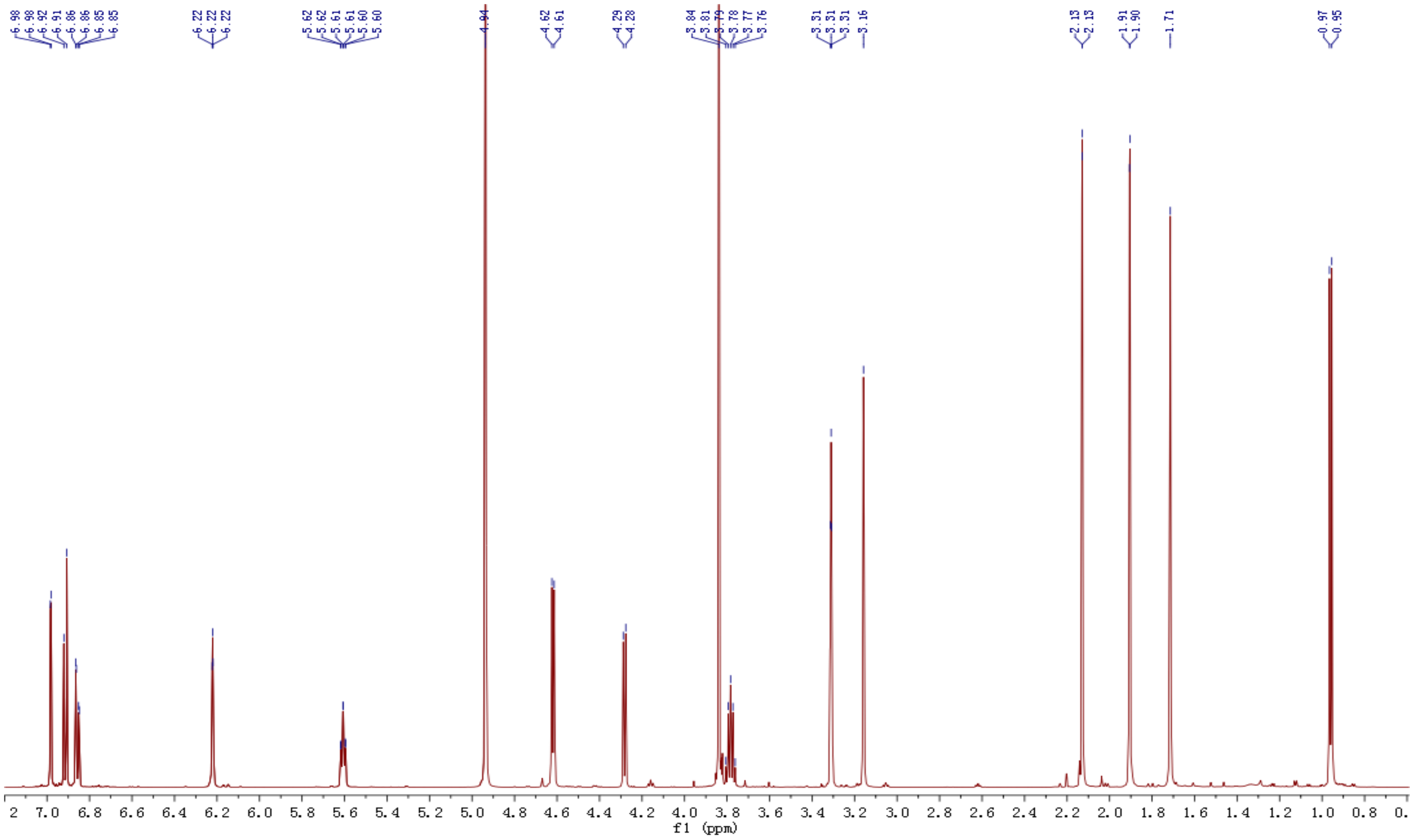

Figure 9S. ^{13}C NMR and DEPT of compound **2**.

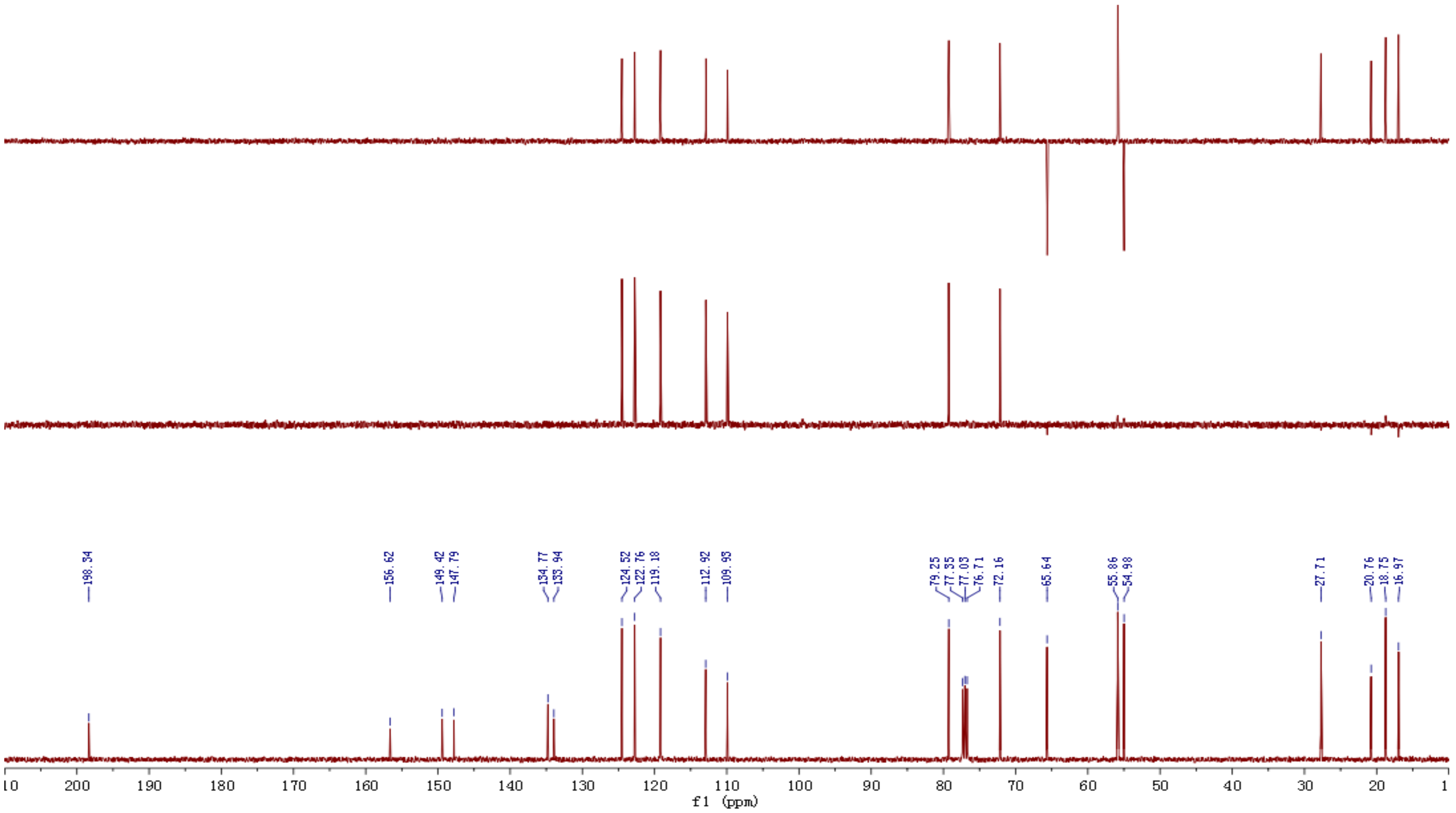

Figure 10S. HSQC of compound **2**.

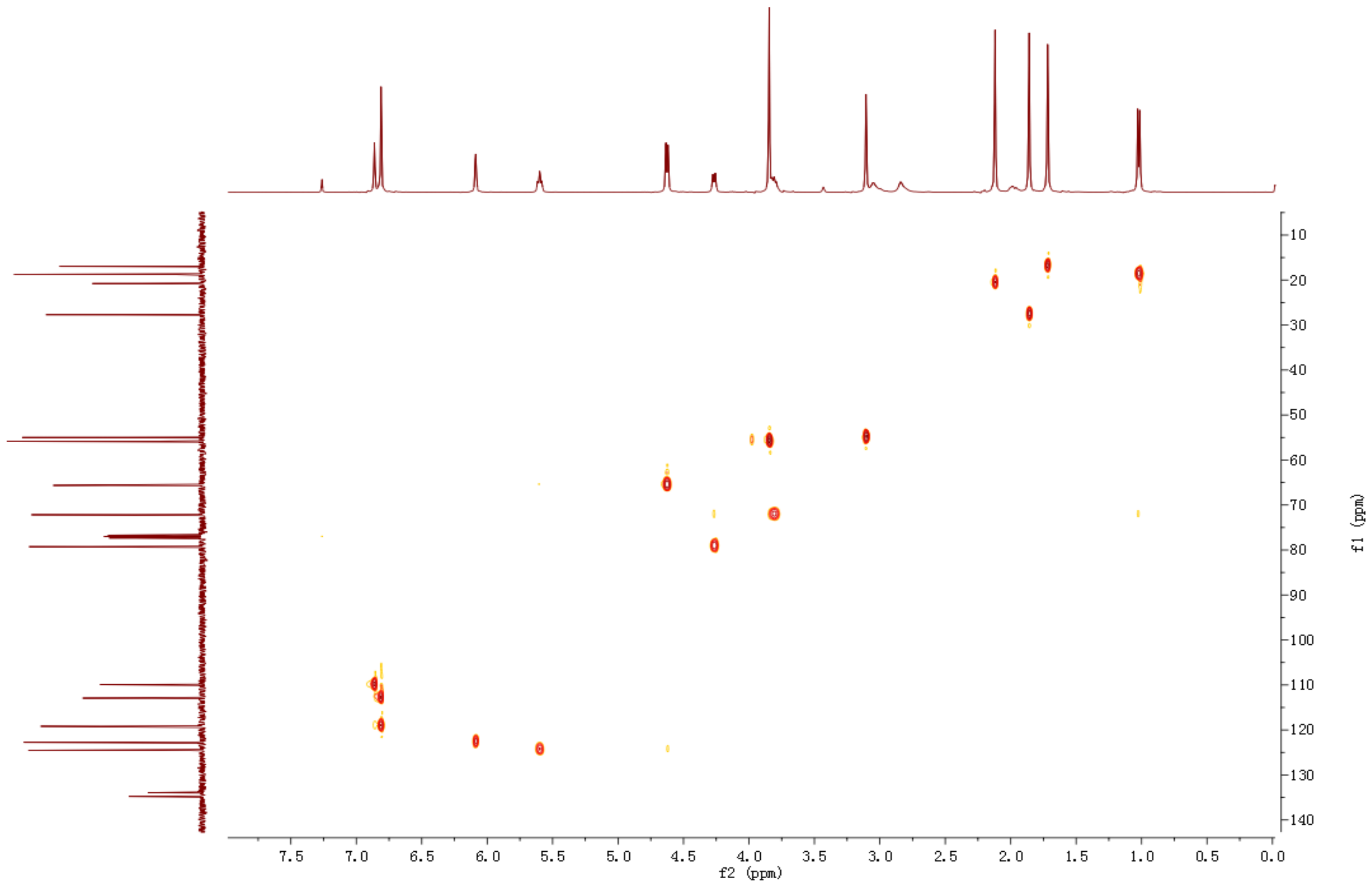

Figure 11S. HMBC of compound **2**.

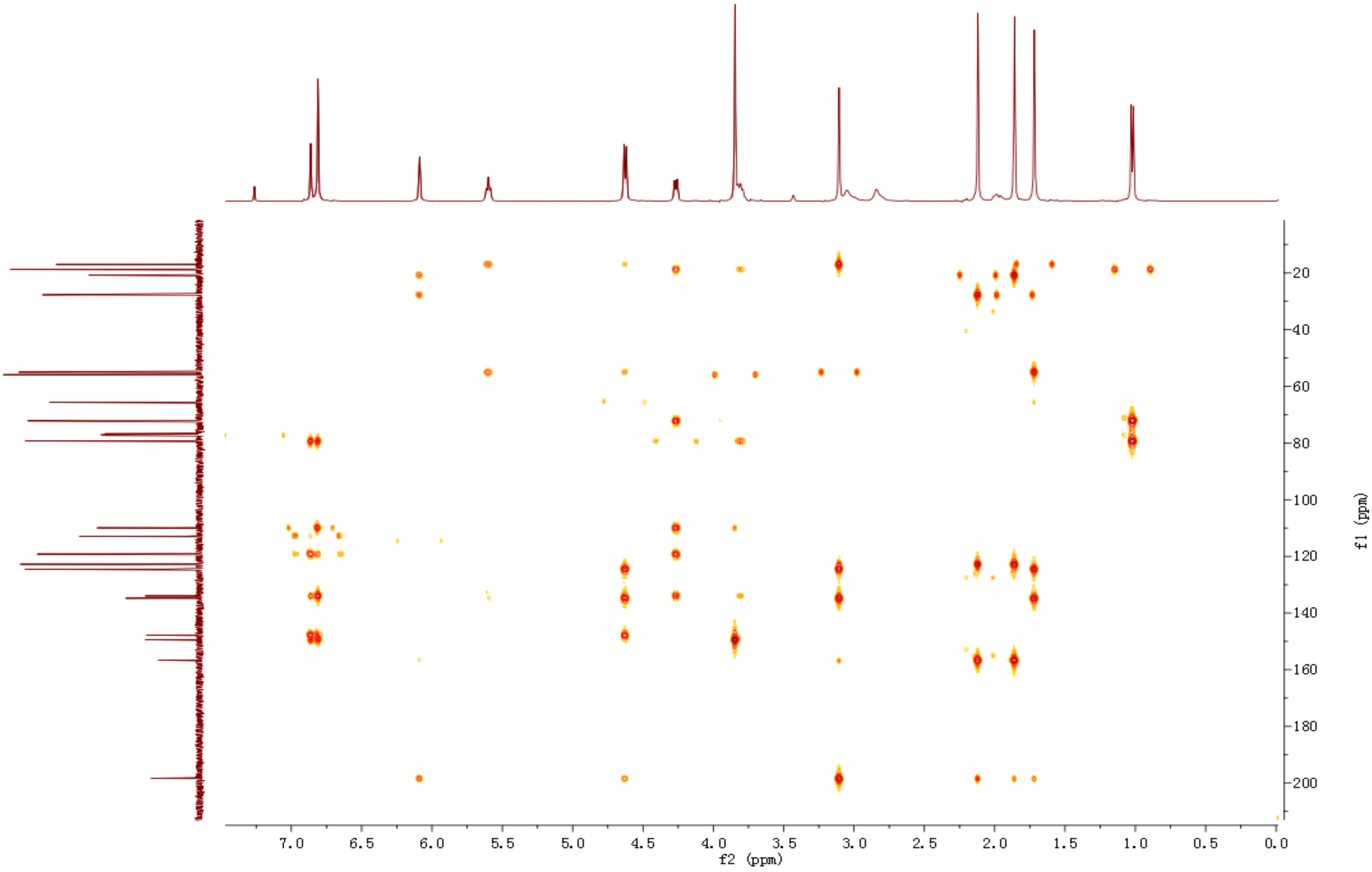

Figure 12S. ^1H-^1H COSY of compound **2**.

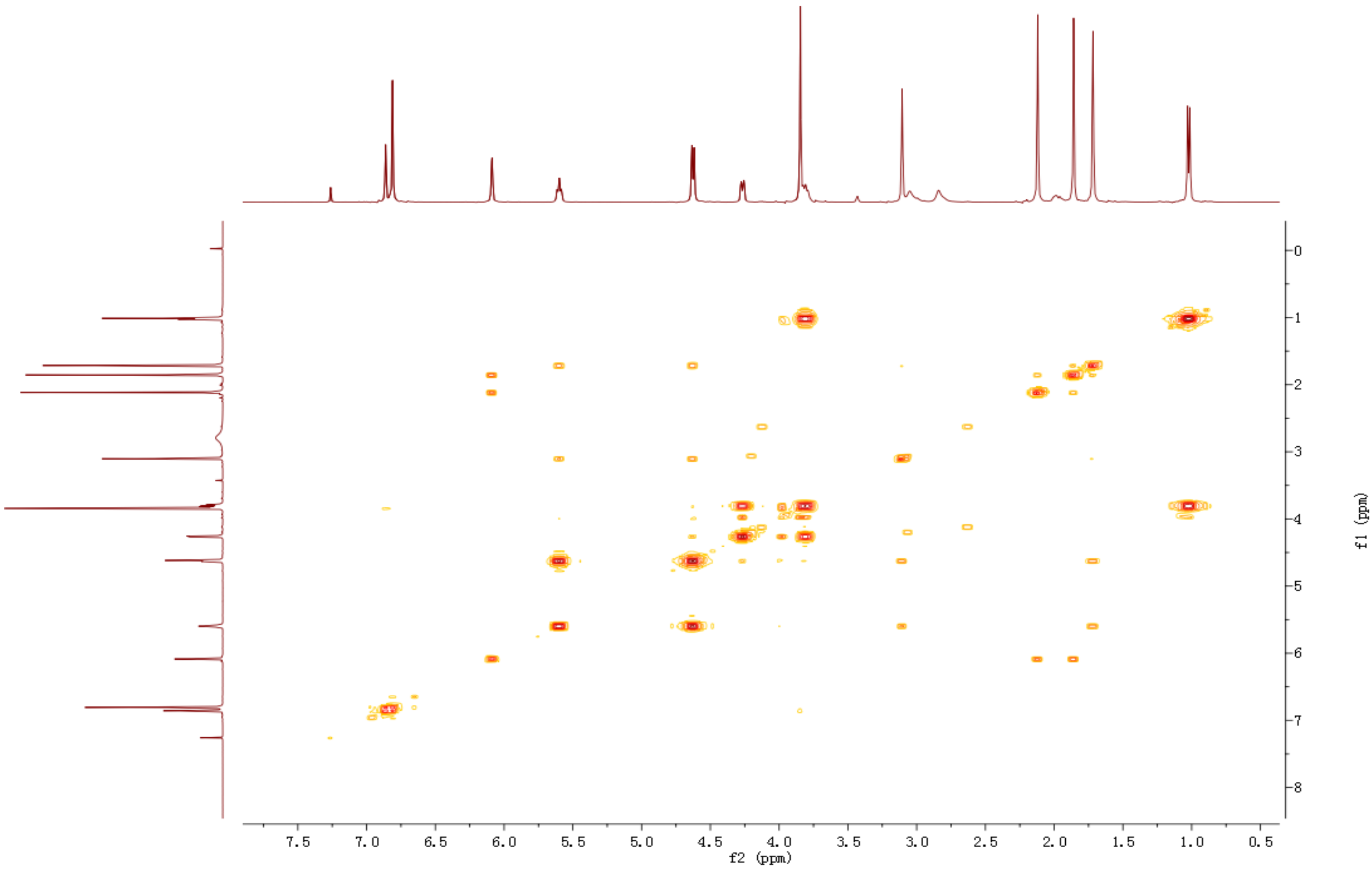

Figure 13S. ROESY of compound **2**.

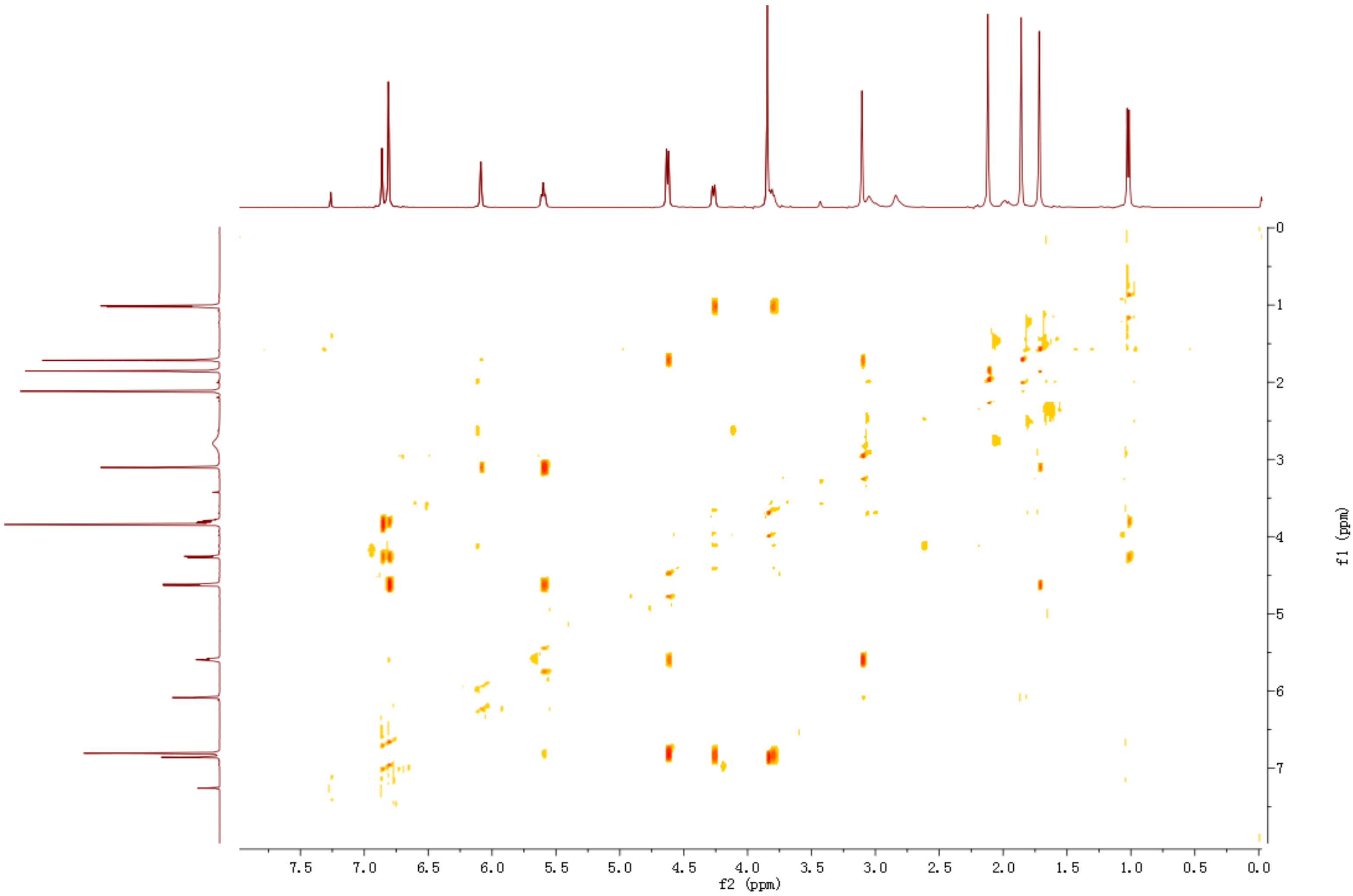

Figure14S. HRESIMS of compound **2**.

Elemental Composition Report **Page 1**

Single Mass Analysis (displaying only valid results)
Tolerance = 10.0 PPM / DBE: min = 0.5, max = 60.0
Selected filters: None

Monoisotopic Mass, Odd and Even Electron Ions
17 formula(e) evaluated with 1 results within limits (up to 51 closest results for each mass)
Elements Used:
C: 0-200 H: 0-400 O: 3-6

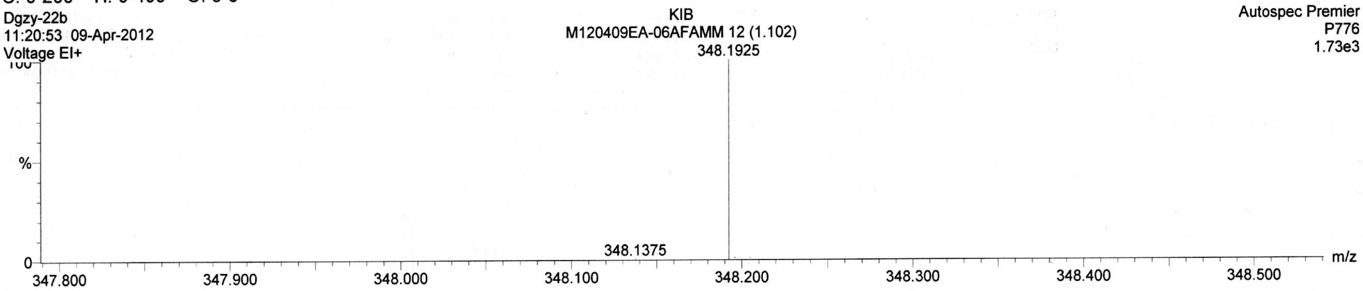

Minimum:				0.5		
Maximum:		73.0	10.0	60.0		
Mass	Calc. Mass	mDa	PPM	DBE	i-FIT	Formula
348.1925	348.1937	-1.2	-3.4	7.0	5546874.5	C20 H28 O5

Figure 15S. ^{1}H NMR of compound **1a**.

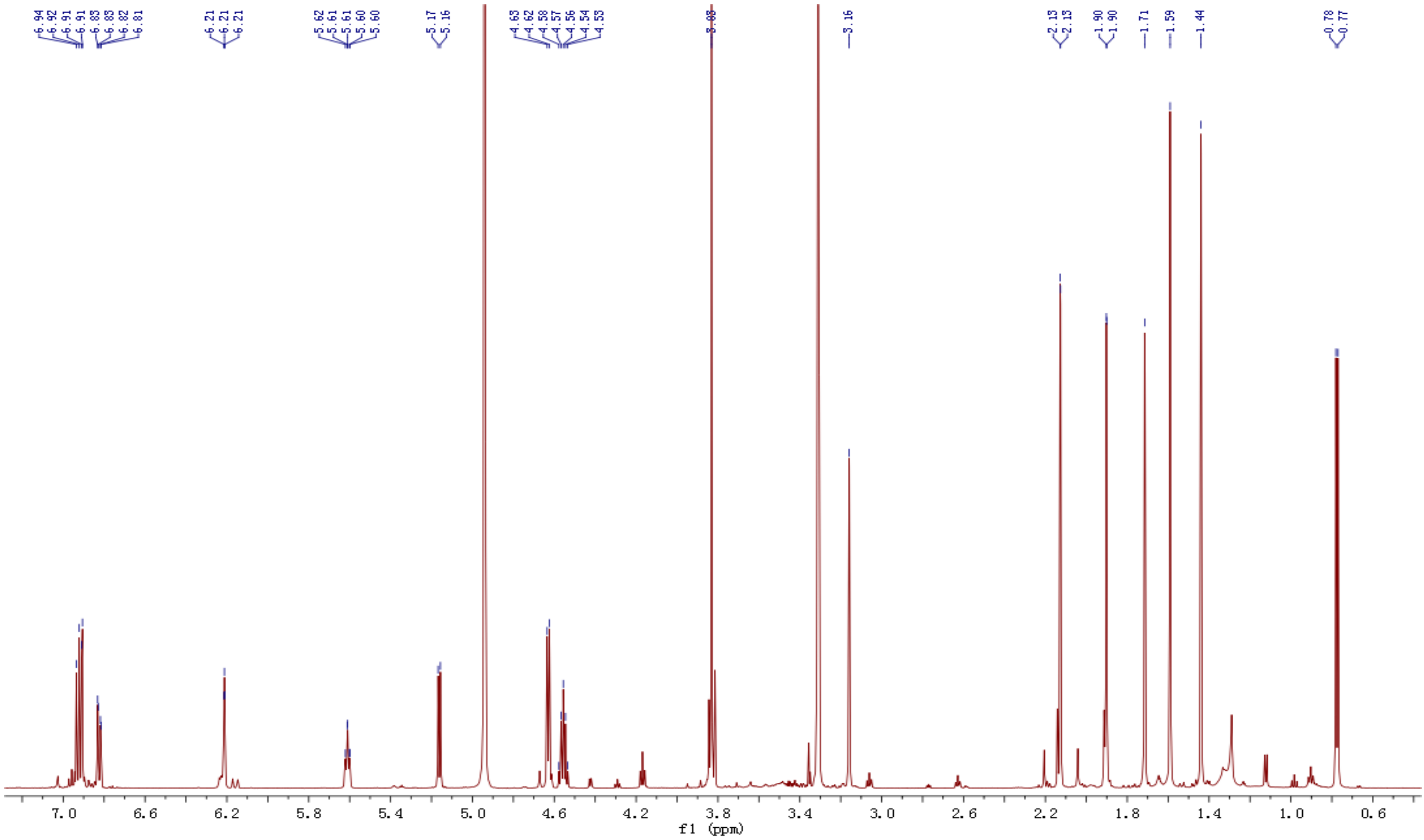

Figure 16S. ^{13}C NMR and DEPT of compound **1a**.

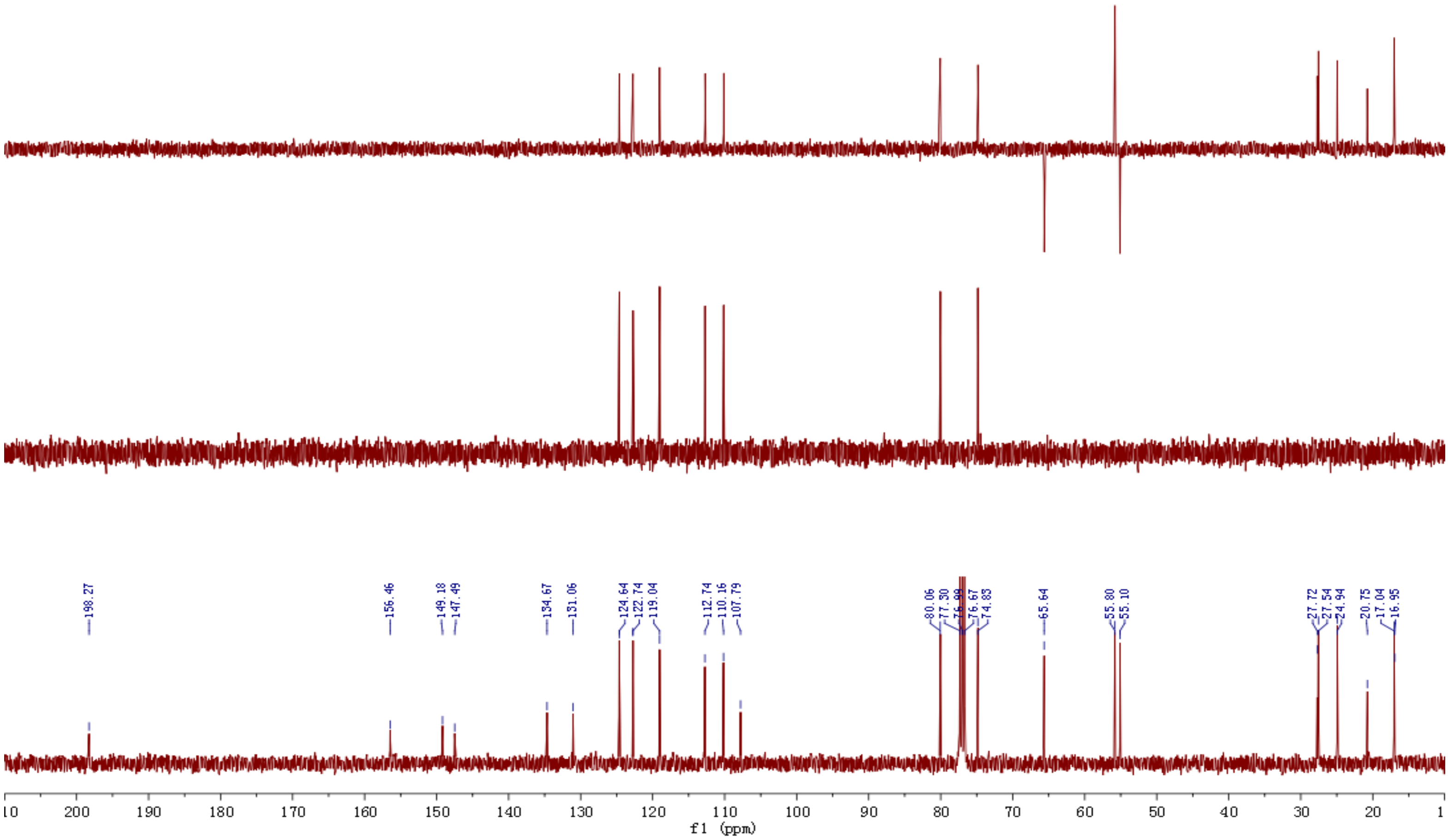

Figure 17S. ROESY of compound **1a**.

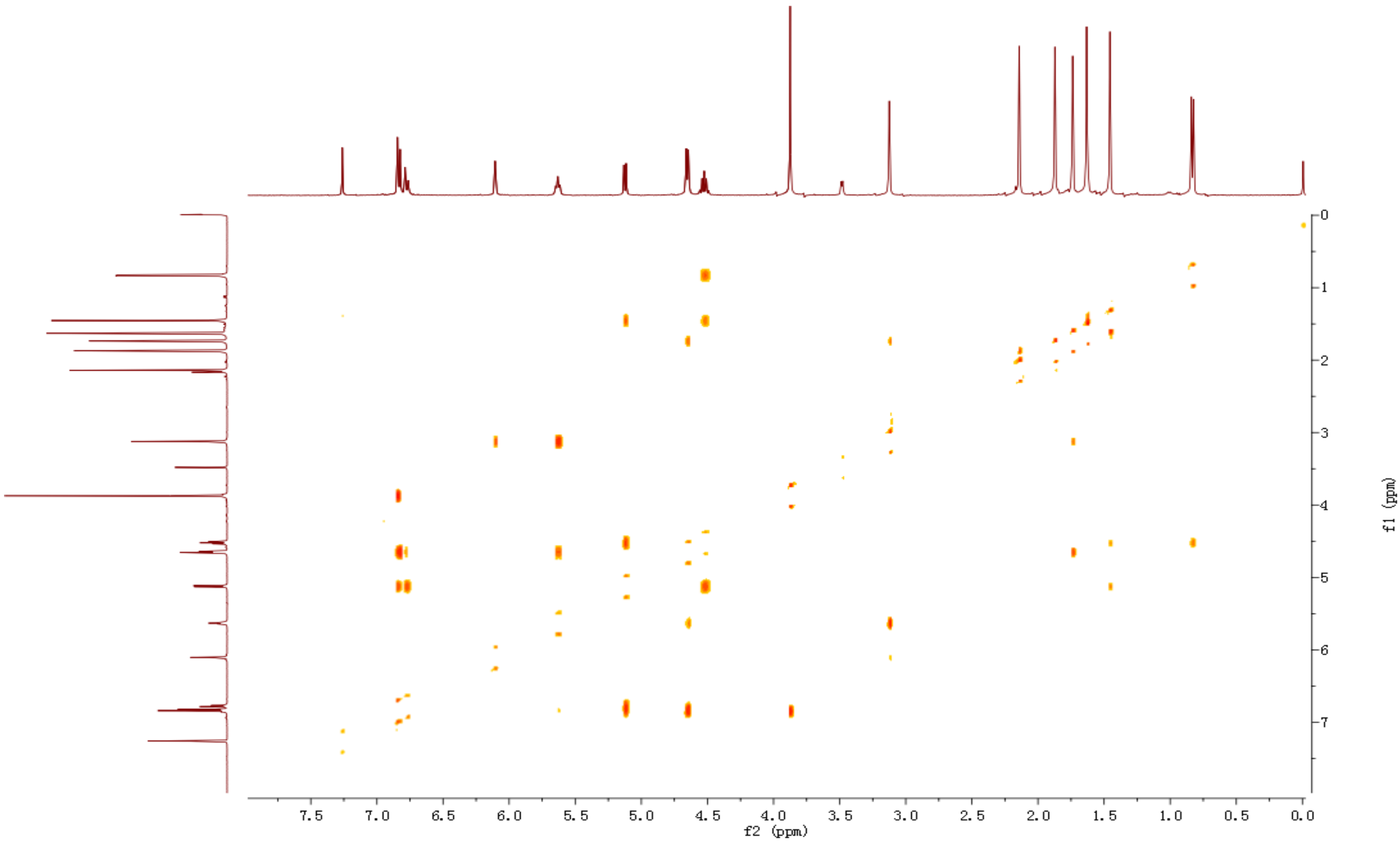

Figure 18S. ^{1}H NMR of compound **2a**.

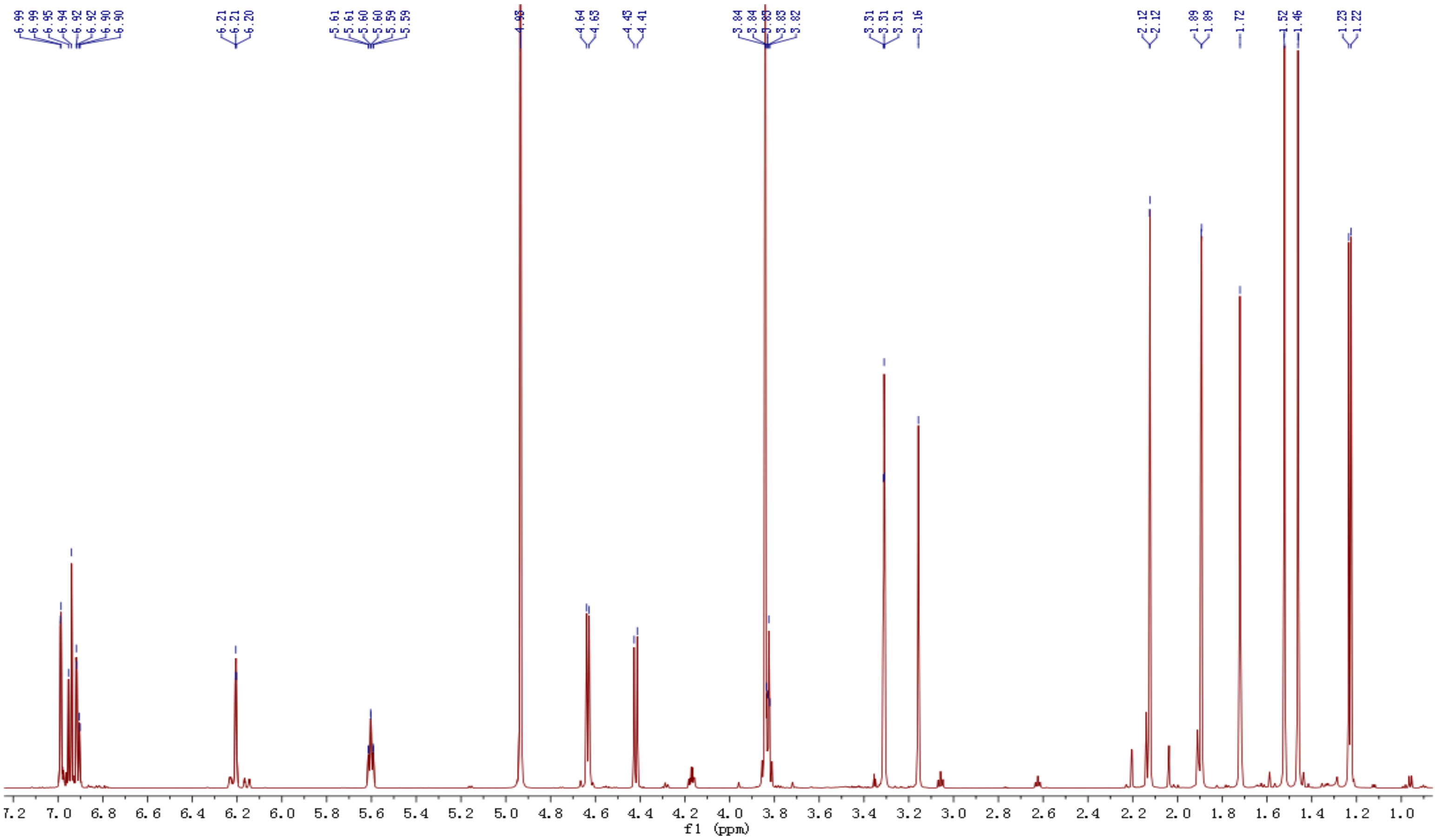

Figure 19S. ^{13}C NMR and DEPT of compound **2a**.

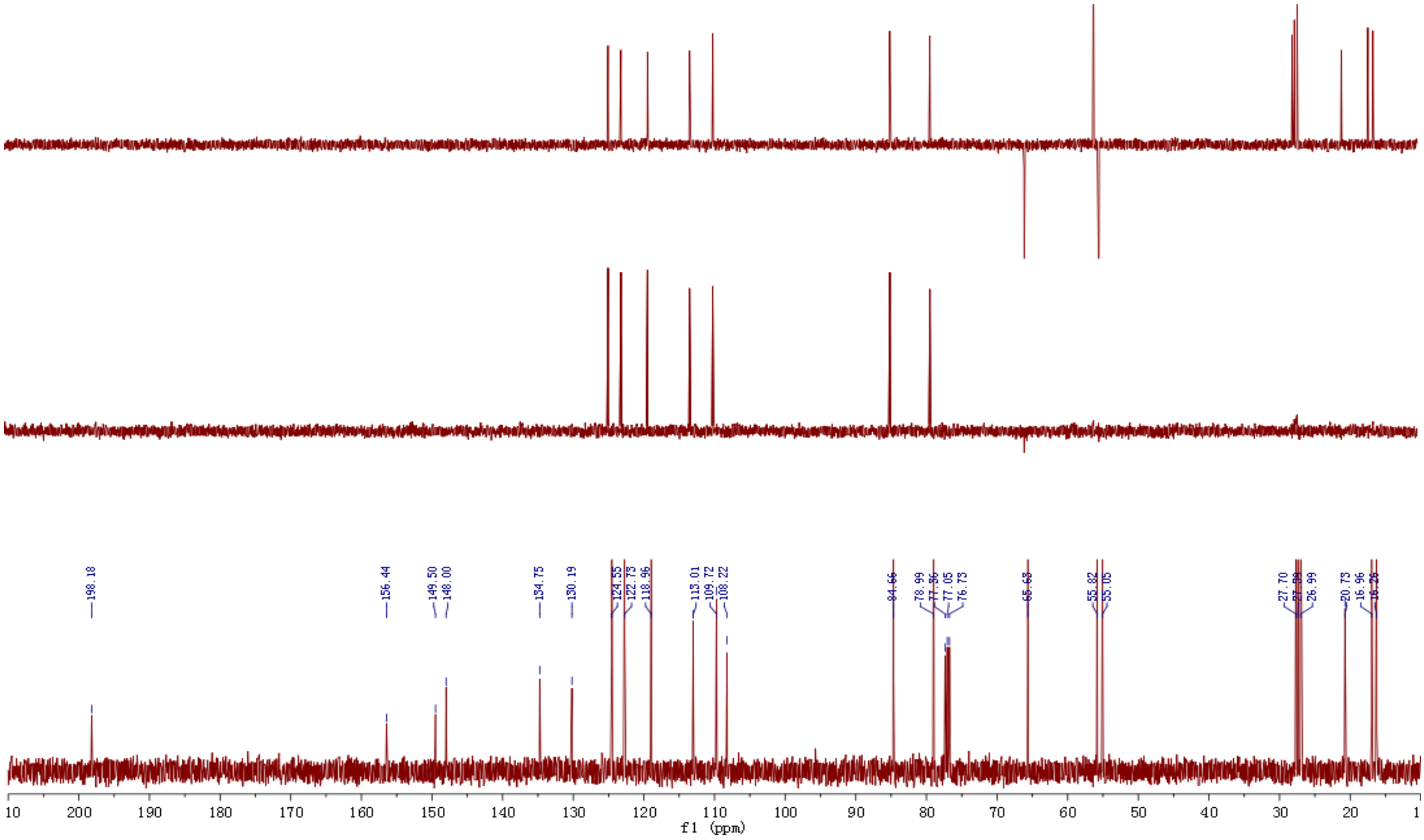

Figure 20S. ROESY of compound **2a**.

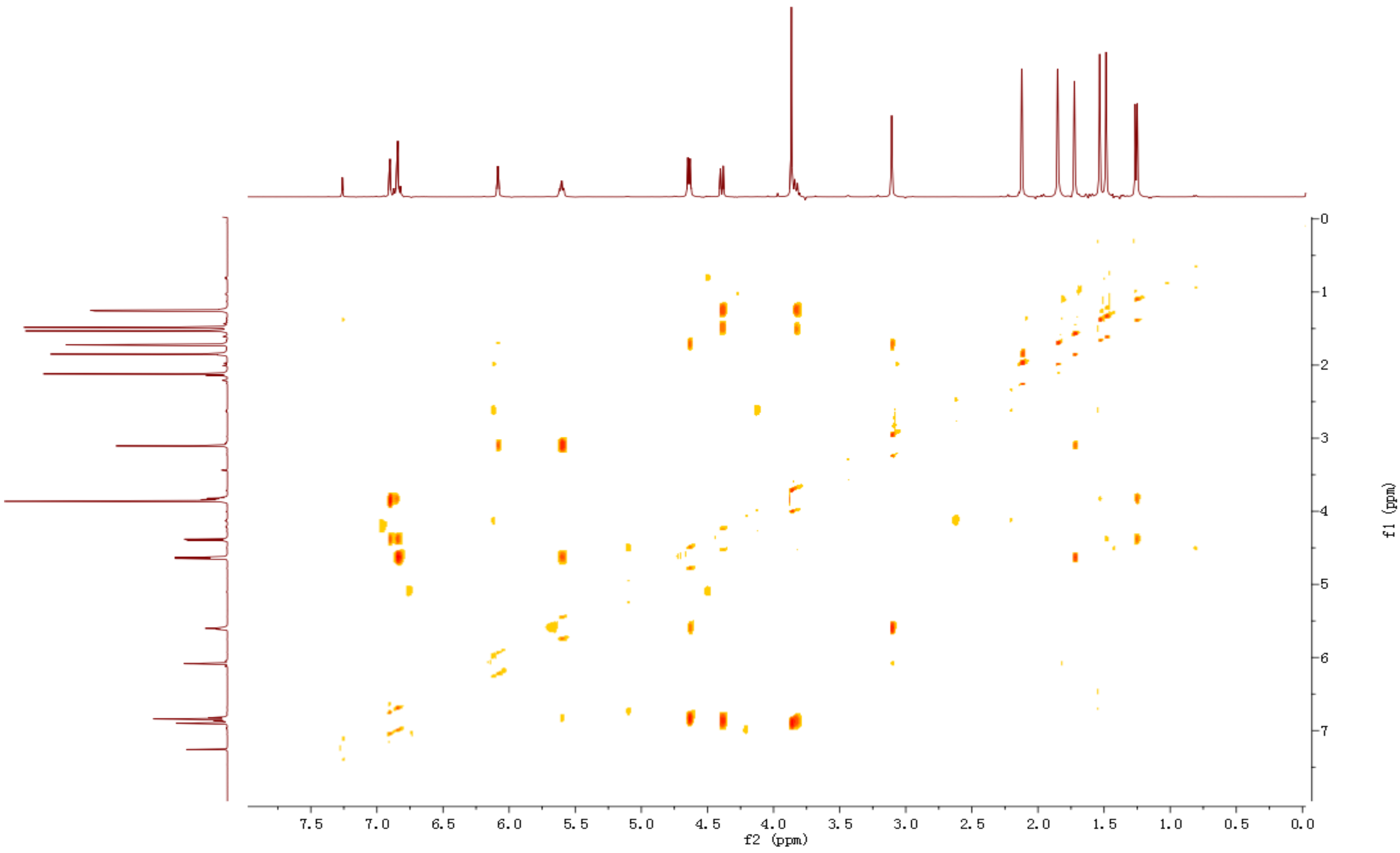

Figure 21S. ^{1}H NMR of compound **3**.

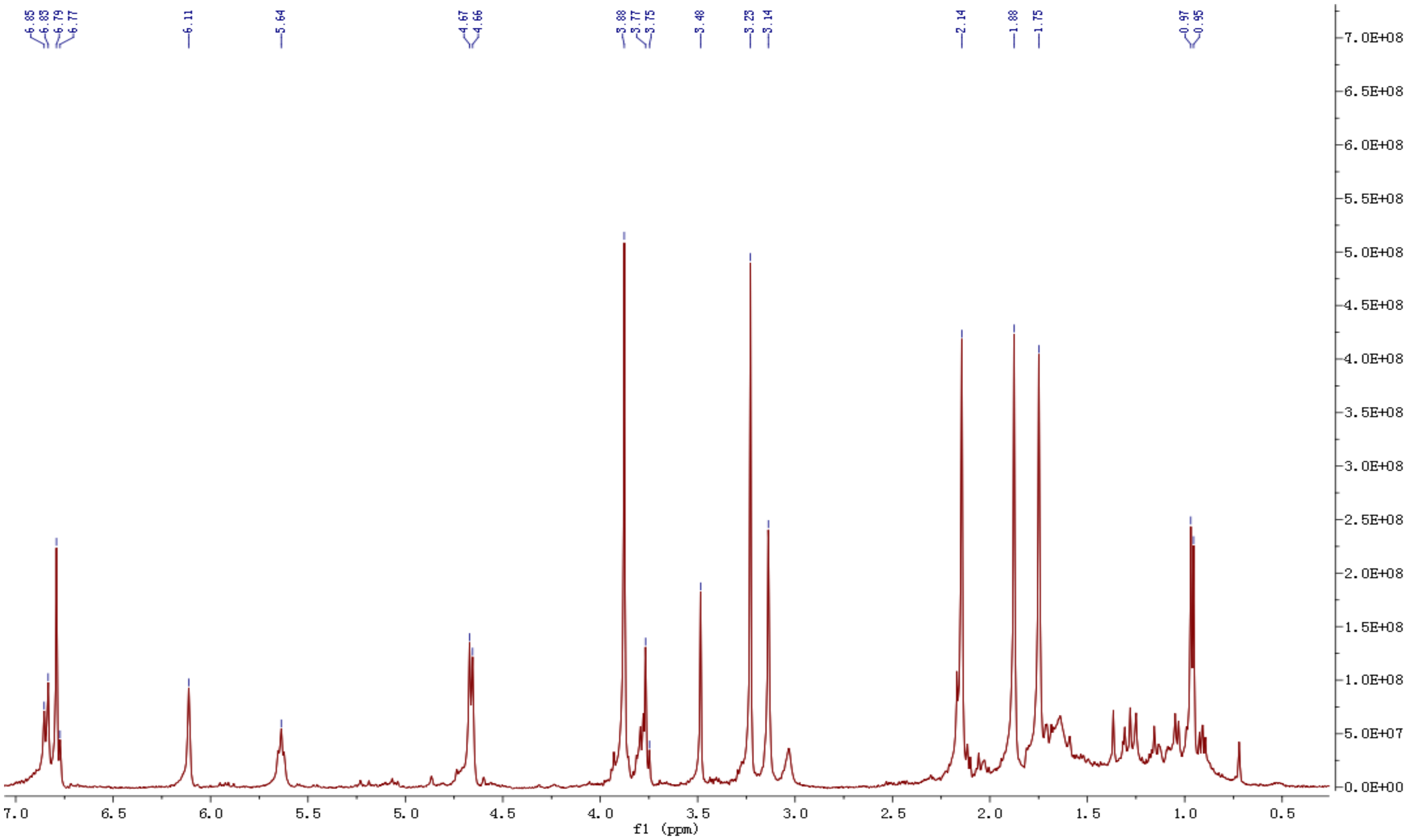

Figure 22S. ^{13}C NMR and DEPT of compound **3**.

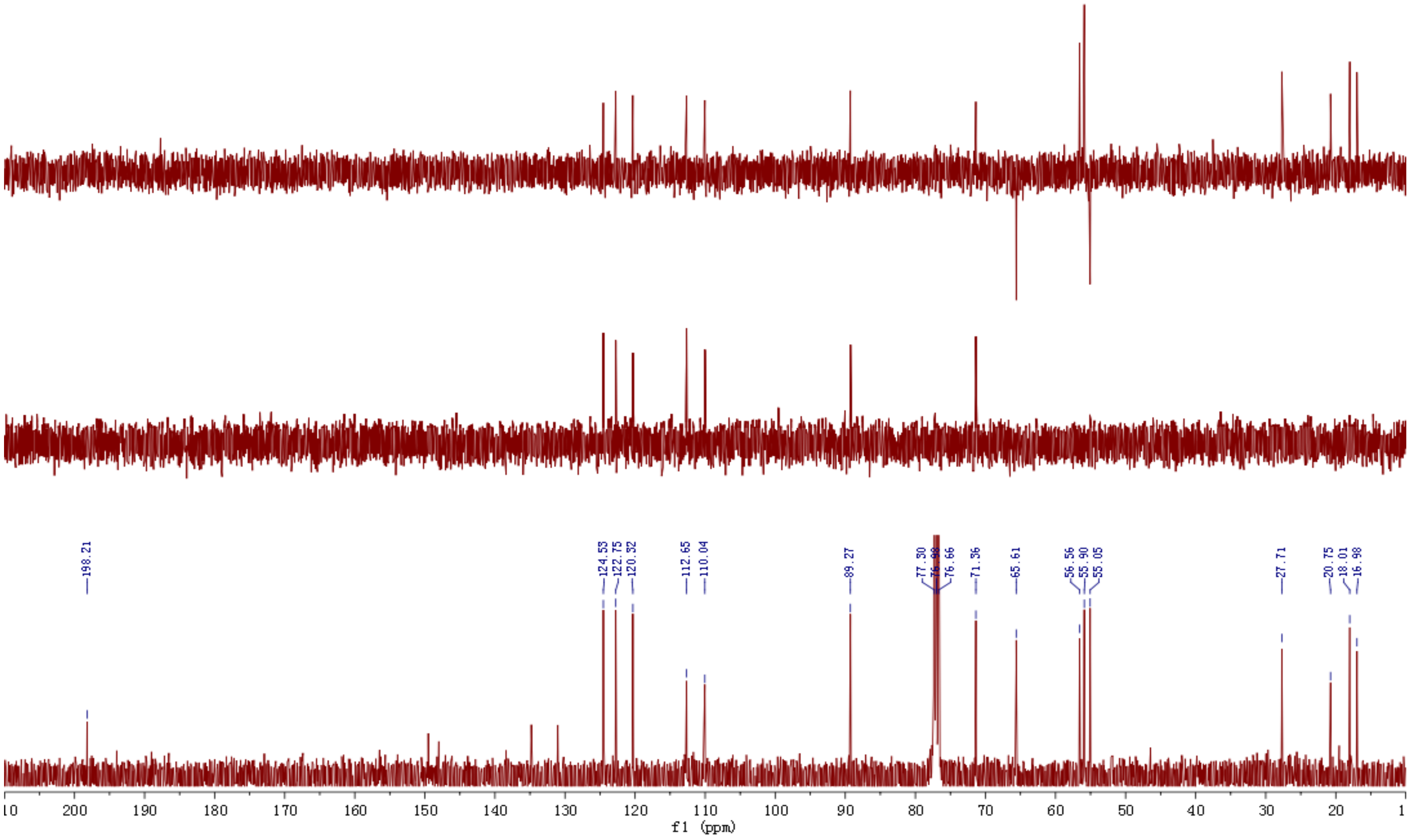

Figure 23S. HSQC of compound **3**.

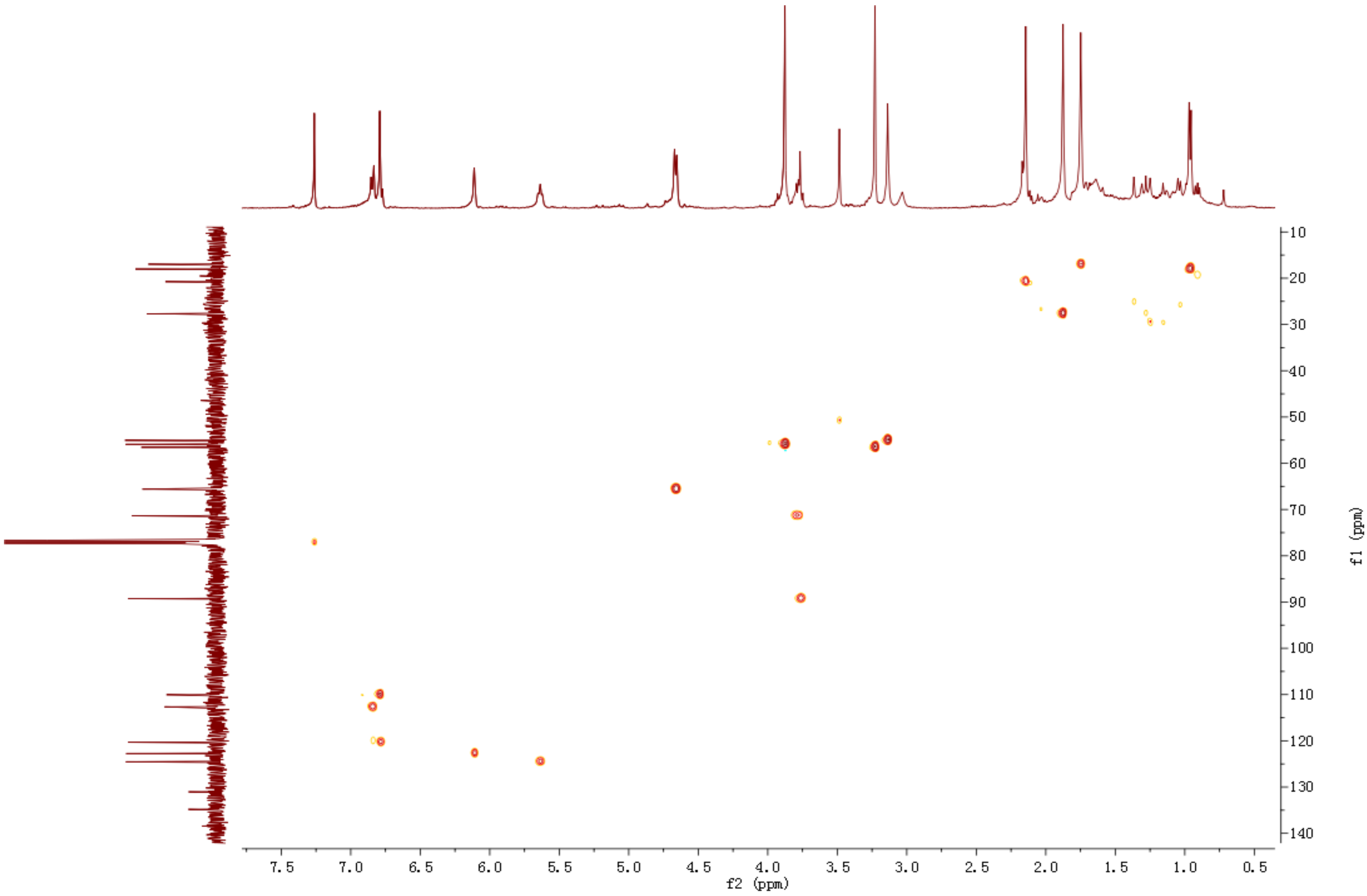

Figure 24S. HMBC of compound **3**.

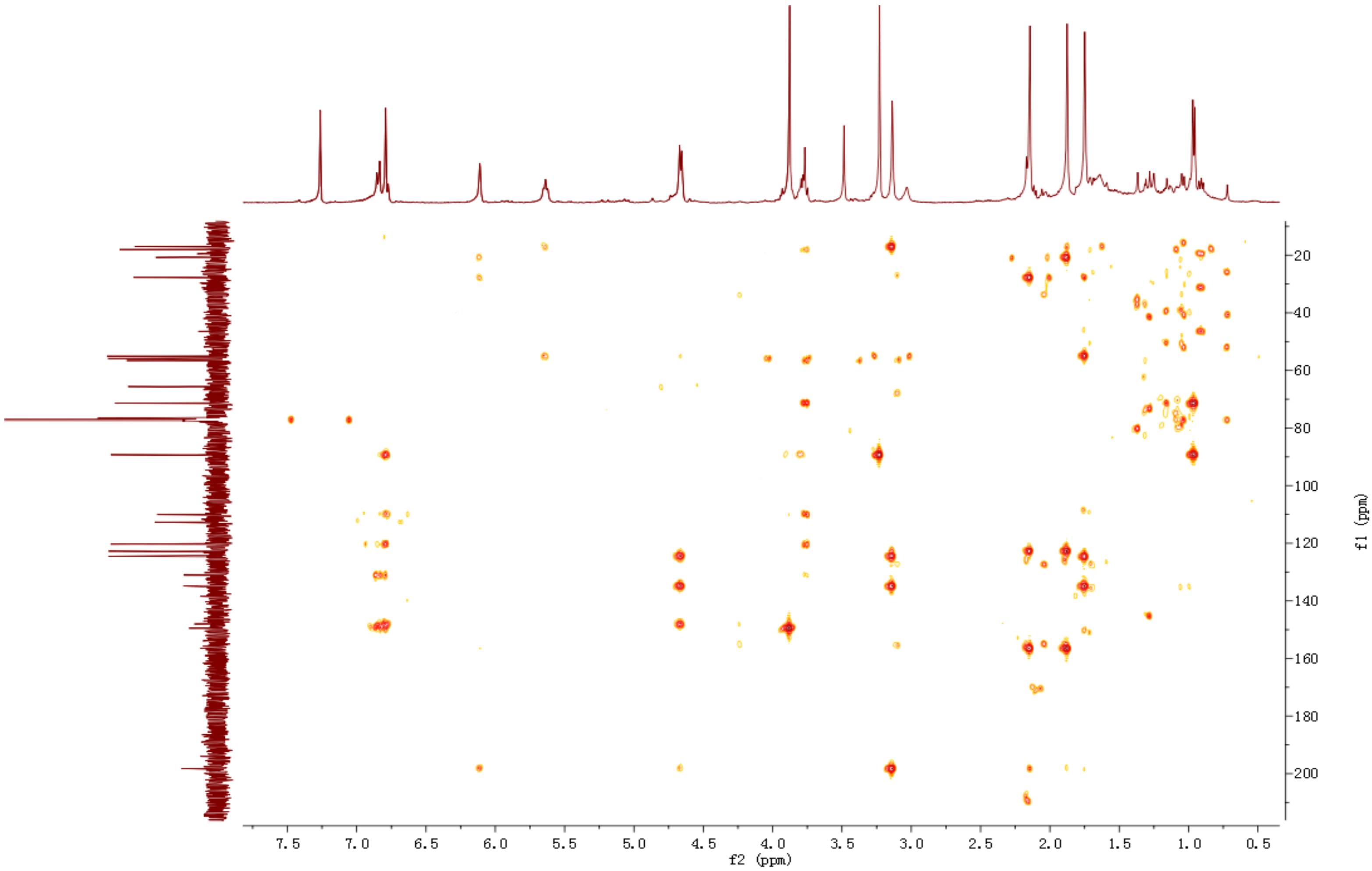

Figure 25S. ^{1}H-^{1}H COSY of compound **3**.

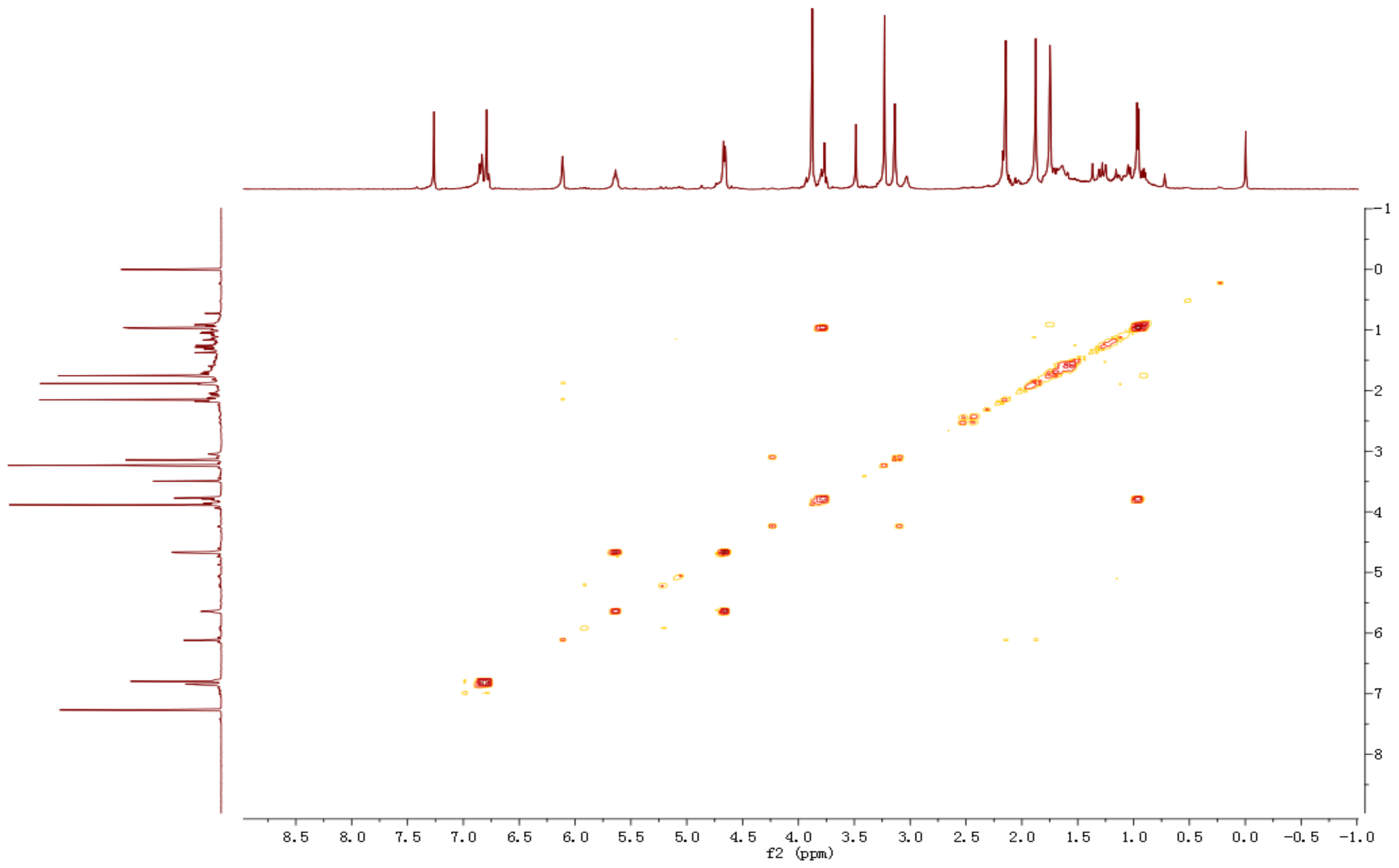

Figure 26S. ROESY of compound **3**.

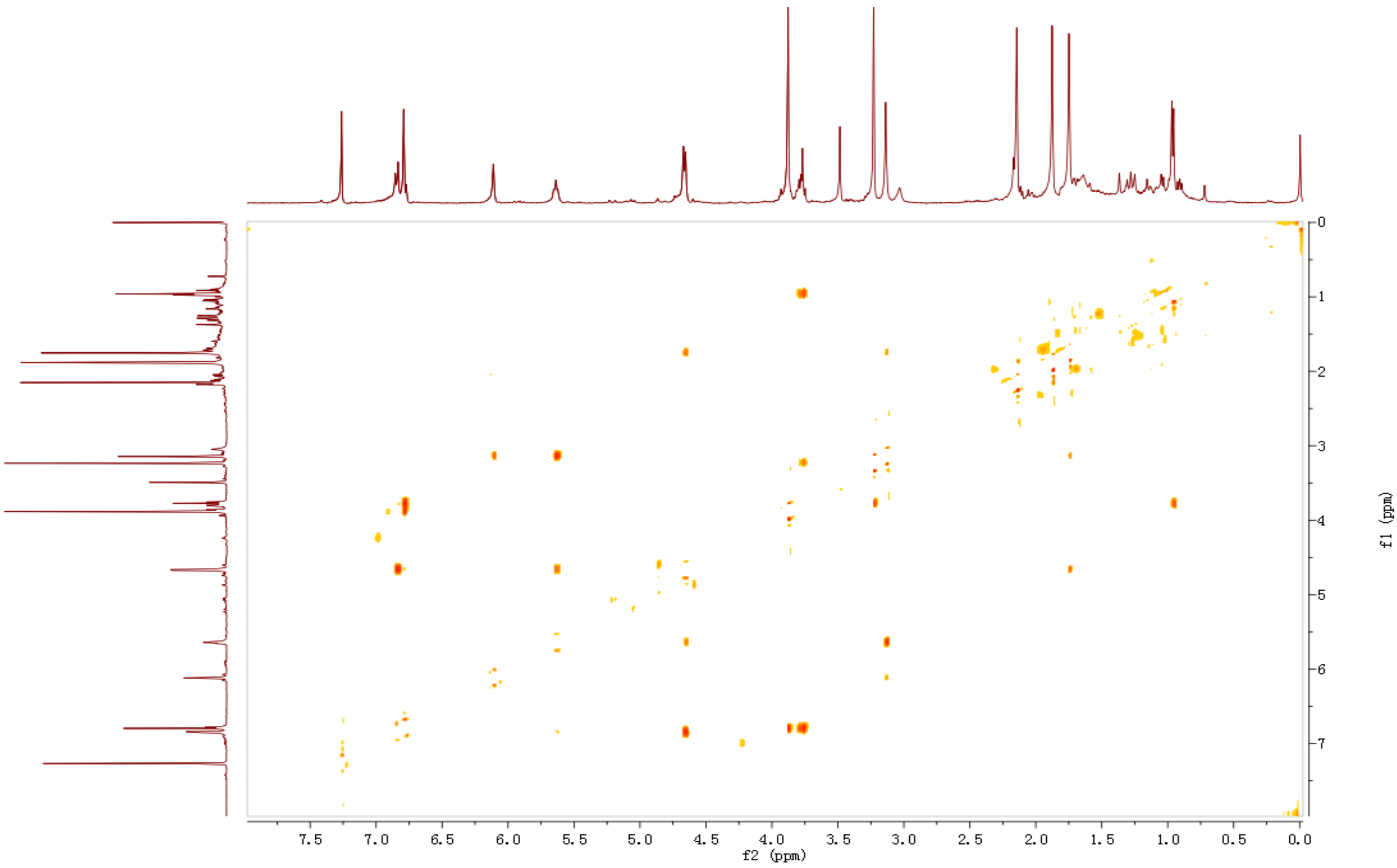

Figure27S. HRESIMS of compound **3**.

Elemental Composition Report

Single Mass Analysis

Tolerance = 10.0 PPM / DBE: min = 0.5, max = 120.0
Selected filters: None

Monoisotopic Mass, Odd and Even Electron Ions
17 formula(e) evaluated with 1 results within limits (up to 51 closest results for each mass)
Elements Used:
C: 0-200 H: 0-400 O: 3-6

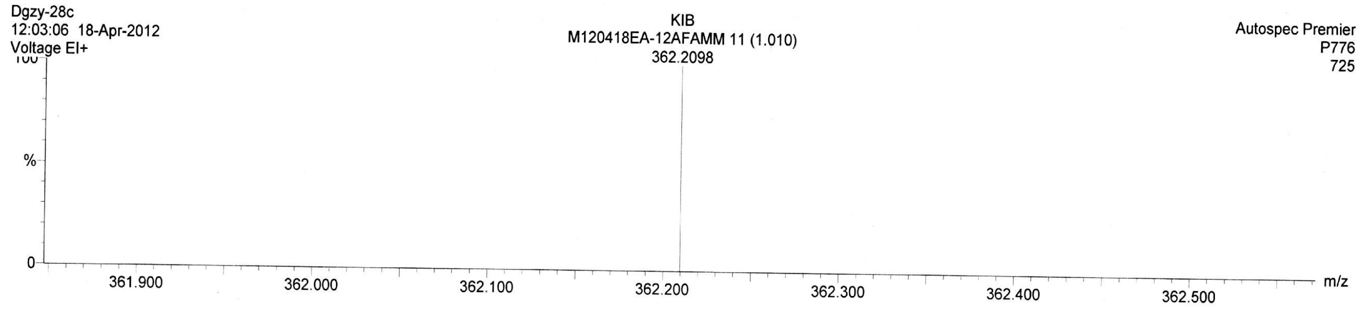

Minimum:				0.5		
Maximum:		40.0	10.0	120.0		
Mass	Calc. Mass	mDa	PPM	DBE	i-FIT	Formula
362.2098	362.2093	0.5	1.4	7.0	5546375.0	C21 H30 O5

Figure 28S. ^{1}H NMR of compound **4**.

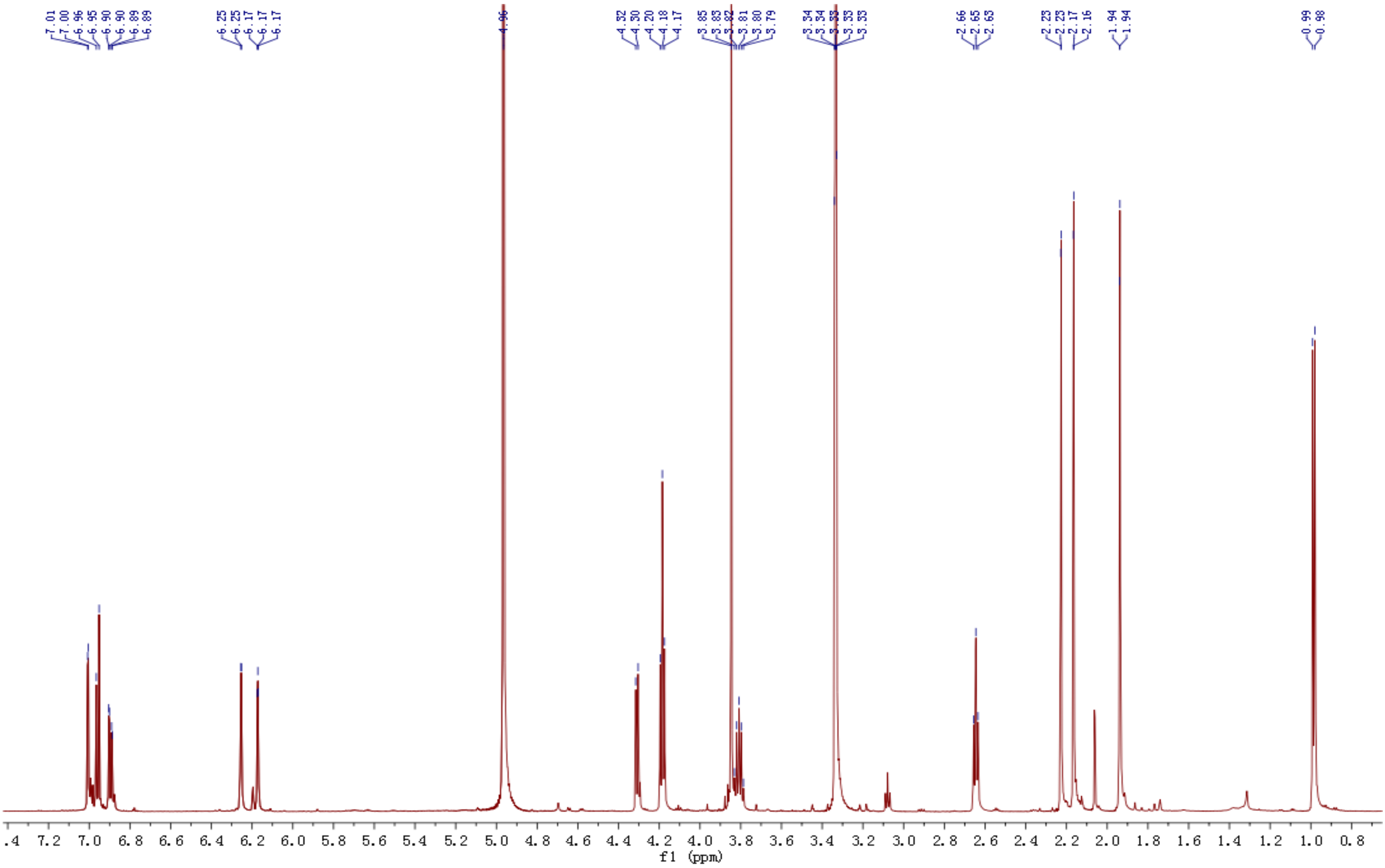

Figure 29S. ^{13}C NMR and DEPT of compound **4**.

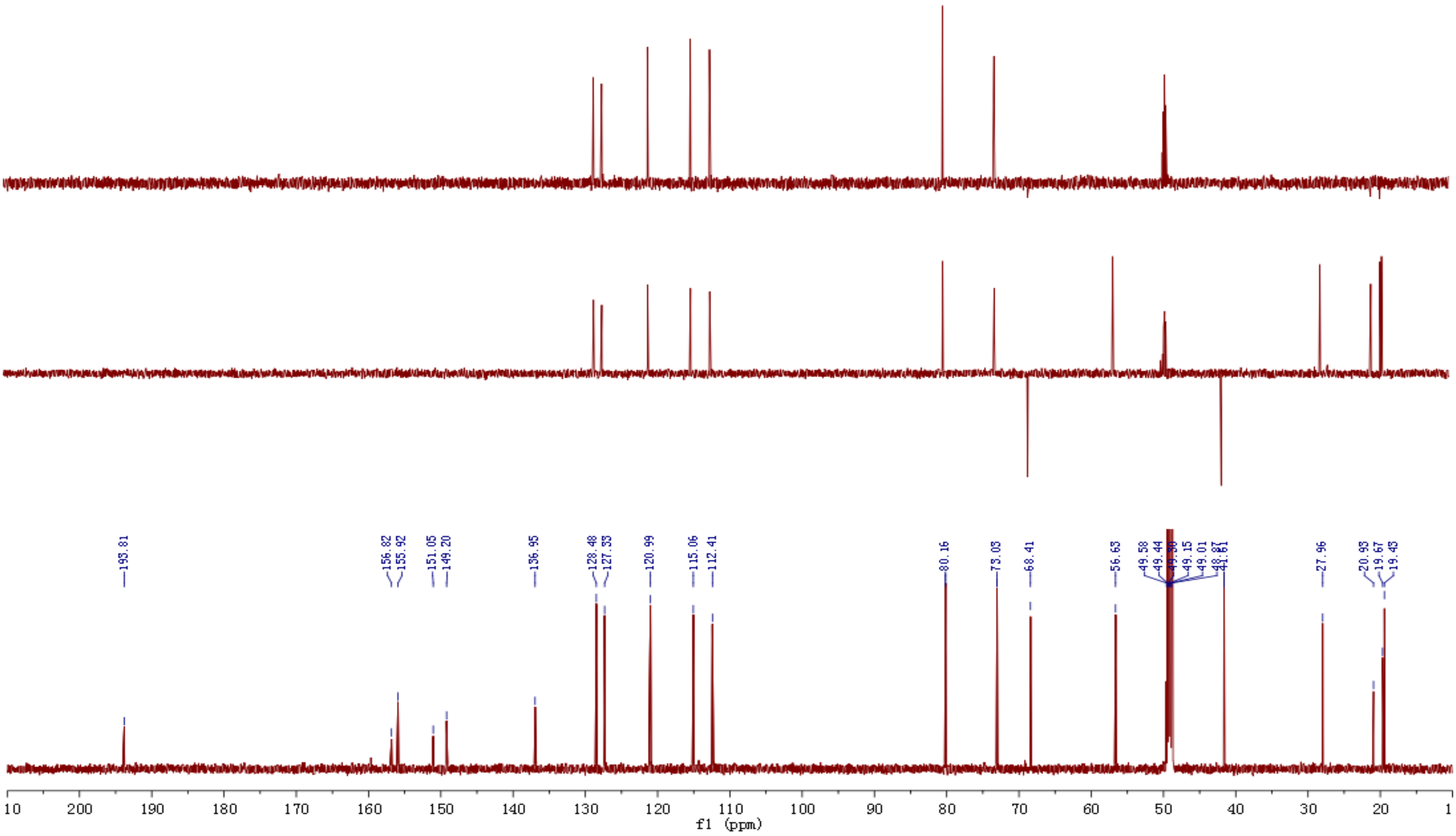

Figure 30S. HSQC of compound **4**.

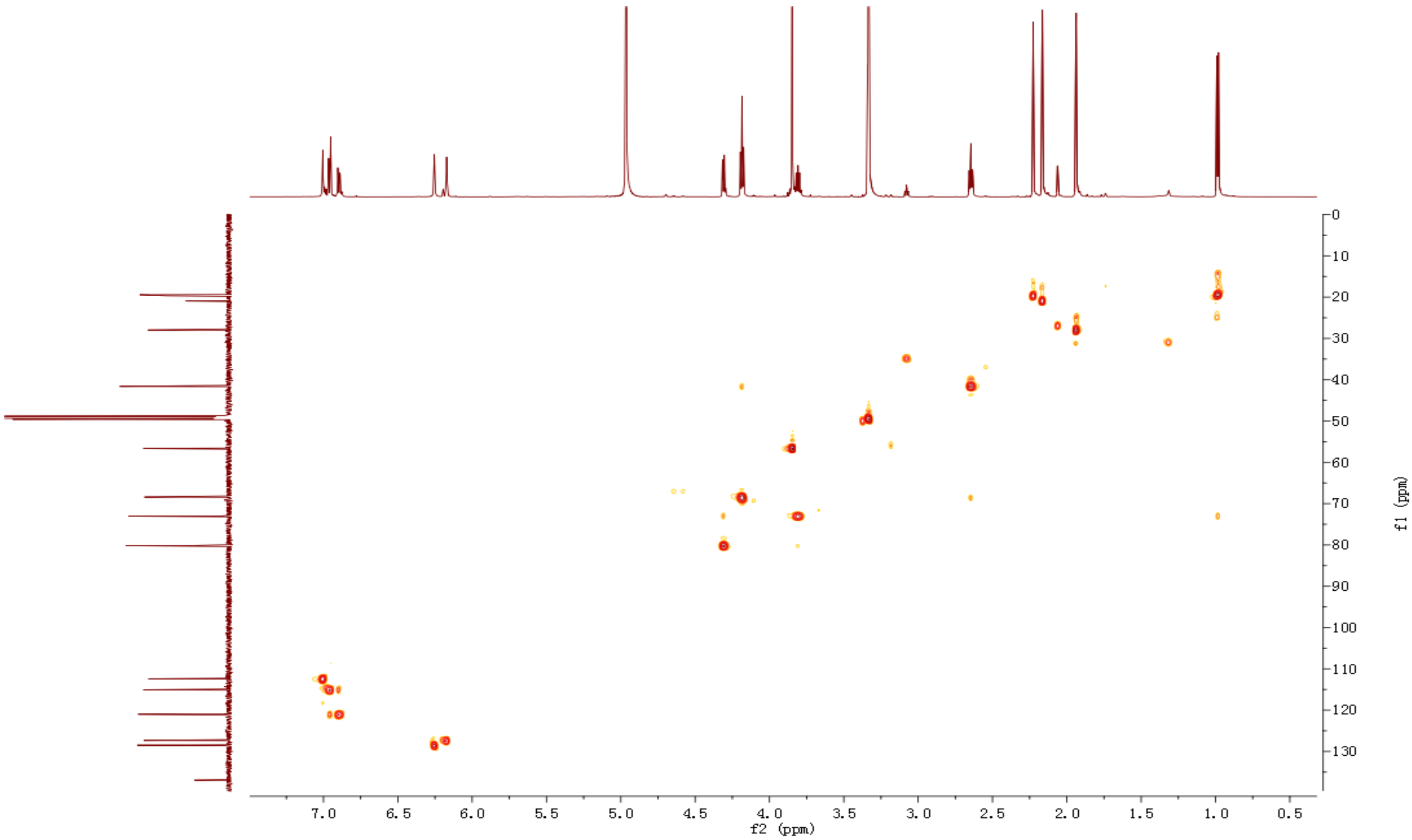

Figure 31S. HMBC of compound **4**.

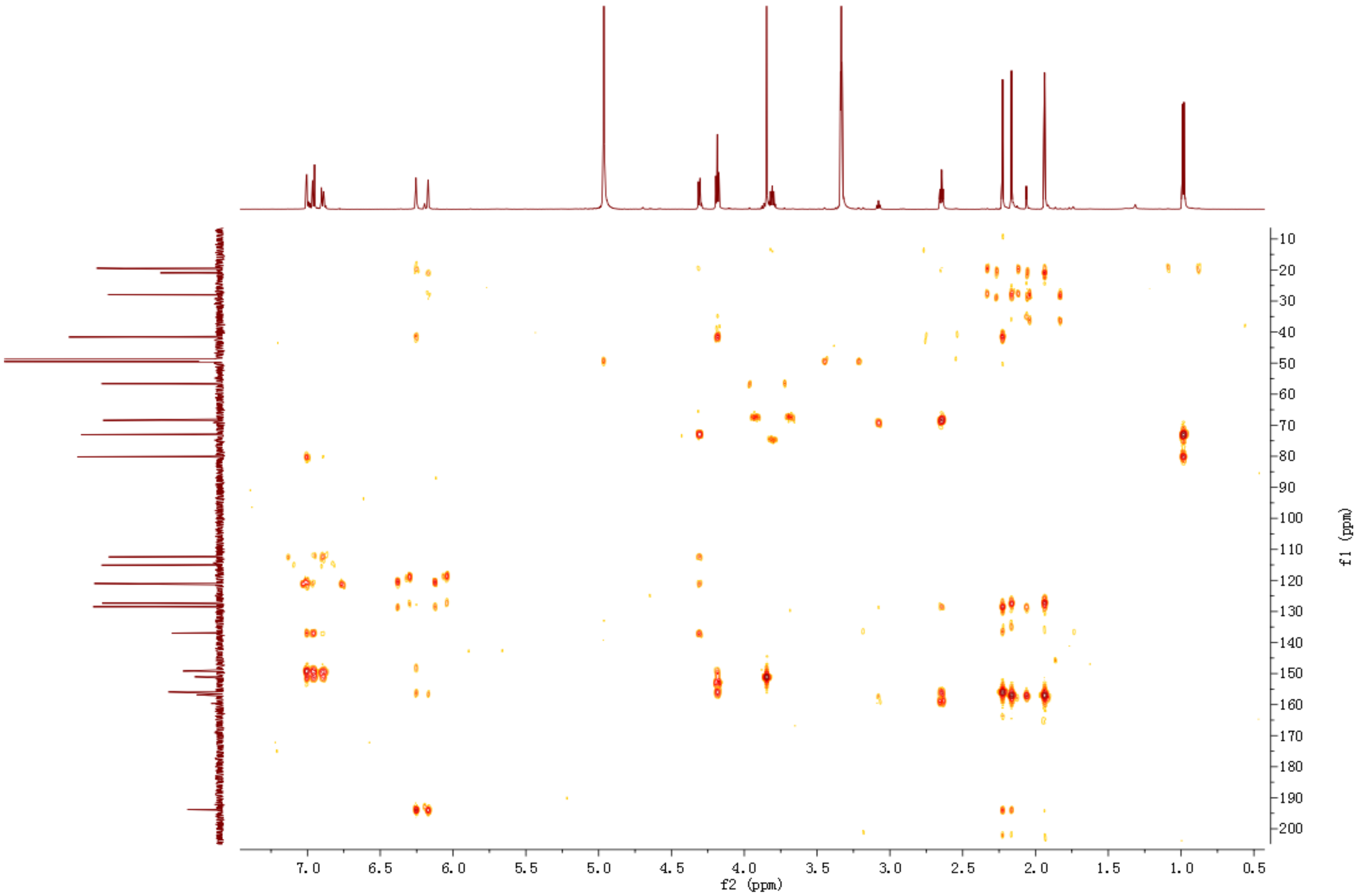

Figure 32S. ^{1}H-^{1}H COSY of compound **4**.

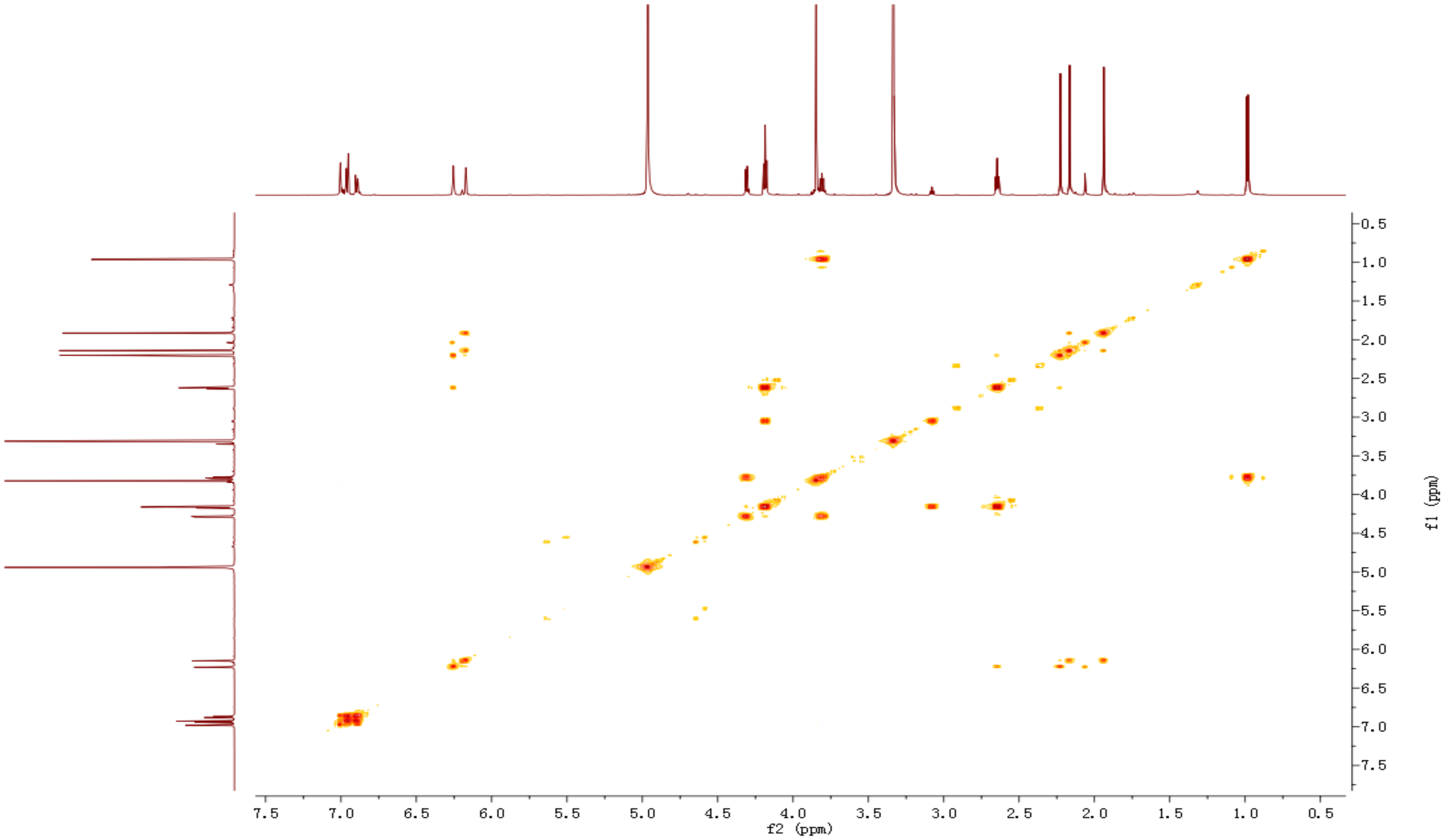

Figure 33S. ROESY of compound **4**.

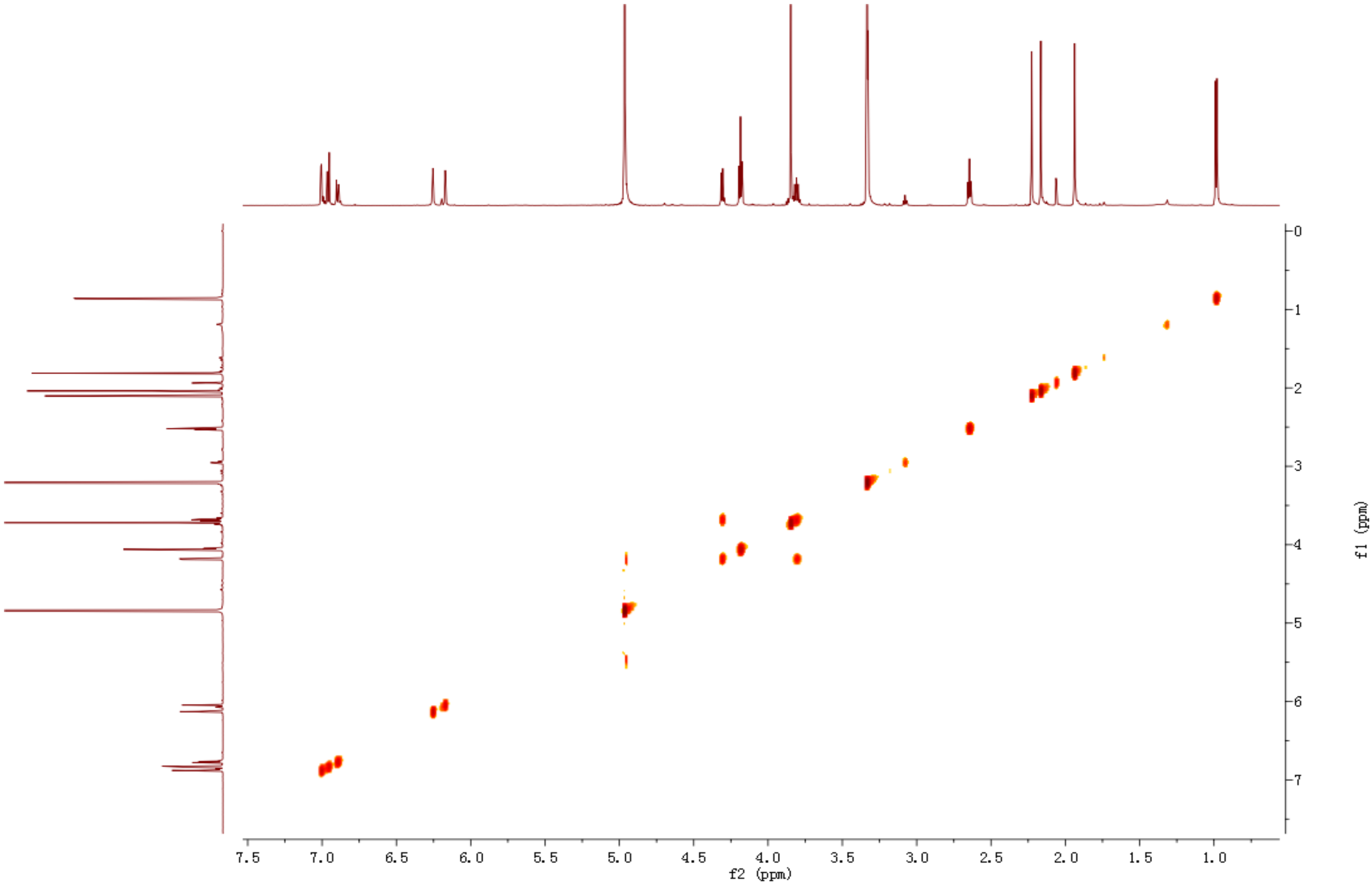

Figure34S. HRESIMS of compound **4**.

Elemental Composition Report

Page 1

Single Mass Analysis
Tolerance = 10.0 PPM / DBE: min = 0.5, max = 120.0
Selected filters: None

Monoisotopic Mass, Odd and Even Electron Ions
17 formula(e) evaluated with 1 results within limits (up to 51 closest results for each mass)
Elements Used:
C: 0-200 H: 0-400 O: 3-6

Dgzy-25c
11:54:27 18-Apr-2012
Voltage EI+

KIB
M120418EA-10AFAMM 12 (1.102)

Autospec Premier
P776
1.98e3

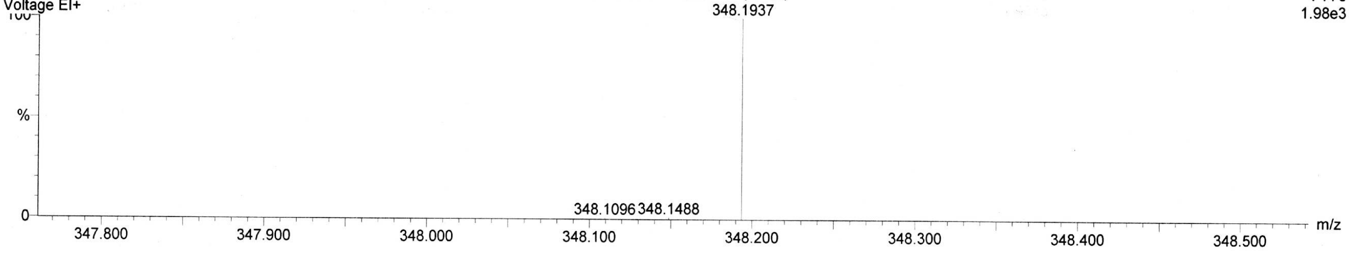

Minimum:				0.5		
Maximum:		40.0	10.0	120.0		
Mass	Calc. Mass	mDa	PPM	DBE	i-FIT	Formula
348.1937	348.1937	0.0	0.0	7.0	5547000.5	C20 H28 O5

Figure 35S. ^{1}H NMR of compound **5**.

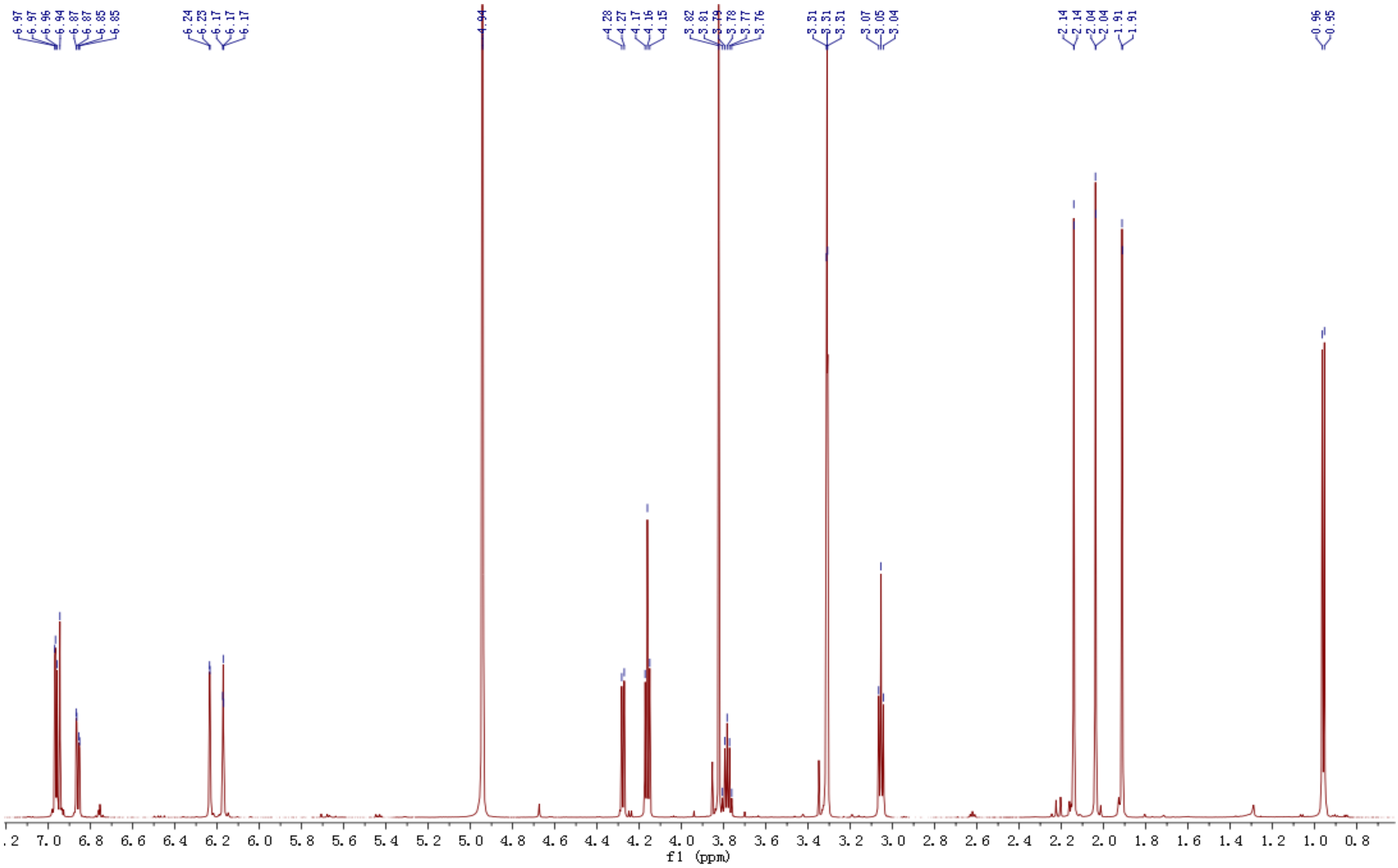

Figure 36S. ^{13}C NMR and DEPT of compound **5**.

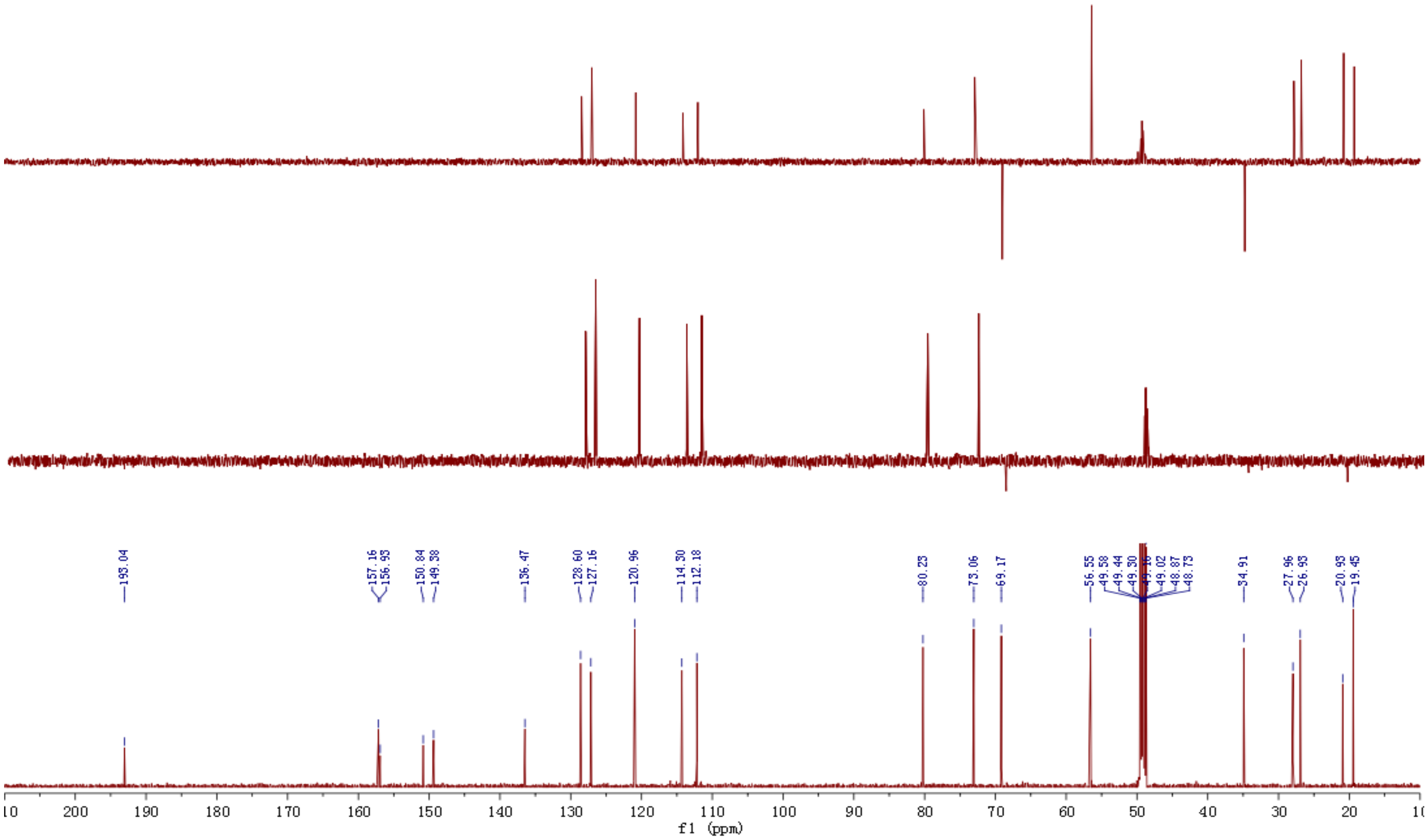

Figure 37S. HSQC of compound **5**.

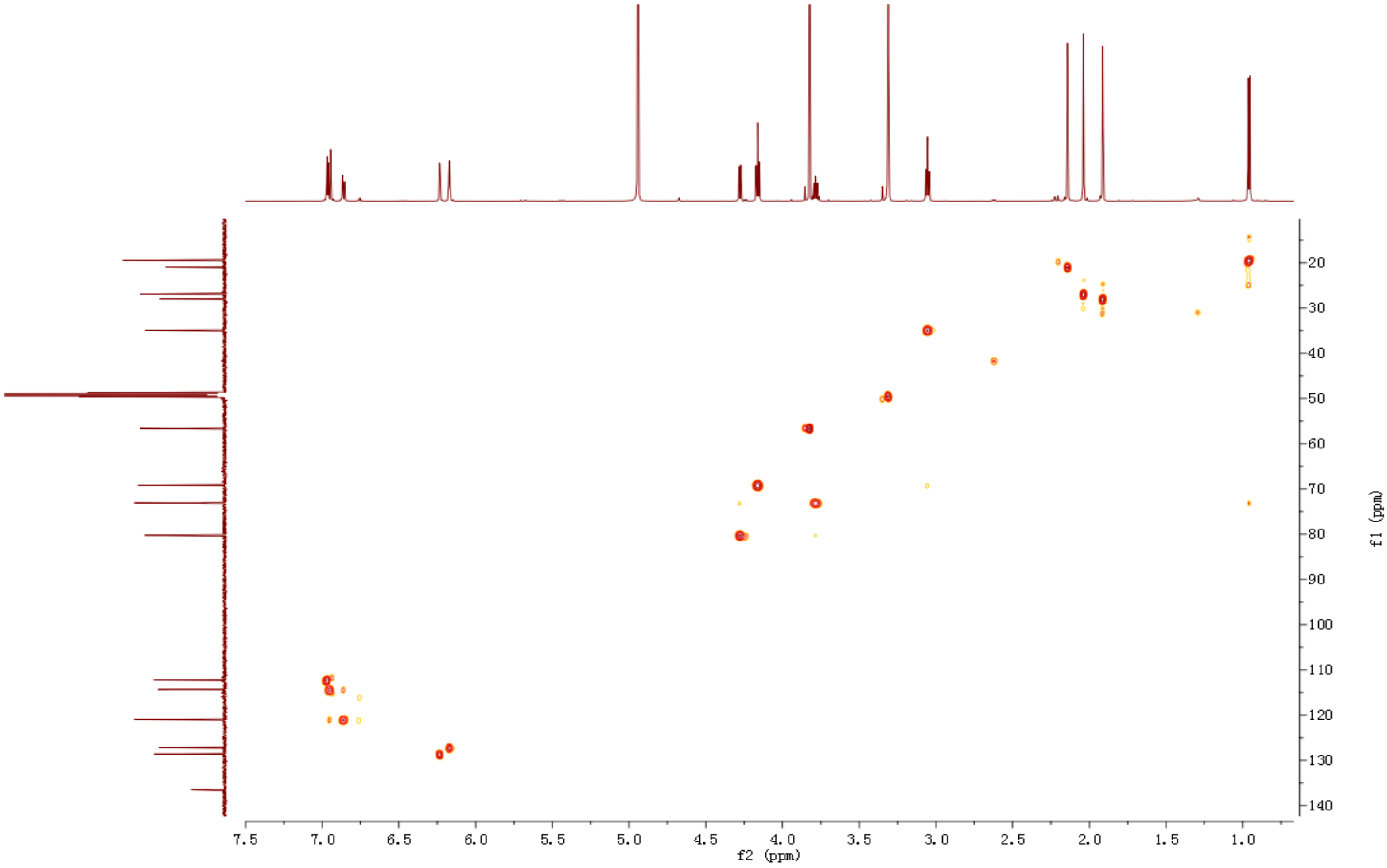

Figure 38S. HMBC of compound **5**.

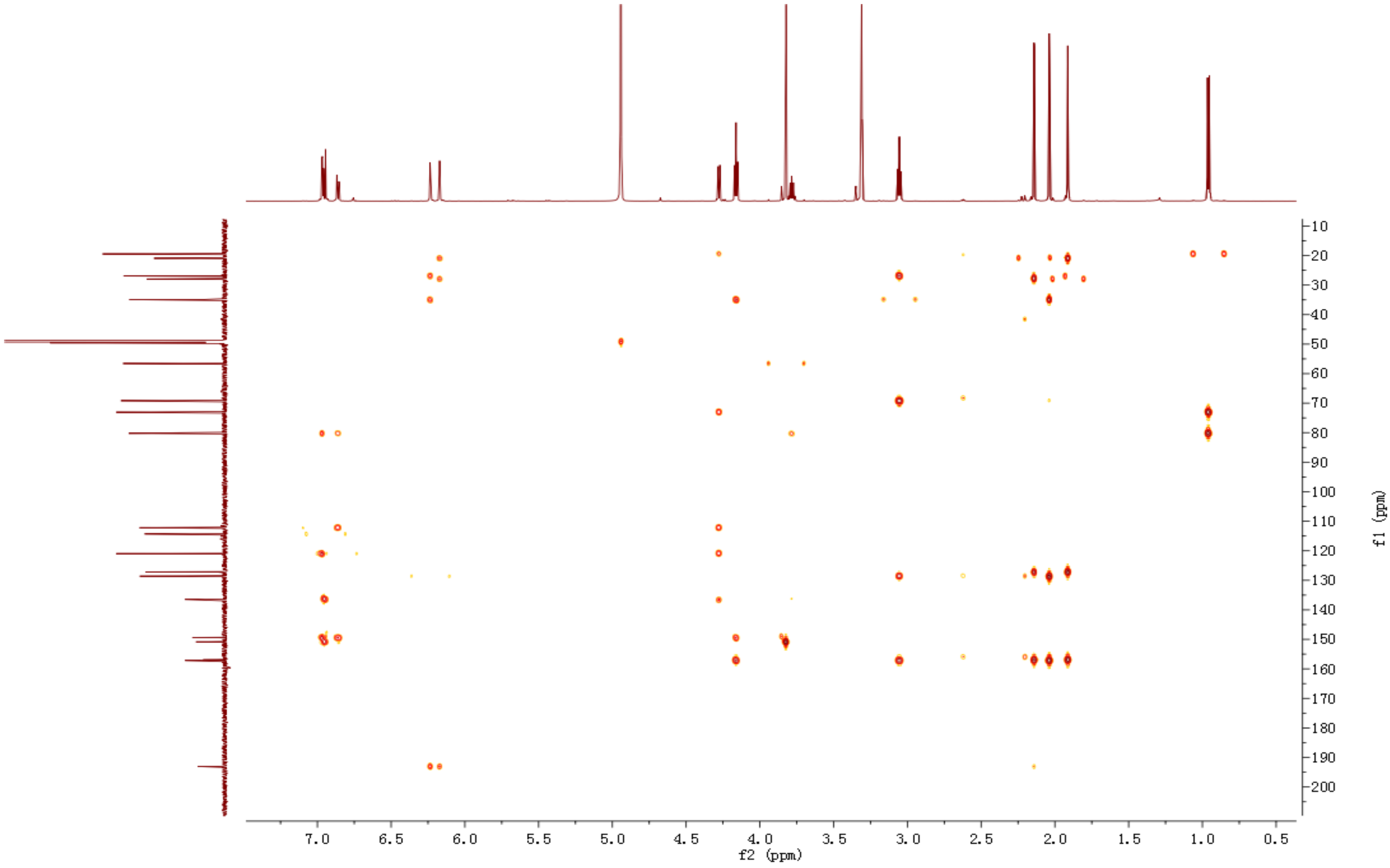

Figure 39S. COSY of compound **5**.

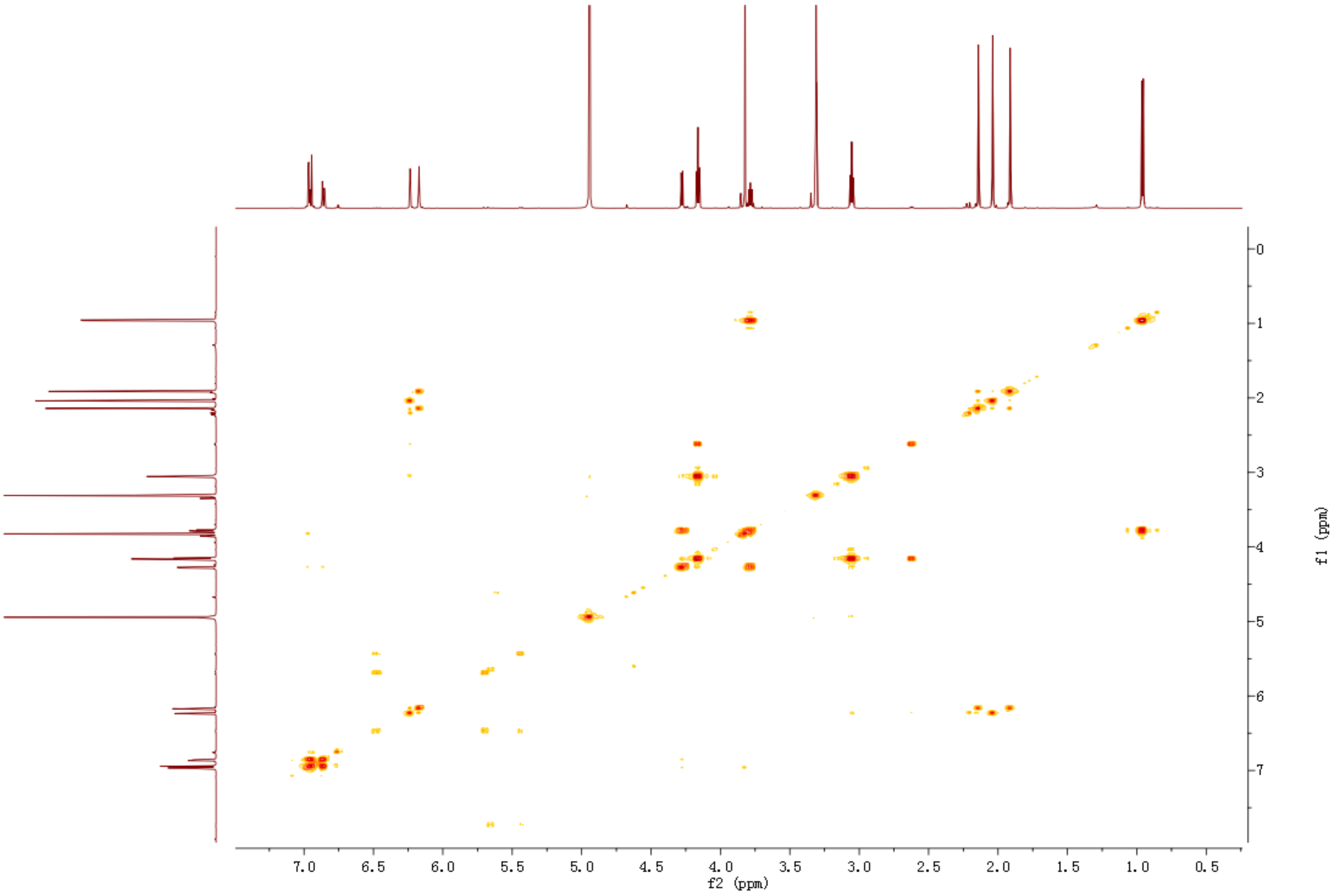

Figure 40S. ROESY of compound **5**.

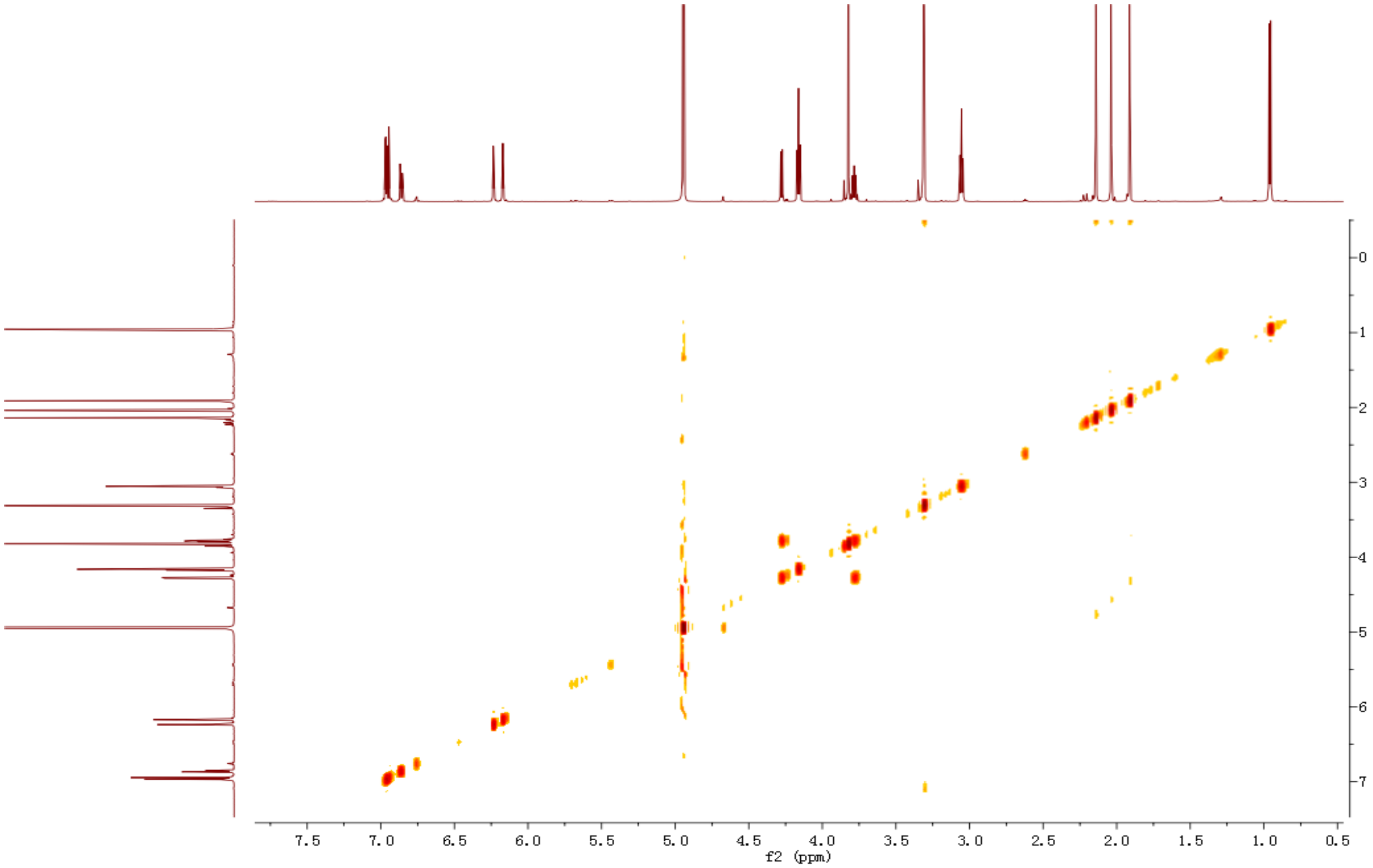

Figure41S. HRESIMS of compound **5**.

Elemental Composition Report **Page 1**

Single Mass Analysis
Tolerance = 10.0 PPM / DBE: min = 0.5, max = 120.0
Selected filters: None

Monoisotopic Mass, Odd and Even Electron Ions
17 formula(e) evaluated with 1 results within limits (up to 51 closest results for each mass)
Elements Used:
C: 0-200 H: 0-400 O: 3-6

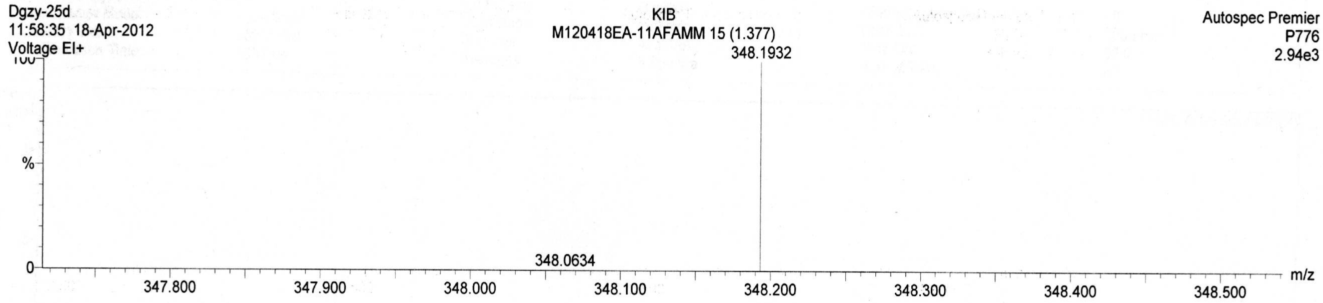

Minimum:				0.5		
Maximum:		40.0	10.0	120.0		
Mass	Calc. Mass	mDa	PPM	DBE	i-FIT	Formula
348.1932	348.1937	-0.5	-1.4	7.0	5547479.0	C20 H28 O5

Figure 42S. ^{1}H NMR of compound **6**.

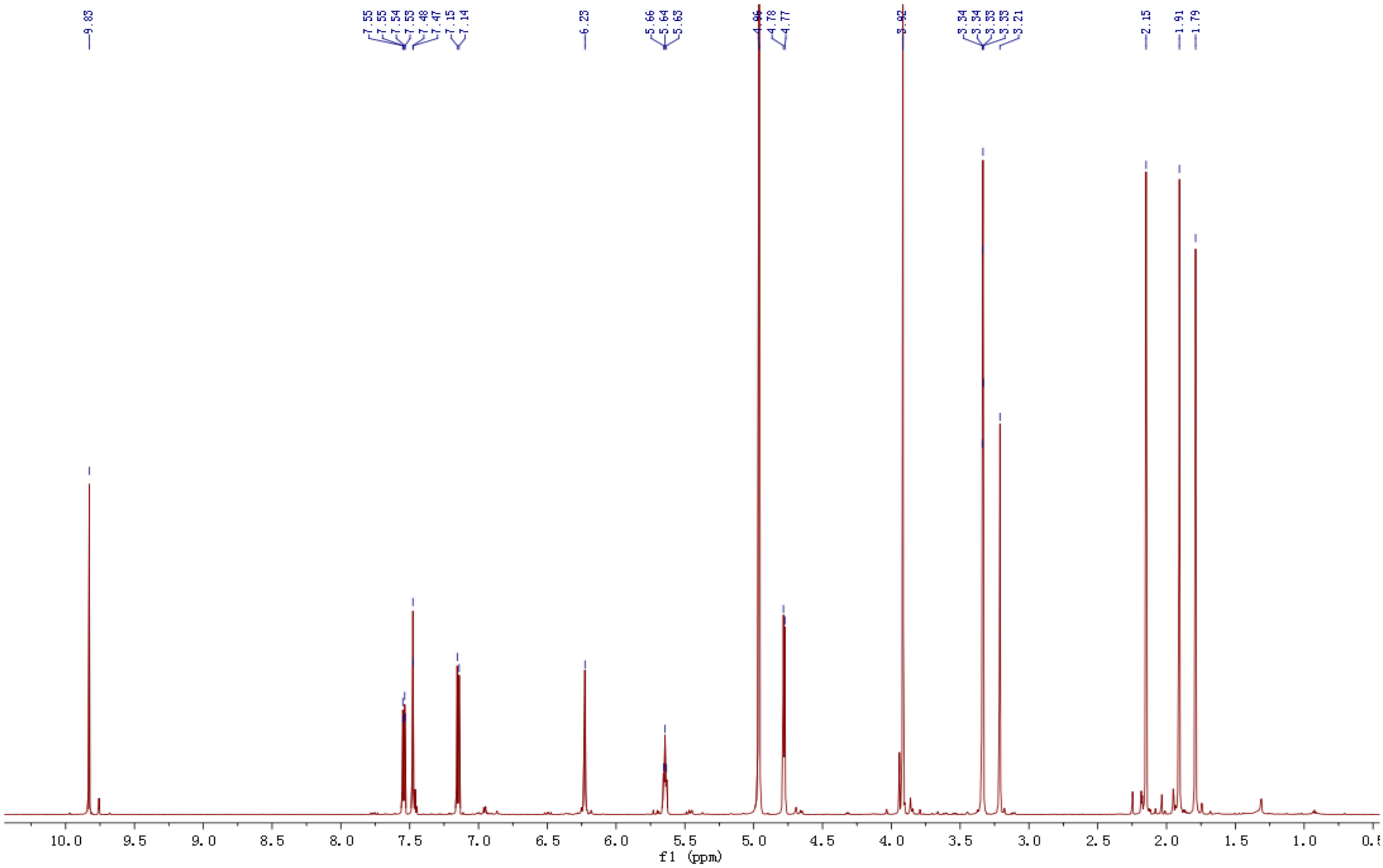

Figure 43S. ^{13}C NMR and DEPT of compound **6**.

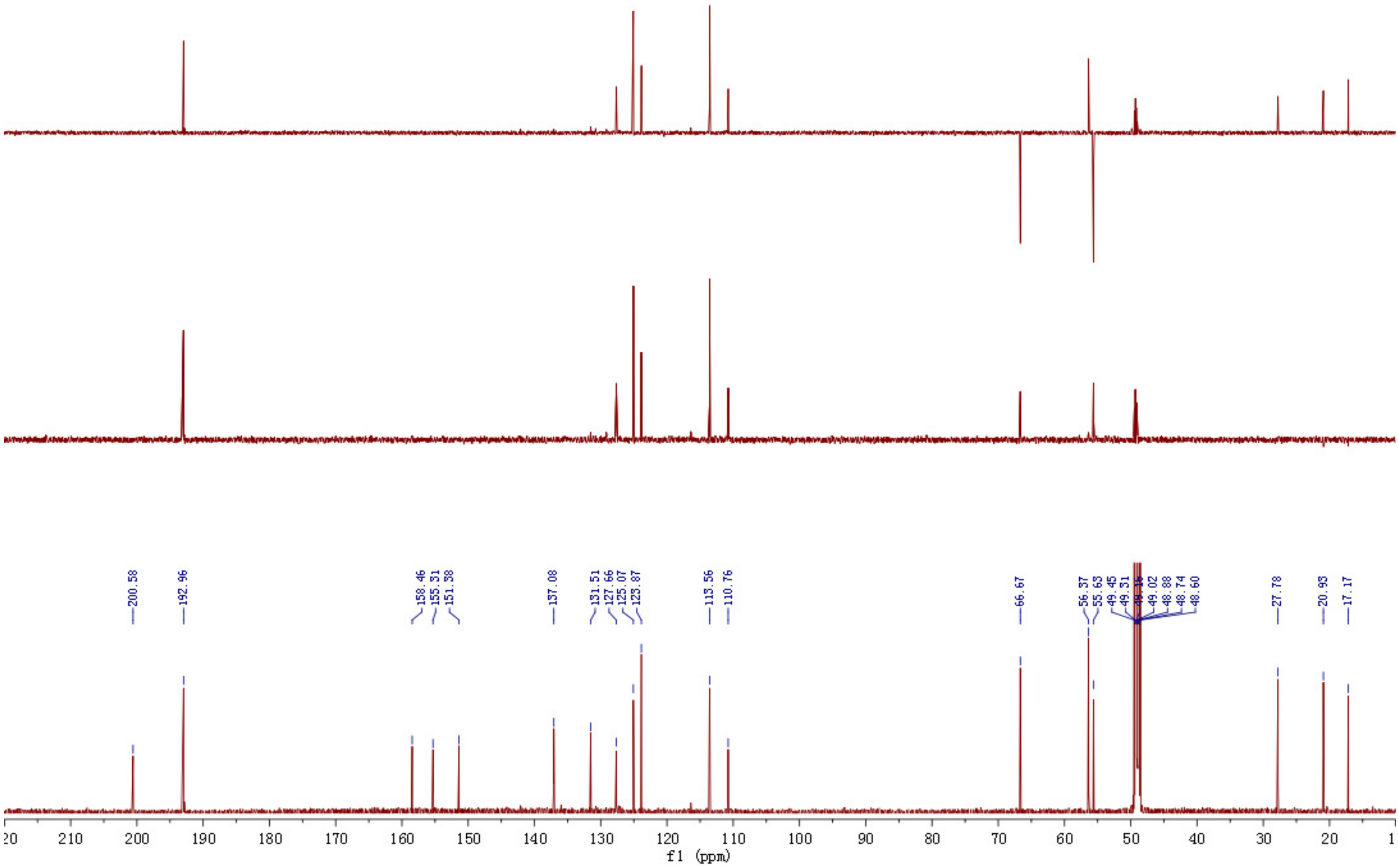

Figure 44S. HSQC of compound **6**.

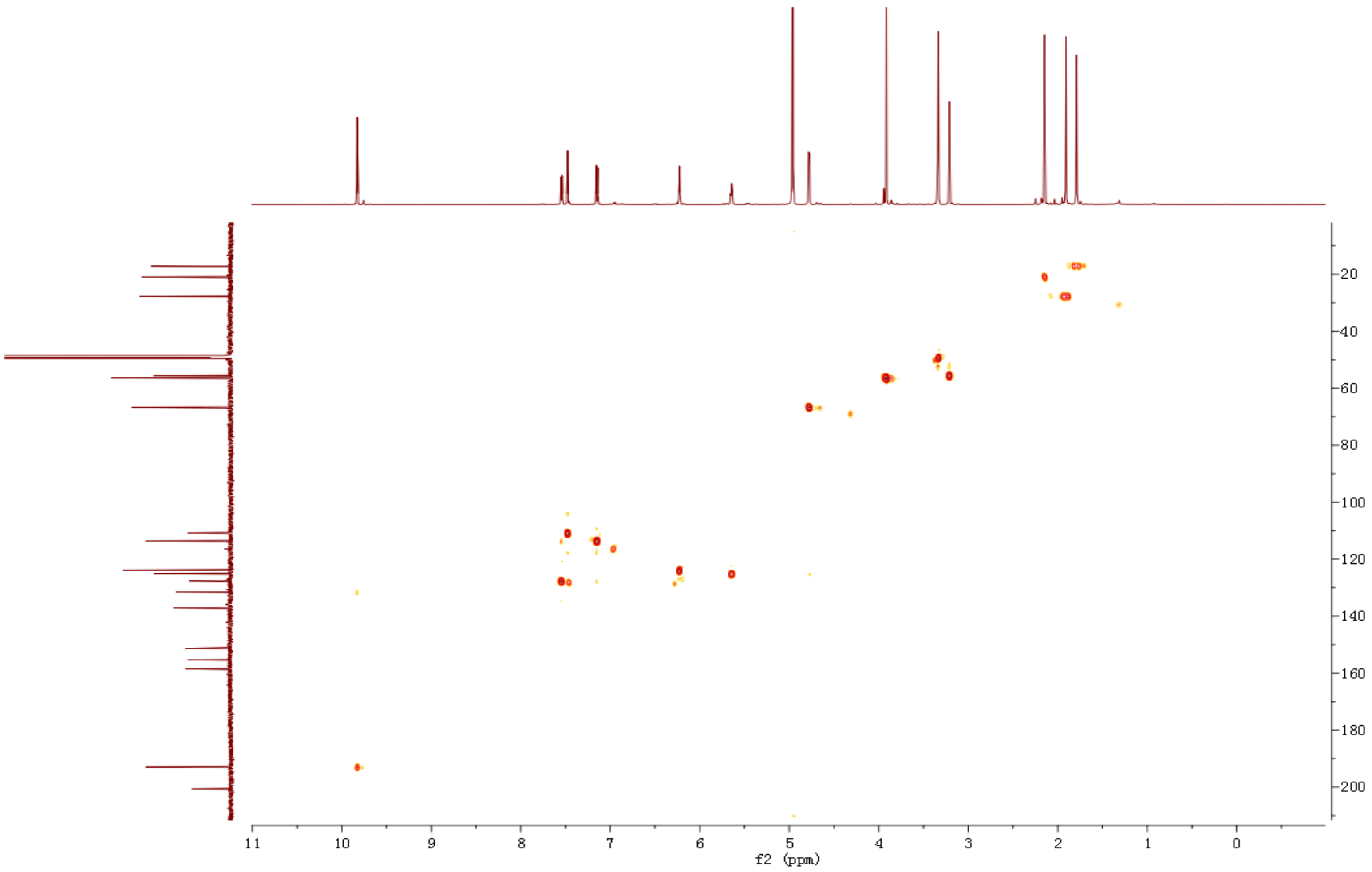

Figure 45S. HMBC of compound **6**.

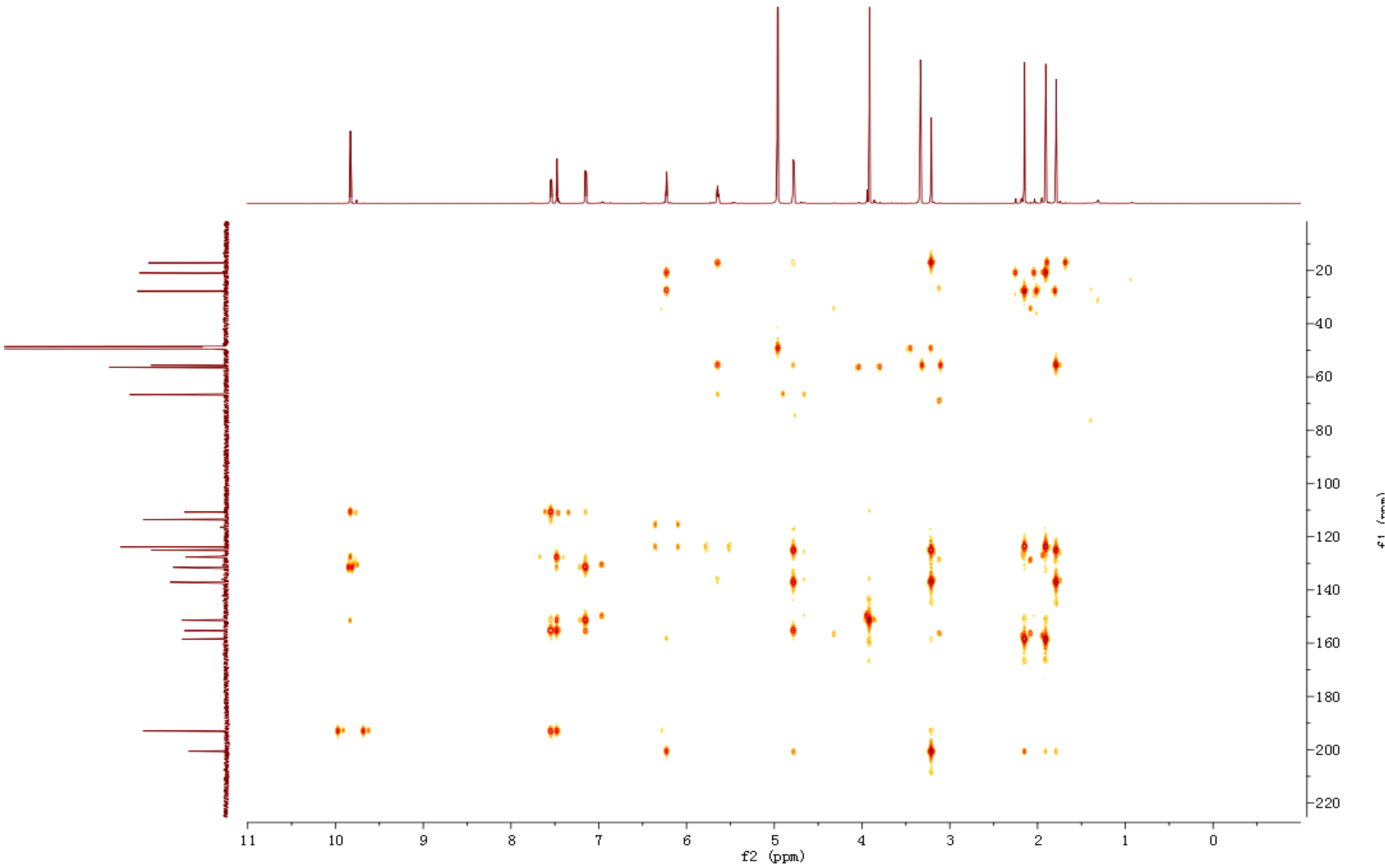

Figure 46S. ^{1}H-^{1}H COSY of compound **6**.

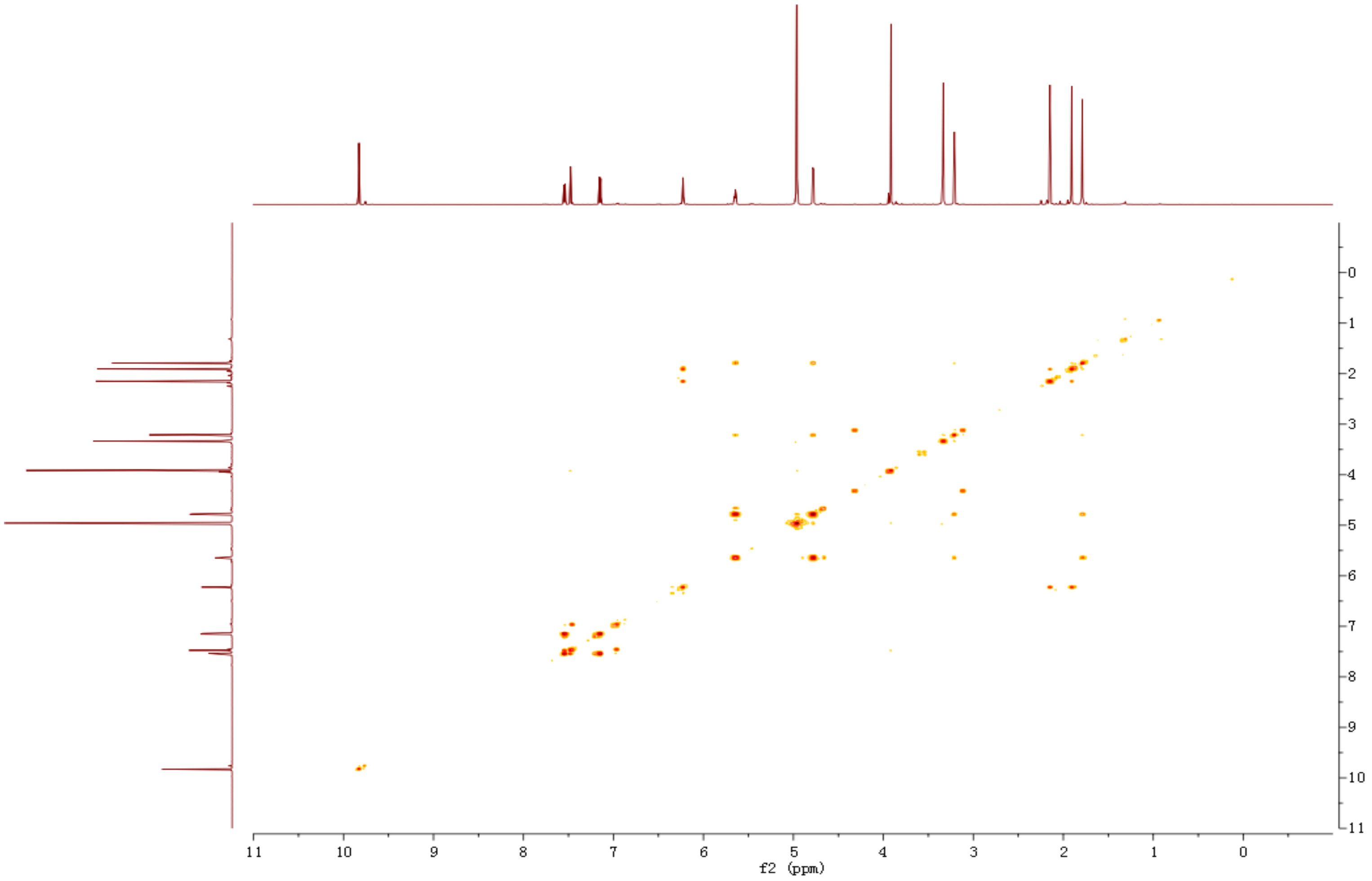

Figure 47S. ROESY of compound **6**.

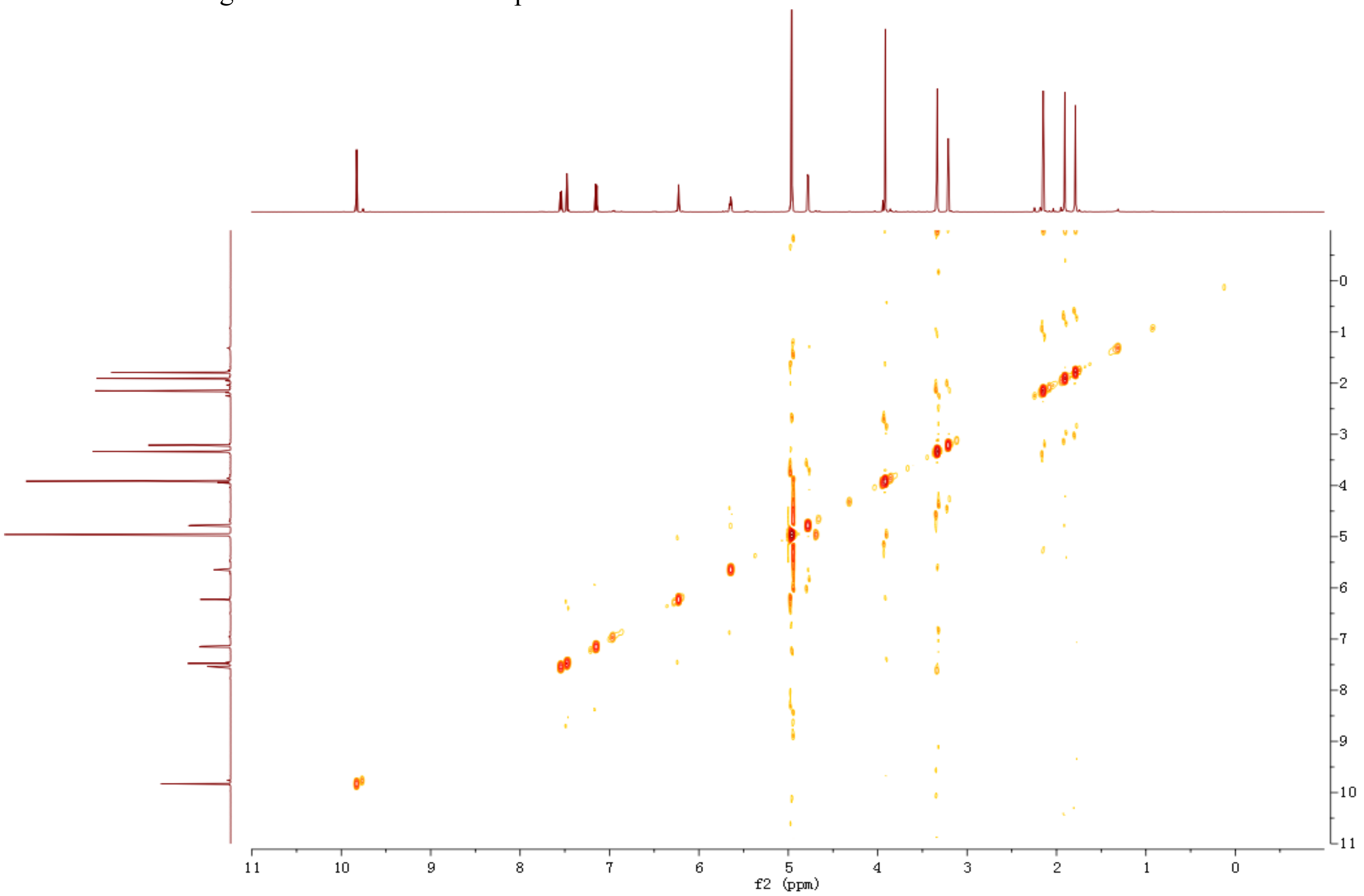

Figure 48S. HRESIMS of compound **6**.

Elemental Composition Report

Single Mass Analysis (displaying only valid results)
Tolerance = 10.0 PPM / DBE: min = 0.5, max = 60.0
Selected filters: None

Monoisotopic Mass, Odd and Even Electron Ions
13 formula(e) evaluated with 1 results within limits (up to 51 closest results for each mass)
Elements Used:
C: 0-200 H: 0-400 O: 3-6

Dgzy-12 KIB Autospec Premier
11:02:14 09-Apr-2012 M120409EA-01AFAMM 13 (1.194) P776
Voltage EI+ 3.68e3

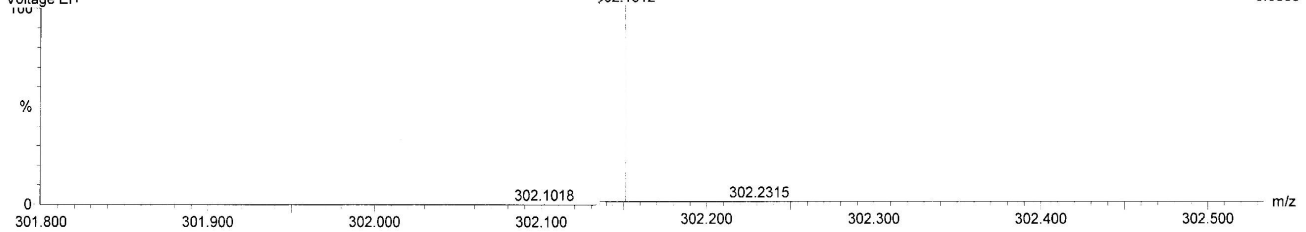

		mDa	PPM	DBE		
Minimum:				0.5		
Maximum:		73.0	10.0	60.0		

Mass	Calc. Mass	mDa	PPM	DBE	i-FIT	Formula
302.1512	302.1518	-0.6	-2.0	8.0	5547847.5	C18 H22 O4

Figure 49S. ^{1}H NMR of compound **7**.

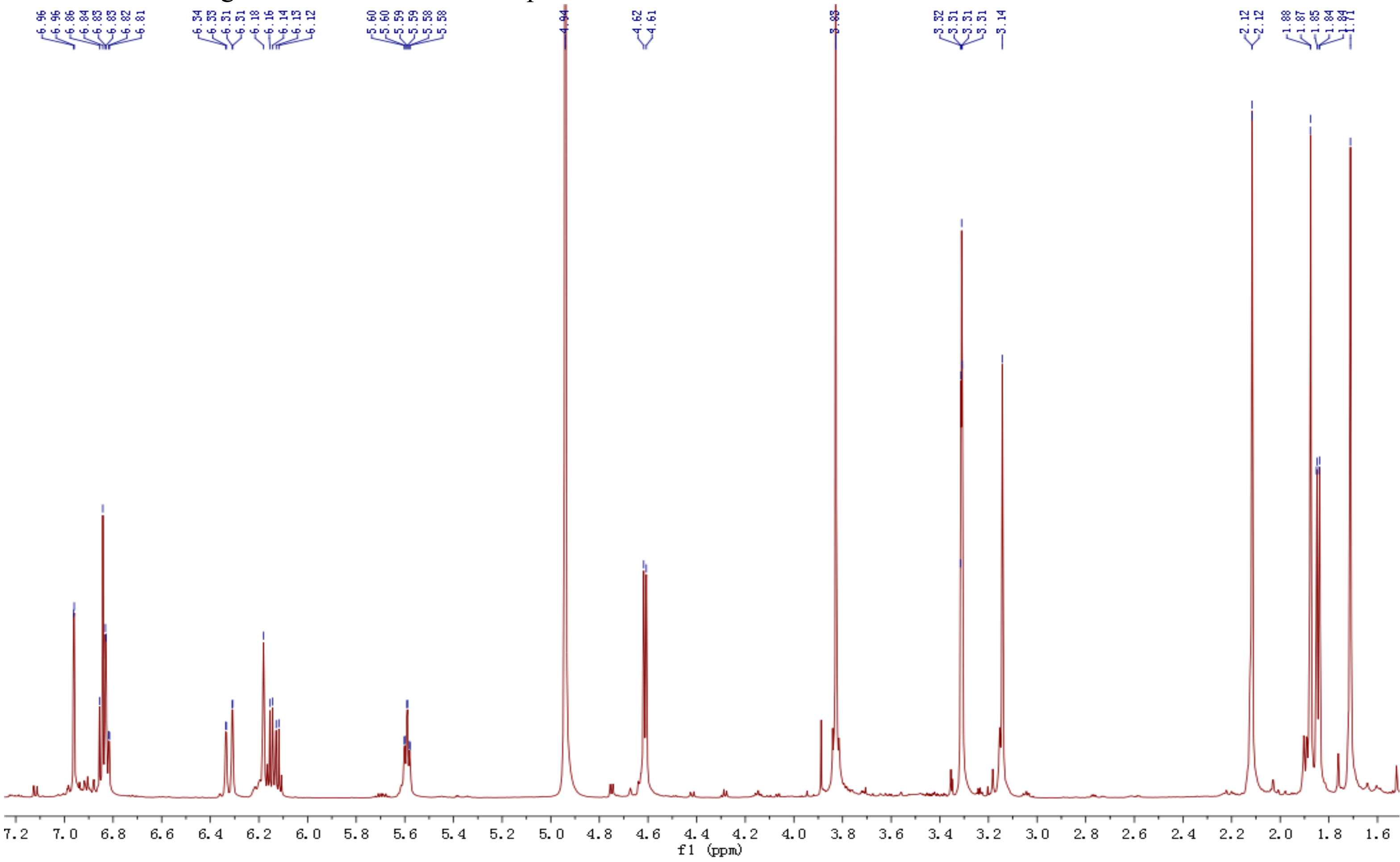

Figure 50S. ^{13}C NMR and DEPT of compound **7**.

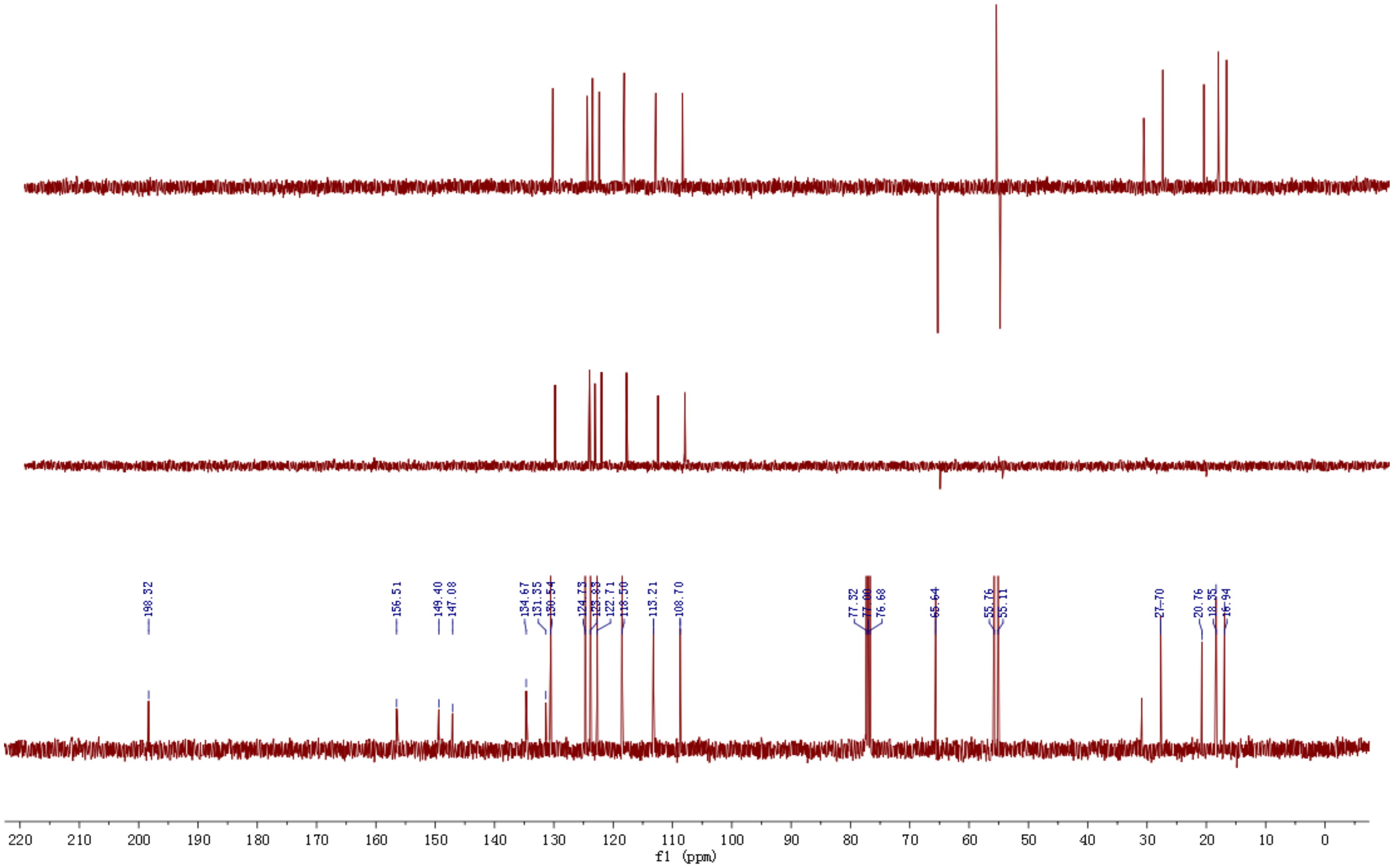

Figure 51S. HSQC of compound **7**.

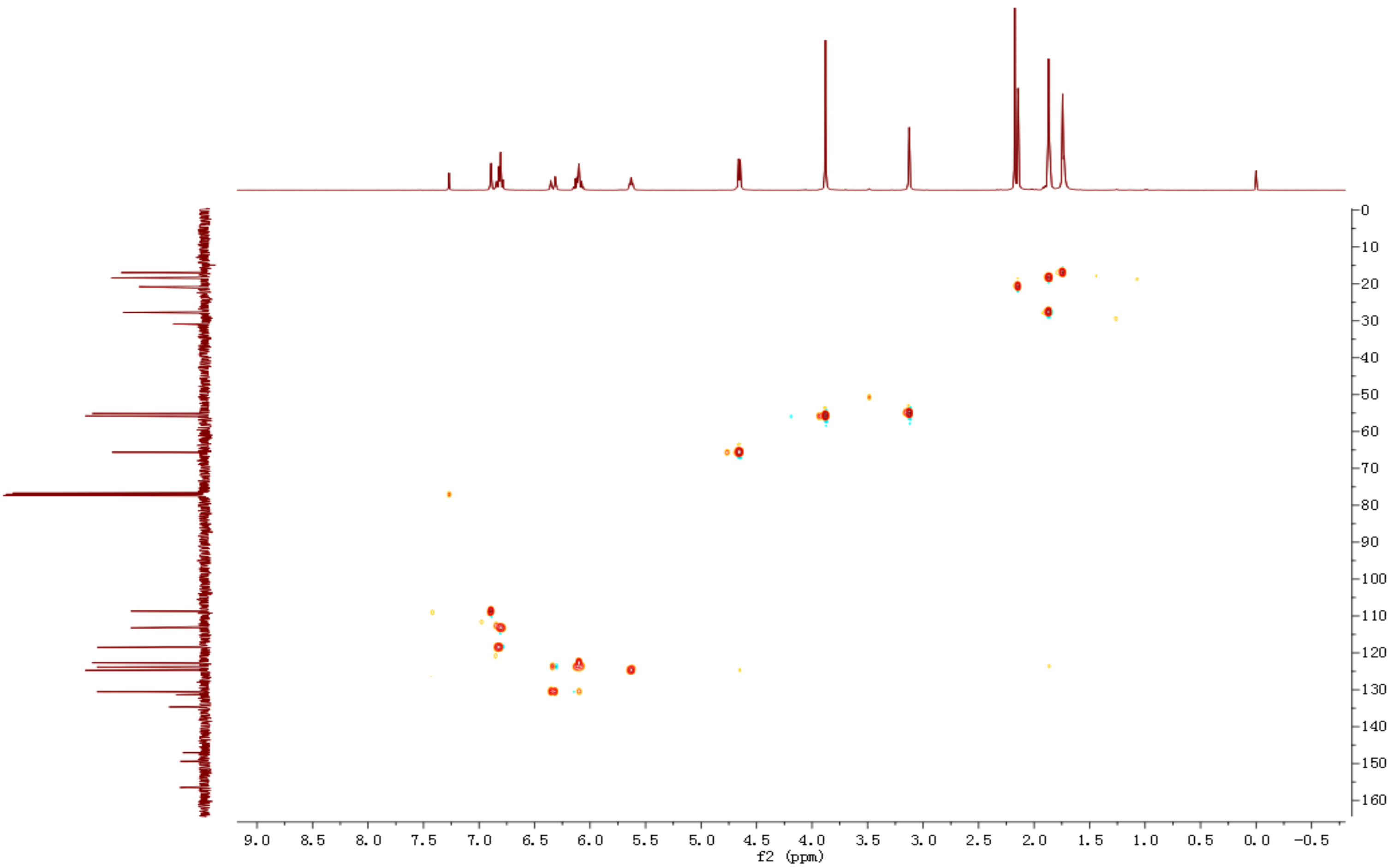

Figure 52S. HMBC of compound **7**.

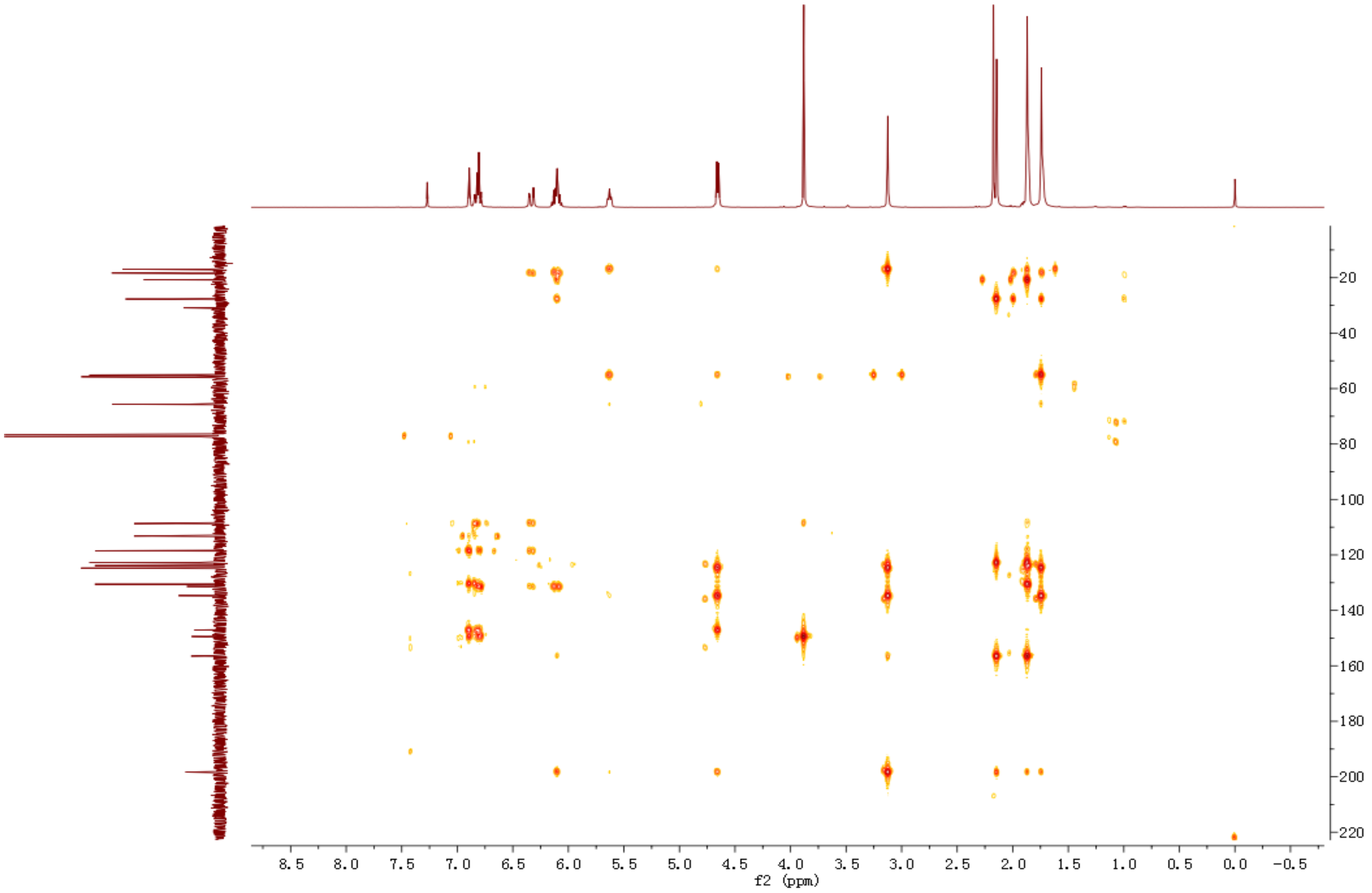

Figure 53S. ^1H-^1H COSY of compound **7**.

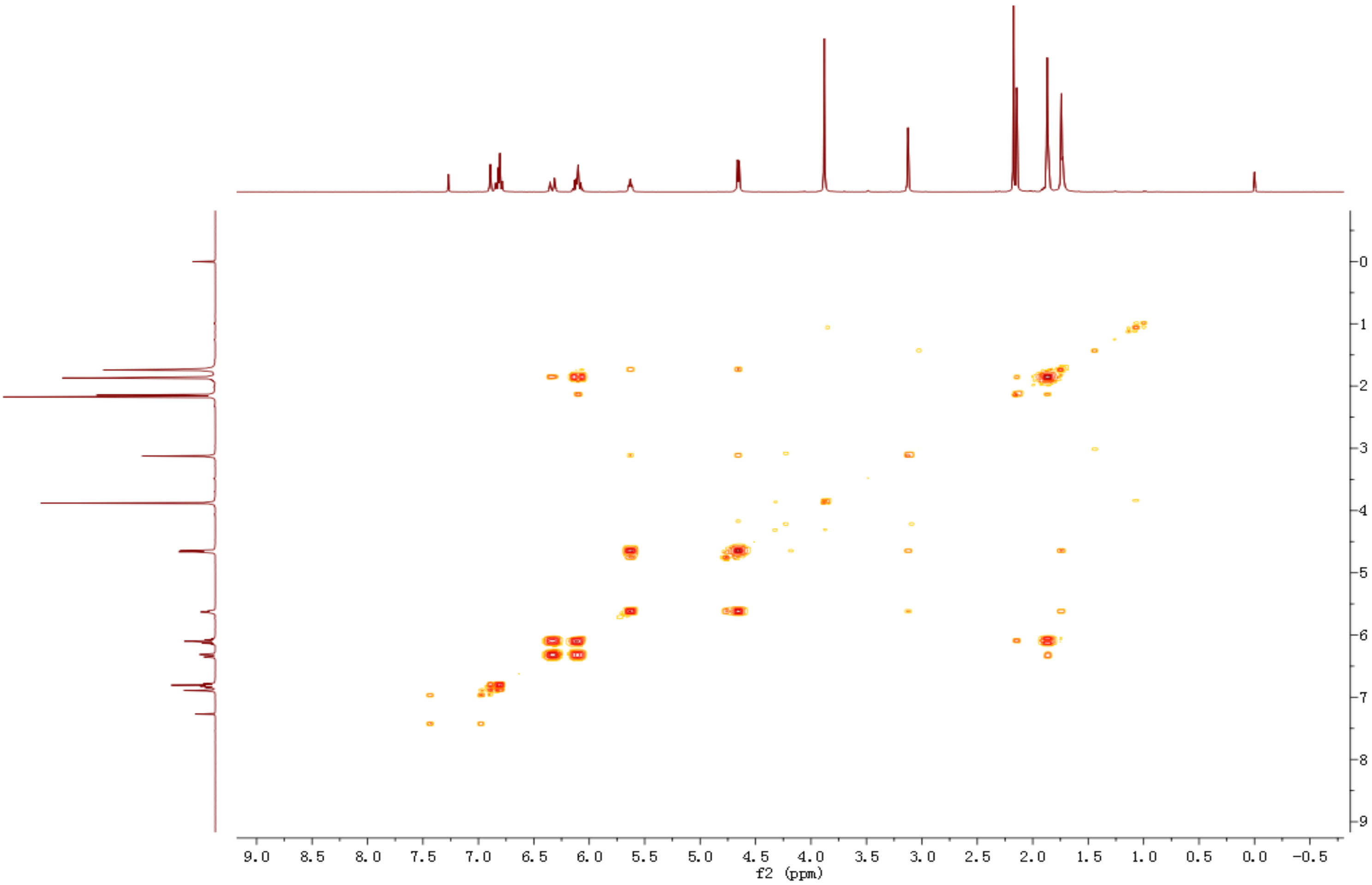

Figure 54S. ROESY of compound **7**.

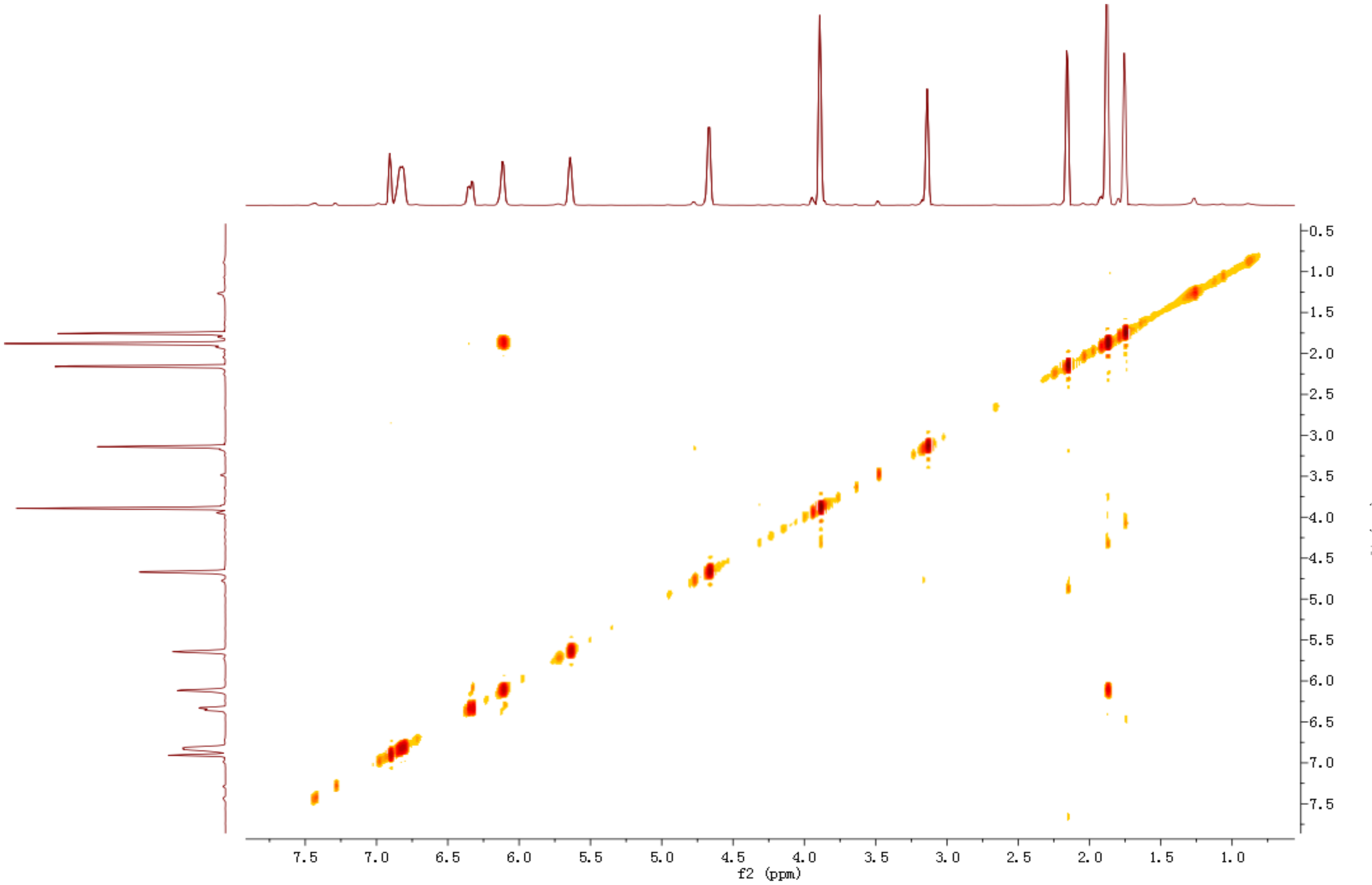

Figure 55S. HRESIMS of compound **7**.

Elemental Composition Report

Single Mass Analysis (displaying only valid results)
Tolerance = 10.0 PPM / DBE: min = 0.5, max = 60.0
Selected filters: None

Monoisotopic Mass, Odd and Even Electron Ions
14 formula(e) evaluated with 1 results within limits (up to 51 closest results for each mass)
Elements Used:
C: 0-200 H: 0-400 O: 3-6

Dgzy-3
11:06:26 09-Apr-2012
Voltage EI+

KIB
M120409EA-02AFAMM 9 (0.826)

Autospec Premier
P776
2.26e4

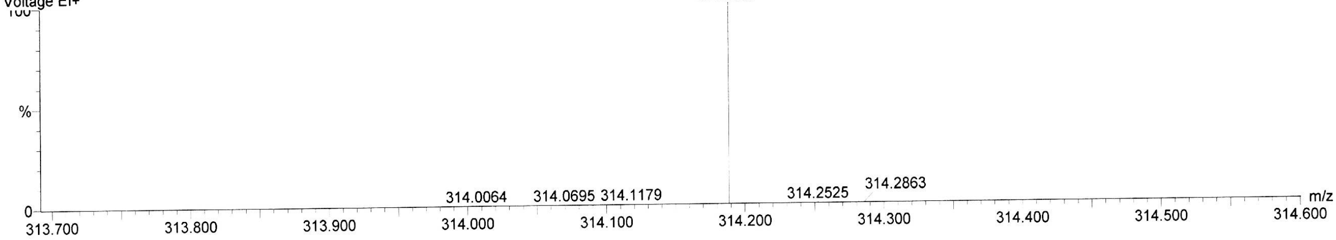

Mass	Calc. Mass	mDa	PPM	DBE	i-FIT	Formula
Minimum:				0.5		
Maximum:		73.0	10.0	60.0		
314.1887	314.1882	0.5	1.6	8.0	5557311.0	C20 H26 O3
